# Sensitivity analysis for contamination in egocentric-network randomized trials with interference

**DOI:** 10.1093/biostatistics/kxag031

**Published:** 2026-08-12

**Authors:** Bar Weinstein, Daniel Nevo

**Affiliations:** Department of Statistics and Operations Research, Tel Aviv University, Tel Aviv, 6997801, Israel; Department of Statistics and Operations Research, Tel Aviv University, Tel Aviv, 6997801, Israel

**Keywords:** causal inference, network experiments, network sampling, probabilistic bias analysis, spillovers

## Abstract

Egocentric-network randomized trials (ENRTs) are increasingly used to estimate causal effects under interference when measuring complete sociocentric network data is infeasible. ENRTs rely on egocentric network sampling, where a set of egos is first sampled, and each ego recruits a subset of its neighbors as alters. Treatments are then randomized across egos. While the observed ego-networks are disjoint by design, the underlying population network may contain edges connecting them, leading to contamination. Under a design-based framework, we show that the Horvitz–Thompson estimators of direct and indirect effects are biased whenever contamination is present. To address this, we derive bias-corrected estimators and propose a novel sensitivity analysis framework based on sensitivity parameters representing the probability or expected number of missing edges. This framework is implemented via both grid sensitivity analysis and probabilistic bias analysis, providing researchers with a flexible tool to assess the robustness of the causal estimators to contamination. We apply our methodology to the HIV Prevention Trials Network 037 study, finding that ignoring contamination may lead to underestimation of indirect effects and overestimation of direct effects.

## 1. INTRODUCTION

In causal inference and design of experiments, it is commonly assumed that there is no interference between units, that is, the treatment assigned to one unit does not affect the outcomes of other units (Cox 1958). However, researchers are often interested in settings where the treatment of interest can spill over from treated units to untreated ones, leading to interference between units. For example, in infectious disease epidemiology, vaccinating some individuals may protect unvaccinated individuals (Halloran and Struchiner 1991).

To estimate causal effects under interference, researchers often rely on detailed information about the network structure that encodes the relationships between units. Such data is typically assumed to be on a complete *sociocentric network*, where all units in the network and their connections are observed (Hudgens and Halloran 2008; Aronow and Samii 2017; Forastiere et al. 2021; Tchetgen Tchetgen et al. 2021; Ogburn et al. 2024). This finite population can be composed of a collection of disjoint clusters or a single connected network. However, collecting complete sociocentric network data is often logistically challenging and expensive, limiting the application of network-based causal inference methods or resorting to subnetworks sampled from the population network.

A common sampling design is *egocentric network sampling* (Marsden 2002; Perry et al. 2018), where a set of egos (also known as indexes) are sampled from the population, and each ego recruits a subset of its neighbors in the population network as alters (non-indexes). The observed network is then assumed to be composed of disjoint ego-networks comprising each ego and its recruited alters. This design is attractive due to its logistical feasibility and accessibility. Such egocentric sampling can be combined with randomized experiments, where treatments are randomly assigned to the recruited egos, resulting in an *egocentric-network randomized trial* (ENRT) (Buchanan et al. 2018, 2024; Chao et al. 2025; Fang et al. 2025). ENRTs are increasingly used in various studies, such as HIV prevention (Latkin et al. 2009, 2013; Booth et al. 2016; Miller et al. 2018), substance addiction (Sherman et al. 2009), and mental health (Asmus et al. 2017; Desrosiers et al. 2020).

However, while most methods assume that the ego-networks are disjoint, alters may be connected to multiple egos in the population network, and egos may be connected to other egos, resulting in contamination between ego-networks. Thus, the egocentric sampling process may lead to missing edges in the observed network, resulting in misspecification of the network interference structure (Weinstein and Nevo 2026). Previous analyses of ENRTs typically assumed that the ego-networks are *non-overlapping* in the population network (Buchanan et al. 2018, 2024; Chao et al. 2025; Fang et al. 2025). That is, alters are connected only to one ego, and egos are not connected to other egos. Such an assumption may not hold in practice and be difficult to verify with the observed data alone, absent additional validation studies or reference data.

In this paper, we relax the nonoverlapping networks assumption and allow for the possibility of overlapping ego-networks. Under a design-based inference paradigm, where the only source of randomness is the treatment assignment, we show that the Horvitz–Thompson (HT) estimators of the indirect and direct effects based on the observed ENRT data are biased whenever there is contamination between ego-networks. We develop a sensitivity analysis framework to assess the impact of such contamination on the causal estimates, and we couple it with bias-corrected estimators of the indirect and direct effects.

These bias-corrected estimators rely on sensitivity parameters that encode the probability or expected number of missing alter–ego and ego–ego edges in the population network. Crucially, because we treat the underlying population network as fixed but partially unobserved, these edge probabilities do not imply a stochastic superpopulation model of network generation. Rather, they quantify the researcher’s epistemic uncertainty regarding latent parts of the interference network. For the direct effect on egos, the framework requires an additional sensitivity parameter capturing the ratio of the sample average direct effect among egos exposed to at least one treated neighbor to that of unexposed egos. We provide practical guidelines for specifying these parameters under various scenarios using covariates and prior knowledge. Furthermore, while our framework is designed to evaluate robustness across various contamination scenarios, we also demonstrate how these sensitivity parameters can be directly calibrated using internal validation data, such as recall surveys in follow-up. This approach reduces the reliance on pure sensitivity analysis and can guide data collection strategies in future trials.

To improve asymptotic precision without compromising design-based validity, we augment these bias-corrected estimators with working outcome regression models. We further develop a conditional two-fold cross-fitting procedure tailored to the ENRT design. The overall framework is implemented using either grid sensitivity analysis (GSA) (Rosenbaum and Rubin 1983; Greenland 1996) or a probabilistic bias analysis (PBA) (Greenland 2005; Lash et al. 2014; Fox et al. 2021).

The HPTN 037 study (Latkin et al. 2009) serves as a primary motivating example for our work. HPTN 037 was an ENRT that aimed to evaluate the efficacy of a peer education intervention to reduce HIV risk behaviors among people who inject drugs. In the trial, treated egos received training on the prevention of risky behaviors and were encouraged to share this information with their alters. While previous analyses of this study (Latkin et al. 2009; Buchanan et al. 2018; Aroke et al. 2023; Chao et al. 2025) assumed that ego-networks are disjoint, contamination remains a significant concern. Specifically, alters might inject drugs or have sexual relations with egos from other ego-networks, or egos might be connected to each other. In this paper, we relax the assumption of disjoint ego-networks and assess the robustness of the causal estimates to contamination between the ego-networks. Our analysis suggests that ignoring contamination may lead to underestimation of the indirect effect and overestimation of the direct effect.

Our work relates to several strands of literature. First, it contributes to the growing literature on causal inference under interference where the true network structure is latent and only proxies are observed by the researchers (Toulis and Kao 2013; Hardy et al. 2019; Li et al. 2021; Boucher and Houndetoungan 2022; Weinstein and Nevo 2025). Second, it adds to the literature on interference with sampled network data which has primarily focused on random node sampling or snowball sampling designs under linear-in-means parametric models (Chandrasekhar and Lewis 2011; Sewell 2017; Yauck 2022; Marray 2024). Third, it contributes to the literature on sensitivity analysis under interference, which has focused on unmeasured confounding (VanderWeele et al. 2014), or on settings where interference depends on two networks, but only one of which is observed (Egami 2021). Finally, it relates to the literature on cross-cluster contamination in cluster-randomized trials, which often recommend changing the cluster boundaries to reduce contamination (Hayes and Moulton 2017), or more recently, develop data-driven methods for cluster construction (Leung 2025).

The rest of the paper is organized as follows. In Section 2, we introduce the setup of ENRTs, define the causal estimands of interest, and show that the commonly used HT estimators based on the observed data are biased under contamination. In Section 3, we develop a sensitivity analysis framework for contamination between ego-networks, providing bias-corrected estimators of the indirect and direct effects. In Section 4, we illustrate the proposed framework via simulations. In Section 5, we re-analyze the HPTN 037 study, assessing the robustness of the causal estimates to possible contamination. Finally, we conclude with a discussion in Section 6. All proofs are provided in the Supplementary Material. The **R** package ENRTsensitivity that implements the proposed sensitivity analysis method is available at https://github.com/barwein/ENRTsensitivity.

## 2. ENRTs

### 2.1. Setup and recruitment process

Let $ \boldsymbol{G}\,=\,(\boldsymbol{V},\boldsymbol{E}) $ denote the population network, where $ \boldsymbol{V}=\{1 , \ldots, N\} $ is the set of units, and $ \boldsymbol{E} $ is the set of edges, assumed to be binary. The size of the population * N * is assumed to be finite but unknown. The population network $ \boldsymbol{G} $ can be described by its adjacency matrix $ \boldsymbol{A} $, where $ A_{ij}\,=\,1 $ if edge $ (i, j)\in\boldsymbol{E} $, and 0 otherwise. We assume that the population network is undirected and that there are no self-edges, that is, $ A_{ij}=A_{ji} $ and $ A_{ii}\,=\,0 $ for all * i *.

Units are recruited to the study via an *egocentric sampling design*. The sampling process consists of two stages. In the first stage, a set of egos (indexes) are sampled from the population. Then, in the second stage, each ego recruits a subset of its neighbors in the population network as alters (non-indexes). Formally, the recruitment process is as follows:

1. Sample egos from the population. Denote the set of recruited egos by $ \mathcal{R}_{e}\subset\boldsymbol{V} $, and $ n_{e}=|\mathcal{R}_{e}| $ as the number of egos. We do not impose any restrictions on the sampling process of egos.
2. Each ego recruits alters from its neighbors in the population network that were not recruited as egos in the first stage. We assume that each alter is recruited by a single ego, resulting in $ n_{e} $ disjoint ego-networks. This is often enforced in the recruitment process by researchers to avoid overlaps between the observed ego-networks, e.g., as was the case in the HPTN 037 study (Latkin et al. 2009). Denote the set of recruited alters by $ \mathcal{R}_{a}\subset\boldsymbol{V}\setminus\mathcal{R}_{e} $, and let $ n_{a}=|\mathcal{R}_{a}| $ be their number.

The set of all recruited units is $ \mathcal{R}=\mathcal{R}_{e}\cup\mathcal{R}_{a} $, yielding an egocentric sample of size $ n\,=\,n_{e}+n_{a} $. We assume that this sample is a strict subset of the population, i.e., $ n\,\lt\,N $. The observed network $ \widetilde{\boldsymbol{G}}\,=\,(\mathcal{R},\widetilde{\boldsymbol{E}}) $ is a subgraph of the population network $ \boldsymbol{G} $ composed of $ n_{e} $ disjoint ego-networks. The set of observed edges is $ \widetilde{\boldsymbol{E}}=\left\{(i, j)\in\boldsymbol{E}:i\in\mathcal{R}_{e},j\in\mathcal{R}_{a},e(j)=i\right\} $, where $ e(j)\in\mathcal{R}_{e} $ is the ego that recruited alter $ j\in\mathcal{R}_{a} $. Clearly, $ \widetilde{\boldsymbol{E}}\subset\boldsymbol{E} $. Let $ \widetilde{\boldsymbol{A}} $ be the adjacency matrix of the observed network $ \widetilde{\boldsymbol{G}} $.

**Figure 1:**
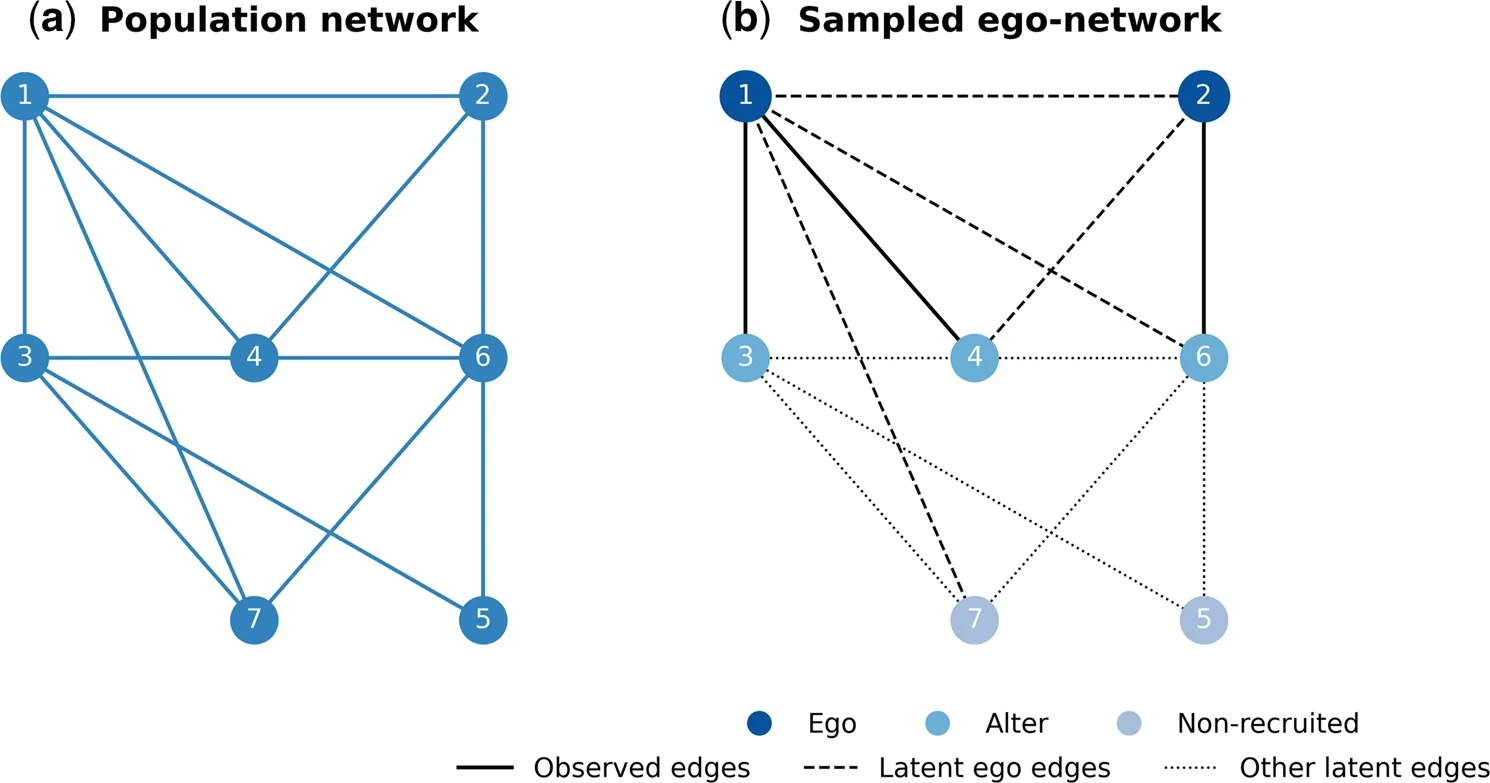
Illustration of egocentric sampling. Panel a) shows the full $ N\,=\,7 $ population network $ \boldsymbol{G} $. Panel b) illustrates a single egocentric sample $ \widetilde{\boldsymbol{G}} $: each ego (nodes 1 and 2; $ n_{e}\,=\,2 $) reports only a subset of its true neighborhood as alters (nodes 3, 4, and 6; $ n_{a}\,=\,3 $). The observed network is composed of disjoint ego–networks (solid lines), while ego–ego and unreported ego–alter connections remain latent (dashed lines). All other edges are also unobserved (dotted lines).

Figure 1 illustrates the egocentric sampling process in a small hypothetical population. Although the sampled egos are connected and some alters are connected to multiple egos in the population network, each ego reports only a subset of its true neighborhood, yielding an observed network made up of disjoint ego-networks.

### 2.2. Experimental design, potential outcomes, and causal estimands

In what follows, all assumptions, estimands, and estimators are conditioned on the recruited sample. Specifically, we consider in-sample causal effects only. Let $ \boldsymbol{Z} $ be the binary treatment assignment vector, where $ Z_{i}\,=\,1 $ if unit * i * is treated, and $ Z_{i}\,=\,0 $ otherwise. We assume that treatments are randomly assigned to the recruited egos via Bernoulli randomization.

Assumption 1 **(Bernoulli experimental design).** Treatments are independently assigned only to recruited egos, $ \Pr(Z_{i}\,=\,1\mid\mathcal{R})=p_{z}\mathbb{I}\{i\in\mathcal{R}_{e}\} $ with a known probability $ p_{z}\in(0,1) $.

Denote the treatment space given the sample as $ \mathcal{Z}_{\mathcal{R}}=\left\{\boldsymbol{z}\in\{0,1\}^{N}:\Pr(\boldsymbol{Z}=\boldsymbol{z}\mid\mathcal{R})\,\gt\,0\right\} $, where treatments of nonrecruited units are assumed to be equal to 0 with probability 1. To avoid clutter, the conditioning on $ \mathcal{R} $ includes the specification of egos and alters sets as well. Furthermore, the distribution of $ \boldsymbol{Z} $ always refers to the conditional distribution of $ \boldsymbol{Z} $ given the recruitment $ \mathcal{R} $.

Let $ Y_{i}(\boldsymbol{z}) $ be the potential outcomes of each unit under treatment vector $ \boldsymbol{z}\in\mathcal{Z}_{\mathcal{R}} $. We take a design-based approach, where the potential outcomes are assumed to be fixed, and the only source of randomness is the treatment assignment. Let $ \boldsymbol{Z}_{-i} $ denote the treatment assignment of all units except unit * i *. We assume that $ \boldsymbol{Z}_{-i} $ affects the potential outcomes of unit * i * only through the values of the following exposure mapping (Manski 2013; Aronow and Samii 2017).

Assumption 2 **(Exposure mapping).** Let $ F(\boldsymbol{Z}_{-i},\boldsymbol{A})=\mathbb{I}\left\{\sum_{j\neq i}Z_{j}A_{ij}\,\gt\,0\right\} $. For any $ \boldsymbol{z},\boldsymbol{z}^{\prime}\in\mathcal{Z}_{\mathcal{R}} $, if $ z_{i}=z^{\prime}_{i} $ and $ F(\boldsymbol{z}_{-i},\boldsymbol{A})=F(\boldsymbol{z}^{\prime}_{-i},\boldsymbol{A}) $, then $ Y_{i}(\boldsymbol{z})=Y_{i}(\boldsymbol{z}^{\prime}) $.

Denote the exposures in the true population network by $ F_{i}=F(\boldsymbol{Z}_{-i},\boldsymbol{A}) $. The exposure $ F_{i} $ indicates whether at least 1 of the neighbors of unit * i * is treated. Assumption 2 implies that the exposure mapping is correctly specified (Sävje 2024), and that only treatments of neighbors affect the potential outcomes of unit * i *. Binary exposure mapping specifications are common in the ENRT literature (Buchanan et al. 2018; Chao et al. 2025; Fang et al. 2025). For clarity, we present the main results under this binary specification, but extend our framework to a three-level exposure mapping specification distinguishing between zero, one, or two-or-more treated neighbors in Supplement B. By Assumption 2, we can express the potential outcomes as $ Y_{i}(\boldsymbol{Z})=Y_{i}(Z_{i},F_{i}) $. Consequently, each unit has four effective potential outcomes, $ Y_{i}(z, f) $ for $ z, f\in\{0,1\}^{2} $, indicating whether unit * i * is treated and whether at least 1 of its neighbors is treated. We assume that the observed outcomes $ Y_{i},\; i\in\mathcal{R} $ are generated from one of the potential outcomes.

Assumption 3 **(Consistency).** For all $ i\in\mathcal{R} $, the observed outcome is given by

\begin{align*} Y_{i}=\sum\limits_{z, f\in\{0,1\}^{2}}Y_{i}(z, f)\mathbb{I}\left\{Z_{i}=z, F_{i}=f\right\}.\end{align*}

In addition, we will also consider cases where covariates $ \boldsymbol{X}_{i} $ are observed for each recruited unit. We will clarify their role later on.

We consider two types of causal estimands, separate for egos and alters. The first is the sample average indirect effect of treatment on the alters,


(1) \begin{align*} IE=\frac{1}{n_{a}}\sum_{i\in\mathcal{R}_{a}}\left[Y_{i}(0,1)-Y_{i}(0,0)\right].\end{align*}

The * IE * is the average effect on alters of being exposed to at least one treated neighbor compared to not being exposed. In Supplement A.9, we also consider the indirect effect on the relative risk scale, for binary outcomes. The second estimand is the sample average direct effect of treatment on the egos,


(2) \begin{align*} DE=\frac{1}{n_{e}}\sum_{i\in\mathcal{R}_{e}}\left[Y_{i}(1,0)-Y_{i}(0,0)\right].\end{align*}

The * DE * is the average effect on egos of being treated compared to not being treated when not exposed to any treated neighbors. Our explicit separation of causal estimands for egos and alters resolves interpretation issues of other estimands (Crawford et al. 2019). Furthermore, by not conflating the two groups, we avoid imposing additional assumptions and structure, as the recruitment process described in Section 2.1 may induce different selection mechanisms for egos and alters, leading to distinct potential outcome means across the two groups.

### 2.3. Naive estimation and bias under contamination

The observed data consist of the treatment assignments $ \boldsymbol{Z} $, the outcomes $ Y_{i} $, the observed network $ \widetilde{\boldsymbol{A}} $, and possibly covariates $ \boldsymbol{X}_{i} $ of the recruited units $ i\in\mathcal{R} $. By Assumption 3, the observed outcomes $ Y_{i} $ are generated from the potential outcomes with exposures $ F_{i} $ created by the *population network* $ \boldsymbol{A} $. However, the observed network $ \widetilde{\boldsymbol{A}} $ might not include all the relevant edges present in the population network $ \boldsymbol{A} $ due to the egocentric sampling process, resulting in a *misspecified network interference structure* (Weinstein and Nevo 2026). Let $ \widetilde{F}_{i}=F(\boldsymbol{Z}_{-i},\widetilde{\boldsymbol{A}}) $, be the observed exposures. Due to this network misspecification, the observed exposures $ \widetilde{F}_{i} $ may differ from the true exposures $ F_{i} $. Specifically, under Assumption 2, only missing ego–ego and alter–ego edges induced by the sampling process can lead to incorrect observed exposures.

Consider, for example, the context of Fig. 1. If only ego 1 is treated, for alter 6 we observe exposure $ \widetilde{F}_{6}\,=\,0 $ since its ego in the observed network (node 2) is not treated. However, its true exposure is $ F_{6}\,=\,1 $ due to its link to ego 1 in the population network. Similarly, under the same treatment assignment, we will observe for ego 2 exposure $ \widetilde{F}_{2}\,=\,0 $ while $ F_{2}\,=\,1 $ in the population network since ego 2 is connected to ego 1.

Due to the ENRT design, the observed exposures of egos are $ \widetilde{F}_{i}\,=\,0 $ for all $ i\in\mathcal{R}_{e} $, while the observed exposures of alters can be either 0 or 1, depending on their observed ego’s treatment status. Specifically, by Assumption 1, alters are exposed to a treated ego in the observed network with probability $ \Pr(\widetilde{F}_{i}\,=\,1\mid i\in\mathcal{R}_{a})=p_{z} $.

In practice, researchers estimate causal effects only with the observed exposures. The most common estimator in design-based inference settings is the HT estimator. For the indirect effect (1), its form is


(3) \begin{align*}\widehat{IE}=\frac{1}{n_{a}}\sum_{i\in\mathcal{R}_{a}}\left[\frac{\mathbb{I}\{\widetilde{F}_{i}\,=\,1\}Y_{i}}{p_{z}}-\frac{\mathbb{I}\{\widetilde{F}_{i}\,=\,0\}Y_{i}}{1-p_{z}}\right],\end{align*}

and for the direct effect (2),


(4) \begin{align*}\widehat{DE}=\frac{1}{n_{e}}\sum_{i\in\mathcal{R}_{e}}\left[\frac{\mathbb{I}\{Z_{i}\,=\,1\}Y_{i}}{p_{z}}-\frac{\mathbb{I}\{Z_{i}\,=\,0\}Y_{i}}{1-p_{z}}\right].\end{align*}

If some alter–ego or ego–ego edges are missing in the observed network $ \widetilde{\boldsymbol{A}} $, both estimators (3) and (4) might be biased.

Proposition 1 Under ENRT design and Assumptions 1 to 3,

\begin{align*}\mathbb{E}_{\boldsymbol{Z}}\left[\widehat{IE}\right] &=IE+\frac{1}{n_{a}}\sum_{i\in\mathcal{R}_{a}}\frac{p_{z}-\pi_{i} ^{a}}{1-p_{z}}\left[Y_{i}(0,1)-Y_{i}(0,0)\right],\\ \mathbb{E}_{\boldsymbol{Z}}\left[\widehat{DE}\right]& =DE+\frac{1}{n_{e}}\sum_{i\in\mathcal{R}_{e}}\pi_{i}^{e}\left\{\left[Y_{i}(1,1)-Y_{i}(0,1)\right]-\left[Y_{i}(1,0)-Y_{i}(0,0)\right]\right\},\end{align*}

where $ \pi_{i}^{a}=\Pr(F_{i}\,=\,1\mid i\in\mathcal{R}_{a}) $ and $ \pi_{i}^{e}=\Pr(F_{i}\,=\,1\mid i\in\mathcal{R}_{e}) $ are the probabilities that an alter and an ego, respectively, are exposed to at least one treated neighbor in the population network $ \boldsymbol{A} $.

When all alters are connected only to their own ego, then $ \pi_{i}^{a}=p_{z} $ for all $ i\in\mathcal{R}_{a} $, and $ \widehat{IE} $ is an unbiased estimator of * IE *. Otherwise, whenever some alters are connected to more than one ego in the population network, $ \pi_{i}^{a} > p_{z} $, and the indirect effect estimator (3) based on the observed exposures can be biased. The magnitude of the bias depends on the number of egos each alter is connected to in the population network. That is, the more egos an alter is connected to, the larger the gap between $ \pi_{i}^{a} $ and $ p_{z} $, and hence the larger the bias. In addition, we show in Supplement A.5 that under unit-level monotonicity (in either direction) of the potential outcomes $ Y_{i}(0,1) $ and $ Y_{i}(0,0) $, the naive indirect estimator $ \widehat{IE} $ is biased toward the null of no indirect effect.

If an ego is not connected to any other egos in the population network, then $ \pi_{i}^{e}\,=\,0 $. If this is the case for all $ i\in\mathcal{R}_{e} $, then $ \widehat{DE} $ is an unbiased estimator of (2). However, if at least one ego * i * is connected to at least one other ego in the population network, then $ \pi_{i}^{e}\,\gt\,0 $, and the direct effect estimator $ \widehat{DE} $ based on the observed data can be biased. Similarly to alters, the magnitude of the bias depends on the number of ego–ego edges in the population network. Furthermore, in Supplement A.6, we analyze the direction of the bias of $ \widehat{DE} $ relative to the null of no direct effect. We demonstrate that the direction of the bias is governed by the ratio of the sample average direct effect among egos exposed to at least one treated neighbor to the effect among non-exposed egos. Generally, if the direct effects are larger, on average, in the presence of treated neighbors than in their absence, $ \widehat{DE} $ is biased away from the null. Conversely, if the direct effects are smaller, on average, in the presence of treated neighbors, the estimator is biased toward the null.

## 3. SENSITIVITY ANALYSIS FOR CONTAMINATION

Proposition 1 shows that the HT estimators based on the observed data are biased whenever there is contamination between the ego-networks. We refer to contamination as the presence of alter–ego and ego–ego edges in the population network $ \boldsymbol{A} $, that are missing from the observed network $ \widetilde{\boldsymbol{A}} $ due to the egocentric sampling process. Under such contamination, the ego-networks are not disjoint in the population network, leading to incorrect exposures when computed using the observed network.

By the structure of the egocentric sampling and the exposure mapping specification (Assumption 2), alters with observed exposure $ \widetilde{F}_{i}\,=\,1 $ will have correctly classified exposures. That is, $ \widetilde{F}_{i}\,=\,1 $ implies $ F_{i}\,=\,1 $ with probability one for $ i\in\mathcal{R}_{a} $. However, alters with $ \widetilde{F}_{i}\,=\,0 $ can have a true exposure of $ F_{i}\,=\,1 $ or $ F_{i}\,=\,0 $, depending on the structure of the population network $ \boldsymbol{A} $. Therefore, the observed outcomes $ Y_{i} $ for alters with $ \widetilde{F}_{i}\,=\,0 $ can be generated from either $ Y_{i}(0,1) $ or $ Y_{i}(0,0) $.

On the other hand, all egos have observed exposures of $ \widetilde{F}_{i}\,=\,0 $, as they are linked only to alters in their ego-network in the observed network $ \widetilde{\boldsymbol{A}} $. However, the observed outcomes of egos that are assigned to treatment $ Z_{i}=z $ can be generated from $ Y_{i}(z , 1) $ or $ Y_{i}(z , 0) $, implying that observed outcomes of egos are possibly linked to four different potential outcomes, compared to only two among the alters.

We develop a sensitivity analysis framework that enables researchers to assess the impact of possible contamination between ego-networks on the causal estimates. In Sections 3.1 and 3.2, we provide bias-corrected estimators of the indirect and direct effects that account for possible exposure misspecification due to the egocentric-network recruitment process. Furthermore, in Section 3.3, we show how to augment these estimators with an outcome model through a cross-fitting procedure tailored to the design-based framework taken in this paper, enabling researchers to integrate covariates into the analysis to improve efficiency. Such model-assisted estimation is common in survey sampling (Särndal et al. 2003) and causal inference (Ding 2024).

Building on Proposition 1, the bias-corrected indirect and direct effect estimators are functions of the exposure probabilities under the true population network $ \pi_{i}^{a} $ and $ \pi_{i}^{e} $, respectively. The structure of the egocentric sampling design and our setup enables us to decompose $ \pi_{i}^{a} $ and $ \pi_{i}^{e} $ into basic components that are straightforward to compute given the specification of the presumed *expected number* or the *probability* of missing alter–ego and ego–ego edges. These components will form the basis of our sensitivity parameters, enabling seamless integration of covariates and prior knowledge into the sensitivity analysis. Crucially, we maintain the design-based framework throughout, treating the underlying population network $ \boldsymbol{A} $ as fixed but partially unobserved. Therefore, probability statements regarding latent edges do not reflect a stochastic superpopulation model or assumptions about a data-generating process. Rather, they quantify the researcher’s epistemic uncertainty regarding the fixed, unobserved edges in the interference network. We provide multiple examples and practical guidelines on how to specify these parameters in Section 3.4.

For the direct effect, we require an additional sensitivity parameter to account for the complexity resulting from possibly four potential outcomes generating the observed outcomes. This parameter is the ratio of the sample average direct effect among egos exposed to at least one treated neighbor to the effect among non-exposed egos, as described in Section 3.2.

While the primary focus of this paper is to assess the robustness of the estimates over plausible contamination scenarios, researchers can sometimes calibrate the sensitivity parameters from auxiliary information. In Section 3.6, we demonstrate how different types of internal validation data, such as recall surveys at follow-up, can be leveraged to estimate these parameters. This approach reduces the reliance on pure sensitivity analysis and can motivate data collection and design choices in future studies.

### 3.1. Indirect effect

Define the bias-corrected indirect effect estimator as a function of the observed data and $ \pi_{i}^{a} $,


(5) \begin{align*}\widehat{IE}_{adj}=\frac{1}{n_{a}}\sum_{i\in\mathcal{R}_{a}}\frac{1-p_{z}}{1-\pi_{i}^{a}}\left[\frac{\mathbb{I}\{\widetilde{F}_{i}\,=\,1\}Y_{i}}{p_{z}}-\frac{\mathbb{I}\{\widetilde{F}_{i}\,=\,0\}Y_{i}}{1-p_{z}}\right].\end{align*}

Given $ \pi_{i}^{a} $, (5) is an unbiased estimator of the indirect effect (1).

Proposition 2 Under ENRT design and Assumptions 1 to 3, we have $ \mathbb{E}_{\boldsymbol{Z}}\big[\widehat{IE}_{adj}\big]=IE $.

By the ENRT design and Assumption 2, $ \pi_{i}^{a}\geq p_{z} $, with equality if and only if there are no additional latent alter–ego edges for alter * i *. Consequently, if all alters have the same exposure probability, $ \pi^{a}_{i}=\pi^{a} $, then $ \widehat{IE}_{adj}=\frac{1-p_{z}}{1-\pi^{a}}\widehat{IE} $. Therefore, $ \mathrm{sign}\big(\widehat{IE}_{adj}\big)=\mathrm{sign}\big(\widehat{IE}\big) $, and $ \widehat{IE}_{adj}\geq\widehat{IE} $ if $ \widehat{IE}\,\gt\,0 $ and vice versa otherwise.

We derive variance estimators for the bias-corrected estimator $ \widehat{IE}_{adj} $. By the ENRT design, alters in the same ego-network all have the same treatment assignments and observed exposure status. Thus, we can calculate the variance by using the aggregated outcomes in each ego-network. Let $ r^{a}_{i}=\frac{1-p_{z}}{1-\pi_{i}^{a}}\left[\frac{\mathbb{I}\{\widetilde{F}_{i}\,=\,1\}Y_{i}}{p_{z}}-\frac{\mathbb{I}\{\widetilde{F}_{i}\,=\,0\}Y_{i}}{1-p_{z}}\right] $ such that $ \widehat{IE}_{adj}=\frac{1}{n_{a}}\sum_{i\in\mathcal{R}_{a}}r^{a}_{i} $. The design-based variance estimator is (Ding 2024)


(6) \begin{align*}\widehat{V}_{\boldsymbol{Z}}\left(\widehat{IE}_{adj}\right)=\frac{1}{n_{a}^{2}}\sum_{i\in\mathcal{R}_{e}}\left(T_{i}-\overline{T}\right)^{2},\end{align*}

where $ T_{i}=\sum_{j\in\mathcal{R}_{a}:e(j)=i}r^{a}_{j} $ and $ \overline{T}=\frac{1}{n_{e}}\sum_{i\in\mathcal{R}_{e}}T_{i} $ is the average over ego-networks. In Supplement A.7, we derive the variance estimator and establish the asymptotic normality of the bias-corrected estimator $ \widehat{IE}_{adj} $, ensuring that Wald-type CIs using the variance estimator $ \widehat{V}_{\boldsymbol{Z}}\left(\widehat{IE}_{adj}\right) $ achieve a valid, albeit conservative, asymptotic coverage rate.

### 3.2. Direct effect

As the observed outcomes of egos are possibly generated from four different potential outcomes, compared to only two for the alters, bias correction for the direct effect estimator requires specifying the relation between the direct effect among nonexposed egos ($ F_{i}\,=\,0 $) and the effect among egos exposed to at least one treated neighbor ($ F_{i}\,=\,1 $). Denote the direct effect of treatment on an ego $ i\in\mathcal{R}_{e} $ with exposure value $ f\in\{0,1\} $ by $ \Delta_{i}(f)=Y_{i}(1, f)-Y_{i}(0, f) $. Let $ \overline{\Delta}(f)=\frac{1}{n_{e}}\sum_{i\in\mathcal{R}_{e}}\Delta_{i}(f) $ denote the sample average effect for $ f\in\{0,1\} $. That is, with a slight abuse of notations, $ \overline{\Delta}(0)=DE $ as defined in (2). Let * κ * be the ratio of the sample average direct effect among egos exposed to at least one treated neighbor $ \overline{\Delta}(1) $ to the effect among non-exposed egos $ \overline{\Delta}(0) $:


(7) \begin{align*}\overline{\Delta}(1)=\kappa\overline{\Delta}(0).\end{align*}

Under the design-based framework taken in the paper, where the potential outcomes are fixed, there is always a constant * κ * satisfying (7). The parameter * κ * will serve as an additional sensitivity analysis parameter. We discuss the interpretation of * κ * below.

Let $ \overline{\pi}^{e}=\frac{1}{n_{e}}\sum_{i\in\mathcal{R}_{e}}\pi_{i}^{e} $ be the sample average probability of exposure among the egos. We define an inflation factor $ u_{e}=n_{e}\left[1+\overline{\pi}^{e}(\kappa-1)\right] $ that adjusts the effective sample size of egos based on the expected contamination and * κ *. Using this factor, we define the bias-corrected direct effect estimator as a function of the observed data, $ \overline{\pi}^{e} $ and * κ *,


(8) \begin{align*}\widehat{DE}_{adj}=\frac{1}{1+\overline{\pi}^{e}(\kappa-1)}\widehat{DE}=\frac{1}{u_{e}}\sum_{i\in\mathcal{R}_{e}}\left[\frac{\mathbb{I}\{Z_{i}\,=\,1\}Y_{i}}{p_{z}}-\frac{\mathbb{I}\{Z_{i}\,=\,0\}Y_{i}}{1-p_{z}}\right].\end{align*}

An additional assumption is needed for $ \widehat{DE}_{adj} $ to be unbiased, as we now formalize. For two vectors $ \boldsymbol{a},\boldsymbol{b}\in\mathbb{R}^{m} $, let the empirical covariance be $ S_{\boldsymbol{a},\boldsymbol{b}}=\frac{1}{m}\sum_{i\,=\,1}^{m}(a_{i}-\overline{a})(b_{i}-\overline{b}) $, where $ \overline{a}=\frac{1}{m}\sum_{i}a_{i} $. To eliminate bias, we require that the empirical covariance between exposure probabilities $ \pi_{i}^{e} $ and the direct effect among exposed egos $ \Delta_{i}(1) $ is equal to the empirical covariance between $ \pi_{i}^{e} $ and the direct effect among non-exposed egos $ \Delta_{i}(0) $. Let $ \boldsymbol{\pi}^{e}=\left(\pi_{1}^{e},\ldots , \pi_{n_{e}}^{e}\right) $ and $ \boldsymbol{\Delta}_{i}(f)=\left(\Delta_{1}(f),\ldots , \Delta_{n_{e}}(f)\right) $ be the vectors of exposure probabilities and direct effects at the unit-level of the egos.

Assumption 4
$ S_{\boldsymbol{\pi}^{e},\boldsymbol{\Delta}(1)}=S_{\boldsymbol{\pi}^{e},\boldsymbol{\Delta}(0)} $ .

To provide a better understanding of the implications of Assumption 4, consider the representation of the potential outcomes under a saturated model


\begin{align*} Y_{i}(z, f)=\beta_{0i}+\beta_{1i}z+\beta_{2i}f+\beta_{3i}zf.\end{align*}

Under this representation, $ \Delta_{i}(1)=\beta_{1i}+\beta_{3i} $, $ \Delta_{i}(0)=\beta_{1i} $, and simple calculations show that Assumption 4 is equivalent to


(9) \begin{align*} S_{\boldsymbol{\pi}^{e},\boldsymbol{\beta}_{3}}=\frac{1}{n_{e}}\sum_{i\in\mathcal{R}_{e}}(\pi_{i}^{e}-\overline{\pi}^{e})(\beta_{3i}-\overline{\beta_{3}})\,=\,0.\end{align*}

That is, the empirical covariance between the exposure probabilities $ \pi_{i}^{e} $ and the treatment-exposure interaction terms $ \beta_{3i} $ is 0. Equation (9) naturally holds if these interaction effects are homogeneous across all egos (ie $ \beta_{3i}=\beta_{3} $ for some constant $ \beta_{3} $) or if the exposure probabilities are homogeneous (ie $ \pi_{i}^{e}=\pi^{e} $ for some constant $ \pi^{e}\in[0,1] $). In practice, this assumption would only be violated if egos that are highly connected to other egos systematically exhibit different interaction responses to the intervention compared to less connected egos.

Given $ \pi_{i}^{e} $ and * κ *, (8) is an unbiased estimator of the direct effect (2).

Proposition 3 Under ENRT design and Assumptions 1 to 4, we have $ \mathbb{E}_{\boldsymbol{Z}}\left[\widehat{DE}_{adj}\right]=DE $.

As described above, under homogeneous exposure probabilities, the empirical covariance requirement stated in Assumption 4 is trivially satisfied.

Corollary 1 If exposure probabilities are homogeneous ($ \pi_{i}^{e}=\pi^{e} $ for all $ i\in\mathcal{R}_{e} $), then $ \mathbb{E}_{\boldsymbol{Z}}\left[\widehat{DE}_{adj}\right]=DE $.

Reasoning about the range of plausible * κ * values depends on the mechanism under study. For example, in vaccine efficacy studies, if treatment represents vaccines, it is reasonable to assume that the direct effect of vaccination is larger, on average, among egos with nonvaccinated neighbors compared to those with vaccinated neighbors (who benefit from some indirect protection), implying * κ < 1 *. On the other hand, in the HPTN 037 study, which we analyze in Section 5, the treatment is a behavioral intervention reliant on peer education. In such settings, researchers may believe the intervention to be mutually reinforcing. That is, the direct effect may be larger, on average, for egos who are also exposed to at least one treated ego compared to those who are not, which implies * κ > 1 *. While researchers must often reason about ranges for * κ * based on subject-matter expertise, it is also possible to estimate * κ * directly if appropriate internal validation data are collected, as we detail in Section 3.6.

If $ \pi_{i}^{e}\,=\,0 $ or * κ = 1 *, the bias-corrected estimator $ \widehat{DE}_{adj} $ reduces to the naive HT estimator $ \widehat{DE} $. Thus, $ \widehat{DE} $ is unbiased if, for example, * κ = 1 * and the probabilities $ \pi_{i}^{e} $ are homogeneous. That is, $ \widehat{DE} $ can be an unbiased estimator of * DE * even if there is some contamination between the egos ($ \pi^{e}\,\gt\,0 $). See Supplement A.4 for more details.

Furthermore, if $ \kappa > 1-\frac{1}{\overline{\pi}^{e}} $ (e.g., for $ \overline{\pi}^{e}\,=\,0.01 $ this implies * κ > -99 *), $ \mathrm{sign}\big(\widehat{DE}_{adj}\big)=\mathrm{sign}\big(\widehat{DE}\big) $, i.e., the bias-corrected estimator will have the same sign as the naive estimator. In addition, if * κ > 1 * or $ \kappa\in(1-\frac{1}{\overline{\pi}^{e}},1) $, the bias-corrected estimator $ \widehat{DE}_{adj} $ is closer to or further away from, respectively, zero than the naive estimator $ \widehat{DE} $. See Supplement A.6 for further details.

The requirement of Assumption 4 in Proposition 3 can be replaced with alternative formulations. We derive nonparametric bounds for * DE * based on the observed data, $ \pi_{i}^{e} $ and * κ *, enabling researchers to obtain valid intervals for * DE * without Assumption 4. Furthermore, we show that replacing the average-level proportionality between $ \overline{\Delta}(1) $ and $ \overline{\Delta}(0) $, as expressed in (7), with a unit-level proportionality assumption between $ \Delta_{i}(1) $ and $ \Delta_{i}(0) $, produces a modified bias-corrected estimator that is an unbiased estimator of * DE * without Assumption 4. See Supplement A.10 for more details.

Our simulation study (Section 4 and Supplement D) assessed the impacts of violating Assumption 4 and misspecifying the sensitivity parameter * κ *. We found that violations of Assumption 4 resulted in negligible bias for the corrected direct effect estimators, albeit with slight variance underestimation. The impact of misspecifying * κ *, however, depends on its direction. If * κ * is misspecified in the wrong direction (e.g., specifying * κ < 1 * when the true * κ > 1 *), the bias-corrected estimator can exhibit greater bias and lower coverage than the naive estimator. Conversely, when * κ * is misspecified in magnitude but correctly oriented in direction, the bias-corrected estimator outperforms the naive estimator, maintaining smaller bias and higher coverage.

We derive a conservative variance estimator for the bias-corrected estimator $ \widehat{DE}_{adj} $. Let $ r^{e}_{i}=\frac{\mathbb{I}\{Z_{i}\,=\,1\}Y_{i}}{p_{z}}-\frac{\mathbb{I}\{Z_{i}\,=\,0\} Y_{i}}{1-p_{z}} $ such that $ \widehat{DE}_{adj}=\frac{1}{u_{e}}\sum_{i\in\mathcal{R}_{e}}r^{e}_{i} $. By the ENRT design, treatment assignment is independent across egos. The Neyman-type variance estimator is therefore (Ding 2024),


\begin{align*}\widehat{V}_{Neyman}\left(\widehat{DE}_{adj}\right)=\frac{1}{u_{e}^{2}}\sum_{i\in\mathcal{R}_{e}}\left(r^{e}_{i}-\widehat{DE}_{adj}\right)^{2}.\end{align*}

This estimator assumes that the terms $ r^{e}_{i} $ are independent. However, in the presence of contamination, this independence assumption is violated. If two egos * i * and * j * share a neighbor $ k\in\mathcal{R}_{e} $ in the population network $ \boldsymbol{A} $, then the terms $ r^{e}_{i} $ and $ r^{e}_{j} $ are not independent. This dependence induces pairwise covariances ($ \mathrm{Cov}_{\boldsymbol{Z}}(r^{e}_{i},r^{e}_{j}) $) that are omitted from $ \widehat{V}_{Neyman}\left(\widehat{DE}_{adj}\right) $. To ensure valid inference, we propose a conservative estimator $ \widehat{V}_{Conta.}\left(\widehat{DE}_{adj}\right) $ for the variance in $ \widehat{DE}_{adj} $ attributed to contamination between ego-networks, derived using the edge probabilities under the sensitivity model (presented in Section 3.4) to account for these latent correlations. The final corrected variance estimator for $ \widehat{DE}_{adj} $ is given by


(10) \begin{align*}\widehat{V}_{\boldsymbol{Z}}\left(\widehat{DE}_{adj}\right)=\widehat{V}_{Neyman}\left(\widehat{DE}_{adj}\right)+\widehat{V}_{Conta.}\left(\widehat{DE}_ {adj}\right).\end{align*}

In Supplement A.7 we provide the derivation of the variance estimator $ \widehat{V}_{\boldsymbol{Z}}\left(\widehat{DE}_{adj}\right) $ and, analogously to Section 3.1, show the asymptotic normality of $ \widehat{DE}_{adj} $ and that Wald-type CIs using the variance estimator $ \widehat{V}_{\boldsymbol{Z}}\left(\widehat{DE}_{adj}\right) $ achieve a valid, albeit conservative, asymptotic coverage rate.

### 3.3. Outcome-model augmented estimators

The bias-corrected estimators $ \widehat{IE}_{adj} $ and $ \widehat{DE}_{adj} $ can be improved by incorporating outcome regression models that leverage information from the covariates $ \boldsymbol{X}_{i} $. Crucially, the incorporation of these models does not constitute a departure from our design-based paradigm. The regression models serve strictly as working models used to improve asymptotic precision, and the theoretical validity of the resulting augmented estimators relies solely on the randomization of the treatment assignment (Lin 2013; Abadie et al. 2020; Gao and Ding 2025). As we demonstrate in our simulation study (Section 4), this augmentation yields valid variance estimates, overcoming the conservative variance estimation of the nonaugmented estimators, while maintaining negligible bias.

To fully preserve this design-based validity, standard regression adjustments require careful implementation. While sample splitting (i.e., cross-fitting) is the standard solution to avoid finite-sample bias, standard splitting schemes can violate the design-based independence assumptions (Lu et al. 2025). To address this, we employ a conditional two-fold cross-fitting procedure tailored to the ENRT design. Specifically, we adapt Algorithm 1 of Lu et al. (2025) to our setting. We describe here the algorithm for completeness, but refer readers to Lu et al. (2025) for additional details. We focus on the augmented bias-corrected estimators, but the naive estimators $ \widehat{IE} $ and $ \widehat{DE} $ can also be augmented similarly, albeit without the bias-correction weighting.

Let $ \widehat{\mu}^{a}_{i}(f) $ denote the predicted value for alter * i * under exposure $ \widetilde{F}_{i}=f $, from an outcome model estimated using covariates $ \boldsymbol{X}_{i} $. For example, $ \widehat{\mu}^{a}_{i}(f) $ can be the predicted value from a linear model of the form $ Y_{i}\sim\widetilde{F}_{i}+\boldsymbol{X}_{i} $. Similarly, let $ \widehat{\mu}^{e}_{i}(z) $ be the predicted value for ego * i * under treatment $ Z_{i}=z $, from an outcome model estimated using covariates $ \boldsymbol{X}_{i} $. The two-fold cross-fitting algorithm is as follows.

1. Randomly split the observed ego-networks into 2 disjoint parts $ S_{0},S_{1} $ such that $ | S_{0}\cup S_{1}|=n $ and $ S_{0}\cap S_{1}=\emptyset $. Specifically, we assign each ego-network (ego and alters) into $ S_{1} $ with probability 0.5.
2. For $ q\,=\,0,1 $:
  a. Estimate the outcome models for alters and egos using data from the complementary split $ S_{1-q} $.
  b. Predict $ \widehat{\mu}^{a}_{i}(f) $ and $ \widehat{\mu}^{e}_{i}(z) $ on the units from $ S_{q} $.
  c. Estimate * IE * on units from $ S_{q} $:
    \begin{align*}\widehat{IE}^{aug}_{adj[q]}=\frac{1}{n_{a[q]}}\sum_{i\in\mathcal{R}_{a}\cap S_ {q}}\frac{1-p_{z}}{1-\pi^{a}_{i}}\left[D^{a}_{i}+\widehat{\mu}^{a}_{i}(1)-\widehat{\mu}^{a}_{i}(0)\right],\end{align*}
    where $ n_{a[q]}=\sum_{i\in\mathcal{R}_{a}}\mathbb{I}\{i\in S_{q}\} $ is the number of alters in split $ S_{q} $, and where
    \begin{align*} D^{a}_{i}=\frac{\mathbb{I}\{\widetilde{F}_{i}\,=\,1\}\left(Y_{i}-\widehat{\mu}^{a} _{i}(1)\right)}{p_{z}}-\frac{\mathbb{I}\{\widetilde{F}_{i}\,=\,0\}\left(Y_{i}-\widehat{\mu}^{a}_{i}(0)\right)}{1-p_{z}}.\end{align*}
  d. Estimate * DE * on units from $ S_{q} $:
    \begin{align*}\widehat{DE}^{aug}_{adj[q]}=\frac{1}{u_{e[q]}}\sum_{i\in\mathcal{R}_{e}\cap S_ {q}}\left[D^{e}_{i}+\widehat{\mu}^{e}_{i}(1)-\widehat{\mu}^{e}_{i}(0)\right],\end{align*}
    where $ u_{e[q]}=n_{e[q]}[1+\overline{\pi}^{e[q]}(\kappa-1)] $ similarly to $ u_{e} $ in Section 3.2, with $ n_{e[q]}=\sum_{i\in\mathcal{R}_{e}}\mathbb{I}\{i\in S_{q}\} $ and $ \overline{\pi}^{e[q]}=\frac{1}{n_{e[q]}}\sum_{i\in\mathcal{R}_{e}\cap S_{q}}\pi_{i}^{e} $ are the number of egos and the average exposure probability, respectively, in split $ S_{q} $, and
    \begin{align*} D^{e}_{i}=\frac{\mathbb{I}\{Z_{i}\,=\,1\}\left(Y_{i}-\widehat{\mu}^{e}_{i}(1)\right)}{p_{z}}-\frac{\mathbb{I}\{Z_{i}\,=\,0\}\left(Y_{i}-\widehat{\mu}^{e}_{i}(0)\right)}{1-p_{z}}.\end{align*}
3. Combine the estimates across splits
  (11) \begin{align*}\widehat{IE}^{aug}_{adj}&=\sum_{q\,=\,0,1}\frac{n_{a[q]}}{n_{a}}\widehat{IE}^{aug}_{adj[q]},\nonumber\\ \widehat{DE}^{aug}_{adj}&=\sum_{q\,=\,0,1}\frac{u_{e[q]}}{u_{e}}\widehat{DE}^{aug}_{adj[q]}.\end{align*}

Variance estimation is a combination across splits of variance estimators (6) for the IE estimator and (10) for the DE estimator (Lu et al. 2025). In Supplement A.8, we show that the augmented estimators $ \widehat{IE}^{aug}_{adj} $ and $ \widehat{DE}^{aug}_{adj} $ remain unbiased estimators of * IE * and * DE *, respectively, derive their variance estimator, and establish their asymptotic normality, ensuring that Wald-type CIs based on their associated variance estimators achieve at least the nominal asymptotic coverage rate.

### 3.4. Specifying the sensitivity parameters

Propositions 2 and 3 establish that the bias-corrected estimators of the indirect and direct effects depend on the unknown exposure probabilities $ \pi_{i}^{a} $ and $ \pi_{i}^{e} $ of alters and egos, respectively, in the population network $ \boldsymbol{A} $. For the direct effect, the bias-corrected estimator also depends on the sensitivity parameter * κ *. We now illustrate how the computation of $ \pi_{i}^{a} $ and $ \pi_{i}^{e} $ reduces to specification of edge-level probabilities or the expected number of missing alter–ego and ego–ego edges. These specifications form the basis of our sensitivity parameters. Importantly, while we maintain the design-based framework, implying that the population network $ \boldsymbol{A} $ is fixed, its full structure is unobserved. Consequently, we treat the presence of missing edges as uncertain for the purpose of the sensitivity analysis. We do not posit a generative superpopulation model for $ \boldsymbol{A} $. Rather, we define sensitivity parameters to model our uncertainty about the missing alter–ego and ego–ego edges. Therefore, all subsequent probability statements regarding missing edges should be interpreted strictly as quantifications of this uncertainty.

By the exposure mapping specification (Assumption 2) and the experimental design (Assumption 1), the relevant edges in the population network $ \boldsymbol{A} $ that determine the probabilities $ \pi_{i}^{a} $ and $ \pi_{i}^{e} $ are only the alter–ego or ego–ego edges. In the observed network $ \widetilde{\boldsymbol{A}} $, each alter $ i\in\mathcal{R}_{a} $ is connected only to its ego $ e(i)\in\mathcal{R}_{e} $. Let $ \mathcal{R}_{e}^{j}=\mathcal{R}_{e}\setminus\{j\} $ be the set of all egos other than $ j\in\mathcal{R}_{e} $. The latent alter–ego edges $ \left\{A_{ij}:i\in\mathcal{R}_{a},j\in\mathcal{R}_{e}^{e(i)}\right\} $, and ego–ego edges $ \left\{A_{ij}:i, j\in\mathcal{R}_{e},i\neq j\right\} $ are the edges missing from $ \widetilde{\boldsymbol{A}} $, relevant to the exposure probabilities. Let $ \rho^{e}_{ij} $ and $ \rho^{a}_{ij} $ represent the postulated probabilities that a missing edge exists, ie


(12) \begin{align*}\rho^{e}_{ij}&=\Pr\left(A_{ij}\,=\,1\mid i, j\in\mathcal{R}_{e},i\neq j\right),\nonumber\\\rho^{a}_{ij}&=\Pr\big(A_{ij}\,=\,1\mid i\in\mathcal{R}_{a},j\in\mathcal{R}_{e}^{e(i)}\big).\end{align*}

Using (12), we can derive expressions for $ \pi^{e}_{i} $ and $ \pi^{a}_{i} $. By the law of total probability and Assumption 1, $ \Pr\left(Z_{j}A_{ij}\,=\,1\mid i\neq j\in\mathcal{R}_{e}\right)=p_{z}\rho^{e}_{ij} $. Assuming edge independence yields


(13) \begin{align*}\pi^{e}_{i}\,=\,1-\prod\limits_{j\in\mathcal{R}_{e}^{i}}\big(1-p_{z}\rho^{e}_{ij}\big).\end{align*}

Turning to $ \pi^{a}_{i} $, alter $ i\in\mathcal{R}_{a} $ is exposed ($ F_{i}\,=\,1 $) only if its ego * e(i) * or at least 1 of the other recruited egos connected to * i * in the population network $ \boldsymbol{A} $ is treated. Therefore, we obtain (Supplement A.2)


(14) \begin{align*}\pi^{a}_{i}=p_{z}\,+\,(1-p_{z})\Bigg[1-\prod\limits_{j\in\mathcal{R}_{e}^{e(i)}}\big(1-p_{z}\rho^{a}_{ij}\big)\Bigg].\end{align*}

Equations (13) and (14) illustrate how the exposure probabilities can be computed given specifications of the edge-level sensitivity parameters $ \rho^{e}_{ij} $ and $ \rho^{a}_{ij} $.

We now discuss some prominent examples of how researchers can specify these parameters in practice. The examples include both homogeneous and heterogeneous edge probabilities, as well as specifications based on the expected number of missing edges.

Example 1 **(Homogeneous probabilities).** If researchers are willing to specify the same probabilities (12) for all units, i.e., $ \rho^{e}_{ij}=\rho^{e} $ and $ \rho^{a}_{ij}=\rho^{a} $, then the exposure probabilities (13) and (14) simplify to

\begin{align*}\pi_{i}^{e}&=1-(1-p_{z}\rho^{e})^{n_{e} -1},\\ \pi_{i}^{a}&=p_{z}\,+\,(1-p_{z})\left[1-(1-p_{z}\rho^{a})^{n_{e}-1}\right].\end{align*}

For instance, if researchers suspect that about * 1% * of the ego–ego edges are possible, they can set $ \rho^{e}\,=\,0.01 $.

Example 2 **(Homogeneous number of edges).** A special case of Example 1 occurs by specifying the approximate *expected number* of latent edges, separately for the ego–ego and alter–ego edges. Let $ m^{e}\in\left\{1 , \ldots , \binom{n_{e}}{2}\right\} $ be the total number of ego–ego edges that are believed to be missing. Setting

\begin{align*}\rho^{e}_{ij}=\rho^{e}(m^{e})=\frac{m^{e}}{\binom{n_{e}}{2}},\end{align*}

weights equally each ego–ego edge probability and yields $ m^{e} $ expected ego–ego edges.

There are $ n_{a}(n_{e}-1) $ possible missing alter–ego edges. If approximately $ m^{a} $ such edges are expected to be missing, set the probability of each edge to


\begin{align*}\rho^{a}_{ij}=\rho^{a}(m^{a})=\frac{m^{a}}{n_{a}(n_{e}-1)}.\end{align*}

For instance, if researchers suspect that each alter is connected to approximately $ c^{a}\geq 0 $ additional egos, they can select $ m^{a}=c^{a}\times n_{a} $. Similarly, if each ego is believed to be connected to approximately $ c^{e}\geq 0 $ additional egos, then $ m^{e}=\frac{c^{e}\times n_{e}}{2} $.

Example 3 **(Heterogeneous probabilities).**  Example 1 can be extended by allowing edge probabilities to vary based on unit-level covariates $ \boldsymbol{X}_{i} $. This captures the well-known phenomenon in network science of homophily (McPherson et al. 2001), where units with similar characteristics are more likely to interact.

We begin with a baseline probability parameter, $ \rho^{e}_{base} $, analogous to the homogeneous parameter $ \rho^{e} $ in Example 1. We then adjust this baseline using a pairwise dissimilarity measure $ d_{ij}=d(\boldsymbol{X}_{i},\boldsymbol{X}_{j}) $. Common choices include the $ L^{p} $ norm, $ d_{ij}=||\boldsymbol{X}_{i}-\boldsymbol{X}_{j}||_{p} $, or the Cosine distance, $ d_{ij}\,=\,1-\frac{\boldsymbol{X}_{i}^{T}\boldsymbol{X}_{j}}{||\boldsymbol{X}_ {i}||||\boldsymbol{X}_{j}||} $. To incorporate these distances while preserving symmetry ($ \rho^{e}_{ij}=\rho^{e}_{ji} $), we represent the edge probabilities using the logistic link function:


\begin{align*}\mathrm{logit}(\rho^{e}_{ij})=\mathrm{logit}(\rho^{e}_{base})-\gamma^{e}\left(d_{ij}-\overline{d}^{e}\right),\end{align*}

where $ \overline{d}^{e}=\frac{2}{n_{e}(n_{e}-1)}\sum_{i\,\lt\,j\in\mathcal{R}_{e}}d_{ij} $ is the average distance between all ego pairs. Here, $ \gamma^{e}\geq 0 $ is a sensitivity parameter controlling the *strength of homophily*. By centering the distances (subtracting $ \overline{d}^{e} $), we decouple the network density from the homophily structure such that $ \rho^{e}_{base} $ controls the overall network density and $ \gamma^{e} $ controls how strongly edges concentrate among similar units. Setting $ \gamma^{e}\,=\,0 $ eliminates the distance term, recovering the homogeneous probabilities of Example 1 ($ \rho^{e}_{ij}=\rho^{e}_{base} $). Larger values of $ \gamma^{e} $ decrease the probability of edges between dissimilar units (where $ d_{ij} \gt\overline{d}^{e} $) and increase it for similar units.

Analogous definitions apply for alter–ego edges using a baseline $ \rho^{a}_{base} $, a sensitivity parameter $ \gamma^{a} $, and centered distances between alters and potential egos.

Example 4 **(Heterogeneous number of missing edges).**  Examples 2 and 3 can be combined to allow pair-specific heterogeneous probabilities while fixing the expected number of missing edges. Let $ w_{ij}^{e}=\exp(-\gamma^{e}d_{ij}^{e}) $ and $ w_{ij}^{a}=\exp(-\gamma^{a}d_{ij}^{a}) $ be the exponential of the negative measures defined in Example 3, for fixed values of $ \gamma^{e} $ and $ \gamma^{a} $. Larger values of $ w_{ij}^{e} $ and $ w_{ij}^{a} $ indicate higher similarity between the covariates of * i * and * j *. Define the total sum of weights among all distinct ego–ego and alter–ego pairs with missing edges:

\begin{align*} W^{e}=\sum\limits_{i, j\in\mathcal{R}_{e},i\,\lt\,j}w_{ij}^{e},\qquad W^{a}=\sum\limits_{i\in\mathcal{R}_{a}}\sum\limits_{j\in\mathcal{R}_{e}^{e(i)}}w_{ij}^{a}.\end{align*}

Similar to Example 2, researchers can specify $ m^{e} $ and $ m^{a} $, the approximate number of missing ego–ego and alter–ego edges, respectively, and set:


\begin{align*}\rho^{e}_{ij}=\frac{m^{e}}{W^{e}}\, w^{e}_{ij},\qquad\rho^{a}_{ij}=\frac{m^{a}} {W^{a}}\, w^{a}_{ij}.\end{align*}

This specification reflects that the expected number of missing edges is approximately $ m^{e} $ and $ m^{a} $, while allowing for heterogeneous edge probabilities. Setting all weights ($ w_{ij}^{e} $ and $ w_{ij}^{a} $) to 1 recovers Example 2.

### 3.5. Running the sensitivity analysis in practice

To assess the impact of contamination on the causal estimates, researchers can utilize the bias-corrected estimators for the indirect effect and for the direct effect. The bias-corrected estimators depend on the sensitivity parameters that define the edge-level probabilities (12) and the parameter * κ * for the direct effect. Examples 1 to 4 provide practical guidelines on how to specify these parameters in practice. When the true values of such parameters are unknown and cannot be estimated from the data, researchers can resort to sensitivity analysis by exploring a range of plausible values. We consider here two approaches: GSA and PBA.

In GSA, researchers specify a grid of values for the sensitivity parameters and compute the bias-corrected estimates for each combination of parameters (Rosenbaum and Rubin 1983; Greenland 1996). That results in a grid of bias-corrected estimates. Such estimates can be coupled with the associated CI given each value of the sensitivity parameters in the specified grid. Researchers can then summarize the sensitivity analysis results by reporting the answers for questions such as “what is the largest number of missing alter-ego edges such that the CI for * IE * excludes the value zero?” or “are there combinations of $ \rho^{e}_{ij} $ and * κ * such that $ \widehat{DE}_{adj} $ flips its sign compared to the naive estimator?”

In PBA, researchers specify a distribution for the sensitivity parameters and compute the distribution of bias-corrected estimates over this distribution (Greenland 2005; Lash et al. 2014; Fox et al. 2021). In practice, this distribution is approximated using Monte Carlo by sampling from the specified distribution and summarized through averages and intervals of the resulting bias-corrected estimates. This distribution of bias-corrected estimates accounts for uncertainty in the sensitivity parameters (bias uncertainty). Furthermore, as described below, it is possible to compute a distribution of estimates that accounts for both statistical uncertainty and bias uncertainty. In our design-based framework, statistical uncertainty arises solely from the treatment assignment distribution. A review of PBA is given in Supplement C.

We now describe the GSA and PBA approaches in more detail.

#### 3.5.1. Grid sensitivity analysis

Given a grid of sensitivity parameters, the full procedure is as follows:

1. (Optional.) Get the predicted values for the outcome models $ \widehat{\mu}^{a}_{i}(f) $ and $ \widehat{\mu}^{e}_{i}(z) $ using the cross-fitting procedure described in Section 3.3. These predicted values are calculated only once and used for all sensitivity parameter values.
2. For each parameter value in the grid of sensitivity parameters:
  a. Compute bias-corrected estimates $ \widehat{IE}_{adj} $ or $ \widehat{IE}^{aug}_{adj} $ and $ \widehat{DE}_{adj} $ or $ \widehat{DE}^{aug}_{adj} $.
  b. Estimate the variance of each bias-corrected estimator.
  c. Compute CIs for each estimator.

The results are then summarized by presenting the estimators and associated CIs as a function of the sensitivity parameter(s), as we demonstrate in the data analysis (Section 5). Details on estimating variance and CIs are provided in Sections 3.1 to 3.3.

#### 3.5.2. Probabilistic bias analysis

Instead of specifying a grid of sensitivity parameters, researchers assign a distribution for each parameter. For instance, under the setting in Example 1, if researchers believe that approximately 1% of the possible ego–ego edges are missing from $ \widetilde{\boldsymbol{A}} $, they can set $ \rho^{e}\sim\mathrm{Beta}(a, b) $ and choose the values of * a * and * b * such that the mean is $ \frac{a}{a\,+\,b}\,=\,0.01 $, and the variance reflects the level of uncertainty about $ \rho^{e} $. Let * p(·) * be the specified distribution for the sensitivity parameters. In practice, researchers can specify a separate distribution for each parameter.

Let $ B\,\gt\,0 $ be the number of Monte Carlo samples. The PBA procedure that accounts for both bias and design uncertainty proceeds as follows.

1. For each $ b\,=\,1 , \ldots, B $:
  a. Sample sensitivity parameters from the distribution * p(·) *.
  b. Compute the bias-corrected estimates $ \widehat{IE}_{adj}^{(b)} $ and $ \widehat{DE}_{adj}^{(b)} $ under the sampled values.
  c. Draw from the (approximate) distribution of the estimator
    \begin{align*}\widehat{IE}_{adj}^{(b, rand)} \sim N\left(\widehat{IE}_{adj}^{(b)},\widehat{V}_ {\boldsymbol{Z}}\left(\widehat{IE}_{adj}\right)\right),\end{align*}
    and similarly for the other estimators.
2. Summarize the distribution of bias-corrected estimates. For example, by computing the average $ \frac{1}{B}\sum_{b\,=\,1}^{B}\widehat{IE}_{adj}^{(b, rand)} $ and empirical percentiles for intervals.

If researchers use the augmented estimators $ \widehat{IE}_{adj}^{aug} $ and $ \widehat{DE}_{adj}^{aug} $ (Section 3.3), the outcome models can be precomputed as in GSA step 1. Step 1c propagates the statistical uncertainty (w.r.t. $ \boldsymbol{Z} $) into the estimators by simulating a draw from the estimators’ asymptotic normal distribution. Therefore, the distribution of estimates $ \widehat{IE}_{adj}^{(b, rand)} $ accounts for both bias uncertainty about the sensitivity parameters and statistical uncertainty resulting from the treatment assignments (Greenland 2005; Fox et al. 2021). Further technical details and motivation of the PBA procedure are provided in Supplement C.

### 3.6. Estimation of sensitivity parameters with internal validation data

Until this section, the exposure probabilities $ \pi_{i}^{a} $ and $ \pi_{i}^{e} $ (or the basic components of latent edge probabilities $ \rho_{ij}^{a} $ and $ \rho_{ij}^{e} $) and * κ * have been treated as unknown sensitivity parameters. However, these parameters can be directly estimated with different types of internal validation data, thus alleviating the reliance on pure sensitivity analysis. We now describe the specific data types required for estimating these parameters, separately for alters (network members) and egos.

#### 3.6.1. Internal validation data for alters

Recall that by ENRT design and the exposure mapping specification (Assumption 2), alters’ exposure misclassification is one-sided. That is, alters with observed exposure $ \widetilde{F}_{i}\,=\,1 $ will have a correctly classified exposure, while those with $ \widetilde{F}_{i}\,=\,0 $ can have a true exposure of $ F_{i}\,=\,0 $ or $ F_{i}\,=\,1 $. Therefore, in the study follow-up period, researchers can use a validation subset of alters from the nontreated ego-networks and ask them to recall information about the intervention. For example, in the HPTN 037 study, the treated egos underwent training sessions that included a number of specific phrases and terms designed to assess the diffusion of intervention messages. Thus, alters connected to a nontreated ego should have no recollection of these terms. In the follow-up period, alters within a validation subset were asked to recall terms associated with the intervention to assess possible contamination (Simmons et al. 2015; Aroke et al. 2023; Chao et al. 2025). This information can be used to estimate the conditional probability $ \Pr(F_{i}\,=\,1\mid\widetilde{F}_{i}\,=\,0, i\in\mathcal{R}_{a}) $ and subsequently the exposure probabilities $ \pi_{i}^{a} $ or, in the case of homogeneous probabilities (Examples 1 and 2), the edge probabilities $ \rho^{a} $. We describe this in more detail in Supplement A.11. In Section 5, we compare the bias-adjusted indirect effect estimates resulting from the calibration using the internal validation data of the study to the sensitivity analysis described in the preceding subsections.

#### 3.6.2. Internal validation data for egos

Similar recall data described for the alters can be used to assess the exposure probabilities of egos, in the case of homogeneous exposure probabilities. Nontreated egos should have no recollection of the terms described in the intervention. Therefore, nontreated egos that recall intervention-related information in the follow-up period ostensibly obtained this information due to contamination from other egos. Under homogeneous exposure probabilities, both $ \pi^{e} $ and $ \rho^{e} $ can be estimated using the proportion of non-treated egos in the follow-up period that recalled intervention-related information (Supplement A.11).

Alternatively, researchers can ask the egos more direct and informed questions. For example, instead of recalling *any* terms associated with the intervention, researchers can ask the egos if they learned about the intervention from other friends or sources. That will provide information regarding the true exposure status of egos in the validation subsample. These internal validation data can be used to estimate both $ \pi_{i}^{e} $ and * κ * (Supplement A.11). This type of information was not collected in the HPTN 037 study and can be of valuable importance for future studies.

## 4. SIMULATION STUDY

We conducted a simulation study to evaluate the performance of the bias-corrected estimators $ \widehat{IE}_{adj},\widehat{IE}_{adj}^{aug},\widehat{DE}_{adj}, $ and $ \widehat{DE}_{adj}^{aug} $. We assessed the bias, variance estimation, and empirical coverage of the CIs under the true values of the sensitivity parameters. Furthermore, we evaluated how misspecification of * κ * values or violation of Assumption 4 affects the estimation of the direct effect.

The simulation study is based on a data-generating process that partly mimics the settings of the HPTN 037 study (Latkin et al. 2009). We generated $ n_{e}\,=\,200 $ disjoint ego-networks with two alters each, which was the average number of alters per ego-network in HPTN 037, resulting in $ n_{a}\,=\,400 $. Contamination between the ego-networks was generated by specifying the expected number of missing alter–ego and ego–ego edges, as in Examples 2 and 4, and drawing the latent edges independently. Specifically, we set the expected number of missing alter–ego edges to $ m^{a}\in\{100,200\} $, and ego–ego edges to $ m^{e}\in\{150,250\} $. Under each setup, a single network was generated and was fixed throughout the simulation replications. The potential outcomes were generated once according to the models


\begin{align*} Y_{i}(z, f)&=-0.5\,+\,2z\,+\,0.5f\,+\,zf+\boldsymbol{X}_{i}^{T}\boldsymbol{\beta}_{e}+\varepsilon_{i},&\mathrm{for}\; i\in\mathcal{R}_{e},\\Y_{i}(0, f)&=-0.5\,+\,2f+\boldsymbol{X}_{i}^{T}\boldsymbol{\beta}_{a}+\varepsilon_{i},&\mathrm{for}\; i\in\mathcal{R}_{a},\end{align*}

where $ \varepsilon_{i}\sim N(0,1) $ are independent noise terms. This data-generating process yields true effects of $ DE\,=\,IE = 2 $, and ratio of direct effects among exposed versus non-exposed egos of * κ = 1.5 * as defined in (7). The generation process of the three covariates $ \boldsymbol{X}_{i} $ and the specification of $ \boldsymbol{\beta}_{e} $ and $ \boldsymbol{\beta}_{a} $ are provided in Supplement D. Treatments were assigned according to a Bernoulli design with $ p_{z}\,=\,0.5 $. We performed $ 5\times 10^{3} $ simulation replications by random draws of treatment assignments.

We report here the results when contamination was generated using heterogeneous edge probabilities as in Example 4 with Euclidean distance and $ \gamma^{e}=\gamma^{a}\,=\,1 $. We compare the bias-corrected estimators under the specification that uses the correct edge probabilities (“Heterogeneous”), under the specification that uses a misspecified model with the same edge probabilities as in Example 2 (“Homogeneous”) and the uncorrected estimators $ \widehat{IE} $ and $ \widehat{DE} $ (“Naive”). We considered both the HT estimators and the augmented estimators using an outcome model estimated via linear regression with two-fold cross-fitting (as described in Section 3.3).

**Table 1:** Simulation results for indirect and direct effects under heterogeneous contamination. Bias is the empirical bias, Coverage is the empirical coverage of the 95% CIs, and SD/SE is the ratio of the SD of the estimates to the mean SE estimates. Specification “Heterogeneous” uses the correct edge probabilities, “Homogeneous” uses the same probability for all edges, and “Naive” is the uncorrected estimator. Augmented indicates whether the estimators augmented with an outcome model are used. True effects are * IE = 2 * and * DE = 2 *.

Estimand	Scenario	Specification	Augmented	Bias	Coverage	SD/SE
			FALSE	−0.006	0.963	0.948
		Heterogeneous	TRUE	−0.007	0.948	0.988
			FALSE	−0.004	0.963	0.949
		Homogeneous	TRUE	−0.005	0.949	0.987
* IE *	$ m^{a}\,=\,100 $		FALSE	−0.239	0.507	0.949
		Naive	TRUE	−0.240	0.403	0.987
			FALSE	0.002	0.970	0.902
		Heterogeneous	TRUE	0.000	0.955	0.988
			FALSE	0.011	0.971	0.904
		Homogeneous	TRUE	0.009	0.954	0.987
	$ m^{a}\,=\,200 $		FALSE	−0.434	0.074	0.904
		Naive	TRUE	−0.435	0.026	0.987
			FALSE	0.000	0.986	0.780
		Heterogeneous	TRUE	−0.005	0.960	0.952
			FALSE	−0.011	0.984	0.802
		Homogeneous	TRUE	−0.016	0.956	0.960
* DE *	$ m^{e}\,=\,150 , \kappa = 1.5 $		FALSE	0.514	0.279	1.068
		Naive	TRUE	0.508	0.175	1.045
			FALSE	0.000	0.999	0.574
		Heterogeneous	TRUE	−0.005	0.981	0.829
			FALSE	−0.019	0.998	0.596
		Homogeneous	TRUE	−0.024	0.976	0.840
	$ m^{e}\,=\,250 , \kappa = 1.5 $		FALSE	0.689	0.102	1.064
		Naive	TRUE	0.682	0.023	1.029

Table 1 summarizes the results for the indirect and direct effect estimation. As the level of contamination ($ m^{a} $ and $ m^{e} $) increases, the naive estimators exhibit substantial bias and poor coverage. In contrast, all bias-corrected estimators exhibit negligible bias across all scenarios, illustrating that even a misspecified model for the edge probabilities (homogeneous specification) can effectively correct for the bias. For the non-augmented estimators, the variance estimates were conservative, particularly for the direct effect in high contamination settings (SD/SE * =0.574 *). This is comparable to the conservativeness of the variance estimators in other papers in the network interference literature (e.g., Aronow and Samii 2017; Weinstein and Nevo 2026). The augmented indirect effect estimators, on the other hand, yielded valid variance estimates and achieved nominal coverage rates. The augmented direct effect estimators tend to slightly overestimate the variance (SD/SE * < 1 *), which is attributed to their conservative variance estimator (Section 3.2). Results for homogeneous contamination settings are provided in Supplement D and exhibit similar patterns.

We also evaluated the impacts of misspecifying the value of * κ * or violating Assumption 4. We found that violations of Assumption 4, induced by heterogeneous treatment-exposure interactions, resulted in negligible bias for the corrected direct effect estimators, albeit with slight variance underestimation. The impact of * κ * misspecification depended on the direction of the misspecification compared to the true value. If * κ * is misspecified in the wrong direction (e.g., specifying * κ < 1 * when * κ > 1 *), the bias-corrected estimator can exhibit severe bias and lower coverage than the naive estimator. Conversely, when * κ * is misspecified in magnitude but in the correct direction, the bias-corrected estimator outperforms the naive estimator. Moreover, the performance of the proposed bias-corrected estimators under binary potential outcomes was also assessed, yielding results consistent with those under the continuous potential outcome model. See Supplement D for additional details and results.

## 5. DATA ANALYSIS

HPTN 037 was an ENRT that evaluated the efficacy of a peer education intervention in reducing HIV risk behaviors among people who inject drugs and their drug and sexual networks (Latkin et al. 2009). Egos were randomized ($ p_{z}\,=\,0.5 $) to receive a peer education training and were encouraged to share the HIV risk reduction information with their drug and sexual network members (alters). Egos in the control arm received standard HIV prevention counseling. The study was conducted between 2002 and 2006 in Philadelphia, Pennsylvania, US and Chiang Mai, Thailand. The study recruited 414 egos (index participants) who were asked to recruit at least one drug or sex network member (alter) into the study. Researchers enforced that alters were not also egos, and that each alter was connected to only one ego in the observed network (Latkin et al. 2009). Following previous analyses (Buchanan et al. 2018, 2024; Chao et al. 2025), we limit our analysis to the Pennsylvania site and to a 12-month follow-up, yielding a sample of $ n_{e}\,=\,150 $ egos and $ n_{a}\,=\,263 $ alters, with an average of 2 alters per ego-network. The primary outcome is a binary indicator of any injection-related risk behavior in the past month, including sharing injection equipment, front and back loading, and injecting with people not well known or in a public space.

Contamination across ego-networks is a concern in ENRT. That is, alters might inject drugs or have sexual relations with egos from other ego-networks, or egos might be connected to each other. Using recall data collected after the trial, Simmons et al. (2015) found evidence of contamination. Chao et al. (2025) developed a bias-correction method to adjust for (homogeneous) exposure misclassification, only for the indirect effect, building on the recall data and the assumption of non-overlapping ego-networks. That is, as in most ENRT studies, the analysis in Chao et al. (2025) assumed that, in the population network, alters can only be connected to one ego, and egos can not be connected to each other.

We relax this assumption and apply our proposed sensitivity analysis methods to assess the potential impact of contamination on both indirect and direct effect estimates. For both alter–ego and ego–ego edges, we specify the expected number of missing edges as in Examples 2 and 4. Covariates used in the analysis include risk behaviors at baseline, age, gender, and race. In the outcome models, we also included the average neighbors’ covariates for each unit, as was also done by Buchanan et al. (2024). Quantitative covariates were scaled to have a mean of 0 and a variance of 1. For the specification of heterogeneous edge probabilities using the approximate expected number of missing edges (Example 4), we use the Euclidean distance and set $ \gamma^{e}=\gamma^{a}\,=\,1 $. Results with $ \gamma^{e}=\gamma^{a}\,=\,2 $ are given in Supplement E and were consistent with the results shown here, albeit with higher variance estimates. Note that here $ \gamma^{e} $ and $ \gamma^{a} $ are hyperparameters and not the sensitivity parameters, which are * κ *, $ m^{a} $, and $ m^{e} $. For the direct effect, we specify values larger than 1 to the sensitivity parameter * κ *, addressing the concern that the direct effect is larger, on average, among egos exposed to at least one other treated ego. We apply both the GSA and PBA described in Section 3.5. Variance estimates and 95% CIs were calculated as described in Sections 3.1 to 3.3.

Furthermore, we compare the results of the sensitivity analysis with those obtained using point estimates of parameters from internal validation data, as described in Section 3.6. During the 6-month follow-up of the study, participants were asked to recall terms introduced in the intervention training program and unlikely to be known outside of it. Following previous analyses (Aroke et al. 2023; Chao et al. 2025), we assume that a participant was indirectly exposed to the intervention if she recalled at least one of the five terms associated with the intervention. For greater accuracy, we limit the validation subset only to participants who recalled the positive term and none of the negative terms (Aroke et al. 2023; Chao et al. 2025). As described in Section 3.6, we estimate $ \pi^{a} $ from nonexposed alters ($ \widetilde{F}_{i}\,=\,0, i\in\mathcal{R}_{a} $) and $ \pi^{e} $ from nontreated egos ($ Z_{i}\,=\,0, i\in\mathcal{R}_{e} $), both under homogeneous exposure probabilities. Among 22 alters and 15 egos in the validation subset, only 4 alters and 3 egos recalled the terms, mapping to an estimated exposure probabilities of $ \widehat{\pi}^{a}\,=\,0.59 $ and $ \widehat{\pi}^{e}\,=\,0.2 $. From Example 2 this is equivalent to approximately $ \widehat{m}^{a}\,=\,105.5 $ missing ego-alter edges and $ \widehat{m}^{e}\,=\,33.4 $ missing ego-ego edges.

**Figure 2:**
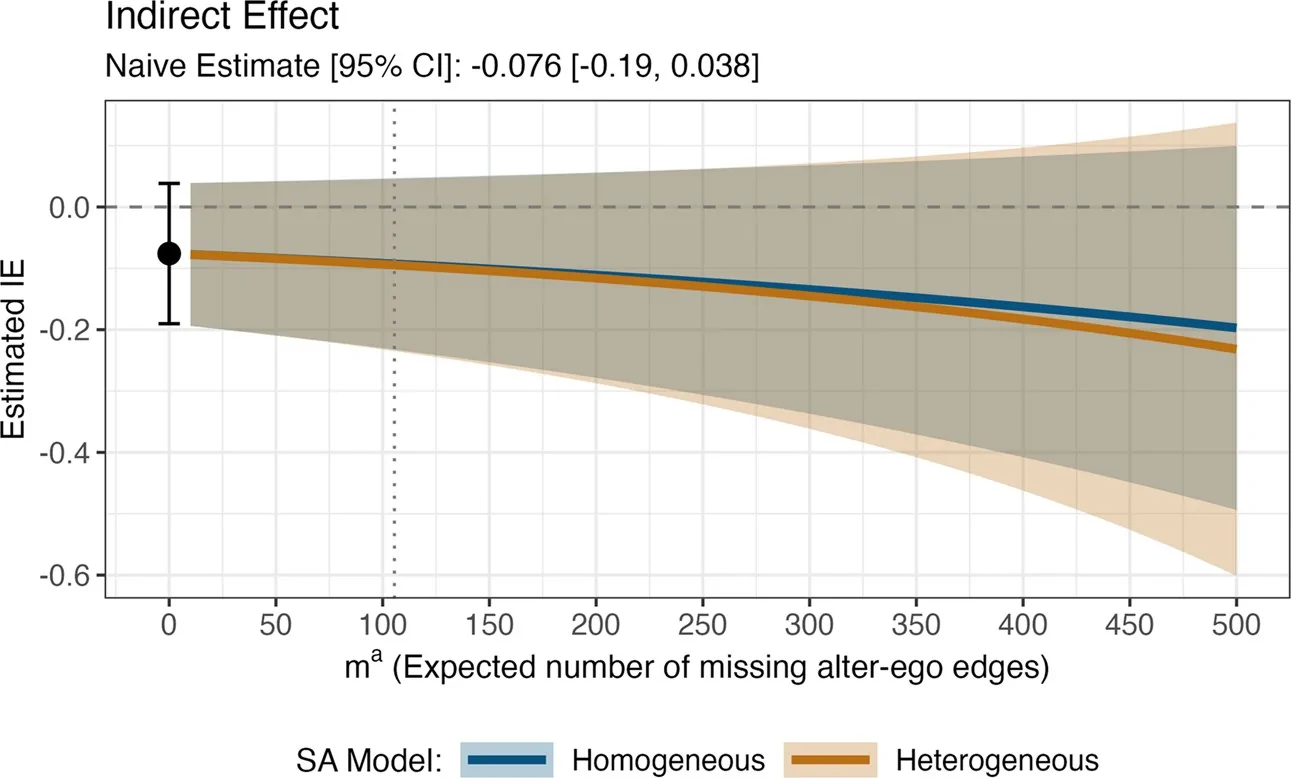
GSA results for indirect effect using the augmented estimator $ \widehat{IE}_{adj}^{aug} $. The *x*-axis represents the approximate total number of missing alter–ego edges $ m^{a} $, while the *y*-axis represents the estimates. The dashed vertical line at 105.5 is the estimate of $ m^{a} $ from the internal validation data. Results are shown for both homogeneous (blue) and heterogeneous (orange) edge probabilities specifications.

**Figure 3:**
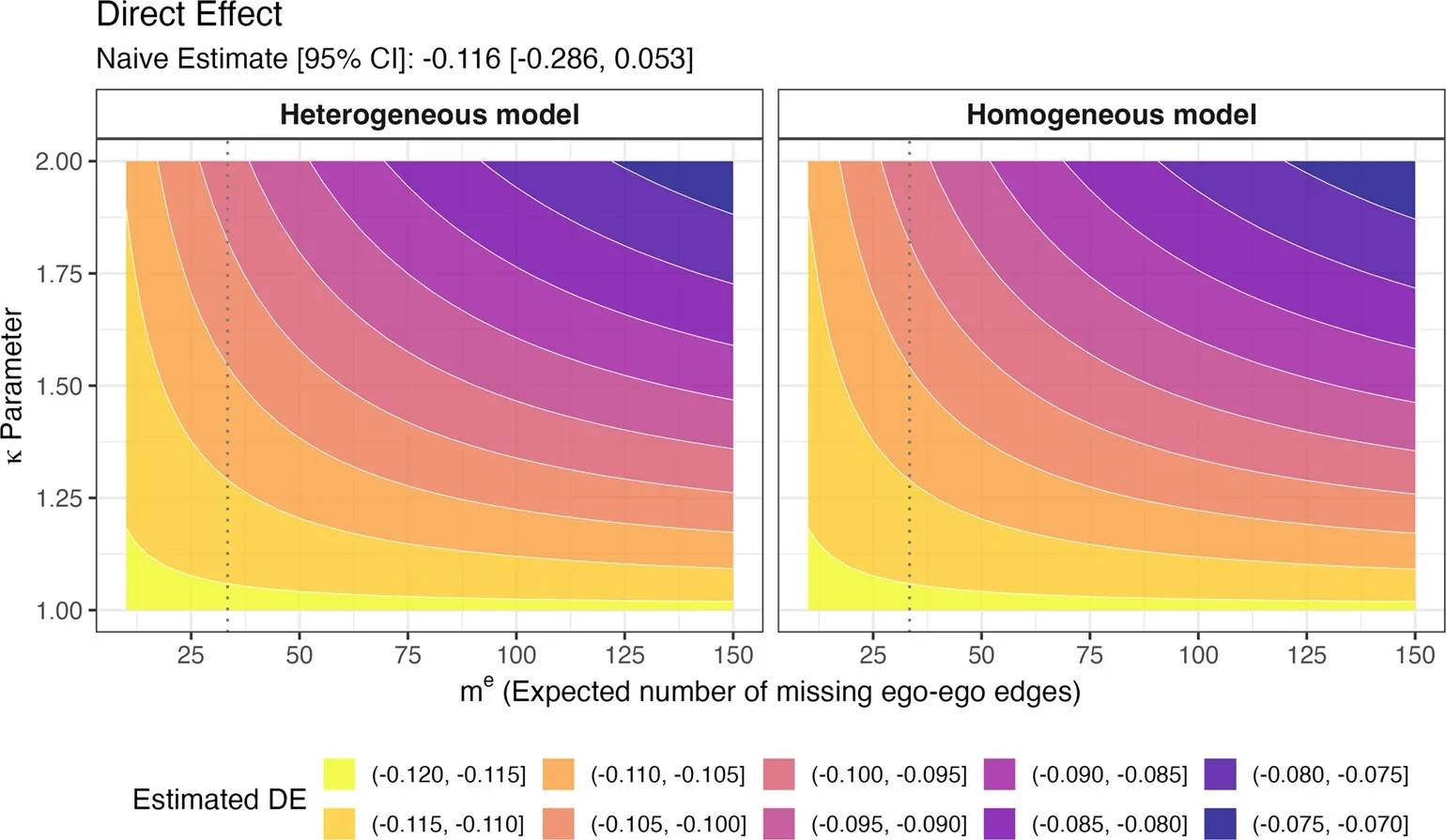
GSA results for direct effect using the augmented estimator $ \widehat{DE}_{adj}^{aug} $. The *x*-axis represents the approximate total number of missing ego–ego edges $ m^{e} $, while the *y*-axis represents * κ *, the interaction sensitivity parameter. The dashed vertical line at 33.4 is the estimate of $ m^{e} $ from the internal validation data. Contour lines and color shading represent the estimated values. Results are shown for both heterogeneous (left) and homogeneous (right) edge probability specifications.

### 5.1. Grid sensitivity analysis

For the indirect effect, we consider a grid of the approximate expected number of missing edges with $ m^{a}\in[10,500] $ and $ m^{e}\in[10,150] $, representing the concern that, in the homogeneous case, each alter and ego are approximately connected to at most two additional egos (Example 2). In addition, we consider $ \kappa\in[1,2] $ reflecting the suspicion that the direct effect among egos connected to at least one treated ego is up to two times larger, on average, than the effect among egos that are not connected to other treated egos. We report here the results for the augmented estimators $ \widehat{IE}_{adj}^{aug} $ and $ \widehat{DE}_{adj}^{aug} $ using two-fold cross-fitting (Section 3.3) and logistic regression for the outcome models. We consider both homogeneous and heterogeneous edge probability specifications. Results for the non-augmented estimators $ \widehat{IE}_{adj} $ and $ \widehat{DE}_{adj} $ are provided in Supplement E, and were consistent with the results shown here.

**Figure 4:**
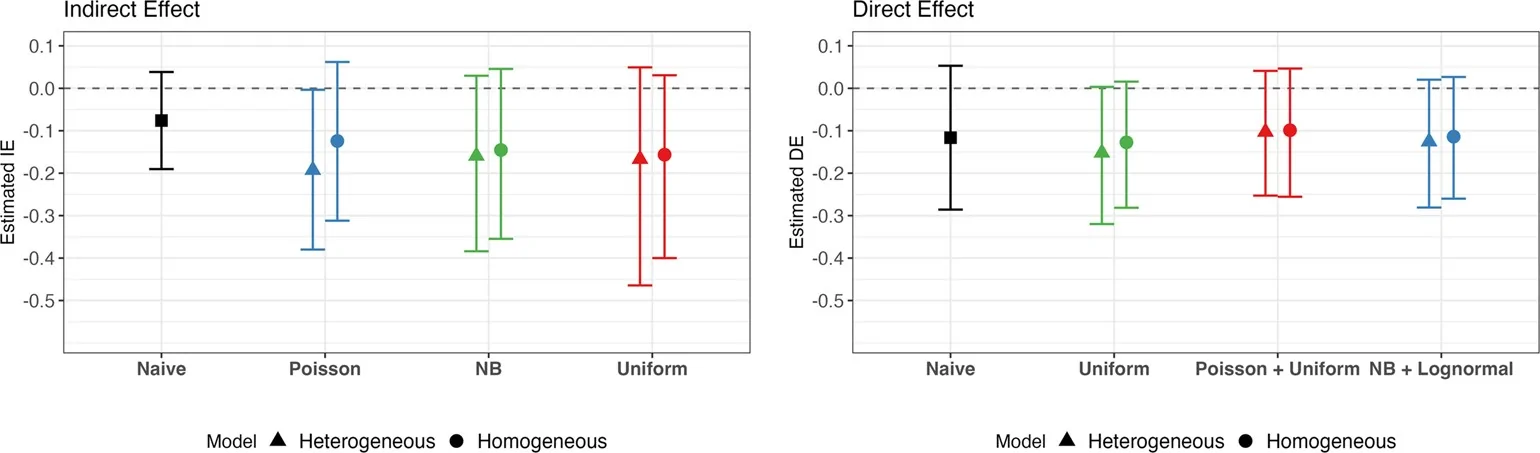
PBA results for indirect effect (left) using the augmented estimator $ \widehat{IE}_{adj}^{aug} $, and direct effect (right) using the augmented estimator $ \widehat{DE}_{adj}^{aug} $. Results are shown as mean and * 95% * intervals for both homogeneous and heterogeneous edge probability specifications, accounting for both bias and statistical uncertainty. Colors represent different specified distributions for the total number of missing edges and the sensitivity parameter * κ *.

Figures 2 and 3 summarize the GSA results for indirect and direct effects, respectively. For the indirect effect, both homogeneous and heterogeneous specifications yield similar results. The results suggest that, for instance, if each alter is connected to up to 1 additional ego on average ($ m^{a}\,=\,263 $) in the homogeneous case, the bias-corrected estimate is around * -0.194 * compared to the naive estimate of * -0.076 *, and a bias-corrected estimate of around * -0.093 * using the estimate $ \widehat{m}^{a}\,=\,105.5 $ from the internal validation data. Taking the estimate $ \widehat{m}^{a}\,=\,105.5 $ as a lower bound for the true $ m^{a} $, implies that the naive estimator underestimate * IE * by an order of at least * 18% *, indicating a substantially larger indirect effect than previously reported.

For the direct effect, the bias-corrected estimates under the homogeneous and heterogeneous specifications yielded similar estimates that are closer to 0 than the naive estimate of * -0.116 * across the entire grid of sensitivity parameters. For example, if * κ = 1.5 * and each ego is connected to up to 1 additional ego on average ($ m^{e}\,=\,75 $), the bias-corrected estimate is around * -0.097 * in the homogeneous case, compared to the bias-corrected estimate of around * -0.105 * using the estimate $ \widehat{m}^{e}\,=\,33.4 $ from the internal validation data. These results imply that the naive estimates of * DE * might be overestimated by over * 10.5% *, indicating a smaller direct effect than previously found.

### 5.2. Probabilistic bias analysis

For the PBA, we specify distributions for the sensitivity parameters, representing different degrees of uncertainty about their values. For $ m^{a} $ and $ m^{e} $, we compare three uncertainty distributions. The first is a discrete uniform distribution over the ranges used in the GSA: $ m^{a}\sim\mathrm{Uniform}\{1,500\} $ and $ m^{e}\sim\mathrm{Uniform}\{1,150\} $, representing maximum entropy, but with a capped right tail. The second is a Poisson distribution with a mean equal to half of the maximum value in the GSA grid: $ m^{a}\sim\mathrm{Poisson}(250) $ and $ m^{e}\sim\mathrm{Poisson}(75) $, representing moderate uncertainty with a thin but unbounded tail. The third is a Negative Binomial distribution with a mean equal to half the maximum value in the GSA grid and a size parameter equal to 10, representing a heavier tail than the Poisson distribution. For the sensitivity parameter * κ *, we consider two uncertainty distributions. The first is a continuous uniform distribution, $ \kappa\sim\mathrm{Uniform}[1,2] $, and the second is a log-normal distribution with expected value 1.5 and SD equal to 0.2 on the log scale, resulting in a heavier right tail than the uniform distribution. We run the PBA procedure with $ B\,=\,10^{4} $ Monte Carlo samples and account for both bias and statistical uncertainty, as described in Section 3.5.

Figure 4 summarizes the PBA results for the indirect and direct effects with the augmented estimators. For the indirect effect, the three distributions for $ m^{a} $ yield similar average estimates, with a slightly larger magnitude for the heterogeneous specification. The uniform distribution results in the widest intervals, while the Poisson distribution yields the narrowest intervals. All bias-corrected estimates are lower (average estimates around * -0.158 *) than the naive estimate of * -0.076 *, suggesting that contamination leads to severe underestimation of the magnitude of the indirect effect. For the direct effect, all specified distributions for $ m^{e} $ and * κ * yield similar average estimates, with the Poisson and uniform combination resulting in estimates closer to zero than the rest. The uniform distribution yields the widest intervals. The averages of the bias-corrected estimates are closer to zero (average estimates around * -0.12 * in the heterogeneous specification) than the naive estimate of * -0.116 *, suggesting that contamination leads to slight overestimation of the direct effect. Nevertheless, apart from the heterogeneous specification with the Poisson distribution for indirect effect, the indirect and direct * 95% * PBA intervals include 0 in all distribution and edge probabilities specifications.

## 6. DISCUSSION

In this paper, we developed sensitivity analysis methods to assess the impact of contamination between ego-networks on the causal estimates in ENRTs. Such trials are increasingly used to evaluate interventions in hard-to-reach populations, where full network information is often unavailable or difficult to obtain. Our methods build on a general formulation of contamination as exposure misclassification due to missing network edges, and provide bias-corrected estimators for both indirect and direct effects. Our bias-corrected estimators rely on two types of sensitivity parameters. The first characterizes the magnitude of contamination. The second, specific to the direct effect, represents the ratio of the average direct effect among egos exposed to at least two treated neighbor to those not exposed. We provided practical guidelines for specifying these sensitivity parameters and described two sensitivity analysis approaches: GSA and PBA.

Our re-analysis of the HPTN 037 trial underscores the consequences of unmeasured contamination in ENRTs. Our findings demonstrate that standard estimators can severely underestimate the indirect effect while slightly overestimating the direct effect. For policymakers, this highlights that peer-driven interventions may be substantially more effective than naive estimates suggest, altering the cost-benefit calculus for deploying network-based public health strategies.

A limitation of our design-based framework is the focus on sample-level causal estimands (1) and (2), which restrict our inference to the finite sample of recruited egos and alters rather than the broader population. Extensions to population-level estimands require specifying the exact mechanism of the egocentric network sampling, such as the inclusion probabilities of the egos. However, many ENRTs, including the HPTN 037 study, involve hard-to-reach populations where egos are typically recruited via convenience sampling rather than a strict probability sample. While recent methodological work has considered population-level estimands in ENRTs, doing so generally relies on the assumption of simple random sampling of units from the population (Fang et al. 2025). Consequently, our focus on sample-level estimands reflects a methodological trade-off: we prioritize robust internal validity and assumption-lean causal inference within the sample, without imposing strong unverifiable conditions on the recruitment process.

A natural alternative to our sensitivity framework might be to explicitly infer the missing alter-ego and ego-ego edges by assuming a stochastic generative model for the population network $ \boldsymbol{A} $ and estimating its parameters from the observed network $ \widetilde{\boldsymbol{A}} $. However, this approach presents significant challenges. First, it requires both positing a valid population-level network model and specifying the egocentric sampling mechanism. Second, even if these mechanisms could be specified, a fundamental challenge remains. Fitting a model to a network of * n * nodes does not necessarily recover the parameters governing the population network of * N * nodes. Assuming that edge formation probabilities scale directly from a sample to the population requires the network model to satisfy properties such as consistency under sampling (Shalizi and Rinaldo 2013; Crane and Dempsey 2021). Our design-based framework coupled with sensitivity analysis offers a reliable alternative. By treating the missing edges as fixed but uncertain, we can quantify the impact of contamination while bypassing the need to posit assumptions on the data-generating and sampling processes.

We assumed a two-level exposure mapping specification, indicating whether a unit is connected to at least one treated neighbor (Assumption 2). This specification is sensible when the treatment effect is saturated at one treated neighbor (regardless of who it is). That is, when exposure to additional treated neighbors does not further affect the outcome. In Supplement B, we extend our methods to a three-level exposure mapping specification, distinguishing between no treated neighbors, one treated neighbor, and two-or-more treated neighbors. In this case, for example, an alter with an observed exposure level of one treated neighbor may have a true exposure level of one or two-or-more treated neighbors. We limit our analysis for the indirect effect * IE *, and derive bias expressions for the naive estimator $ \widehat{IE} $. We also provide bias-corrected estimators that depend on an additional sensitivity parameter that captures the marginal unit-level exposure effect. We show that the two-level exposure mapping model is a special case of the three-level model with saturation of effect after the first exposure.

The exposure mapping specification we took in this paper relies on a first-order neighborhood interference assumption, restricting the diffusion of treatment effects to direct neighbors. A valuable direction for future research is relaxing this specification to accommodate higher-order interference. For example, under second-order interference, an alter could be indirectly affected by the treatment through a connection to another alter in a treated ego-network. In such settings, an ego might also have indirect exposure if its recruited alters are connected to other treated egos. Adapting our proposed sensitivity analysis framework to these scenarios would require specifying sensitivity parameters for latent alter–alter edges across different ego-networks. While conceptually natural, this extension would introduce notable combinatorial complexity to the computation of the exposure probabilities.

ENRT is an attractive design that allows for the estimation of spillovers and the indirect effect without the requirement for sociocentric network measurements. It enables researchers to estimate causal effects under interference with limited network data. However, extending these designs to observational studies where units are sampled from a larger network (e.g., through egocentric sampling), but treatments are not randomly assigned by the researchers, is a more challenging problem. The primary concern is that non-recruited units can be treated as well, leading to treatment interference between recruited and non-recruited units. Since information on the edges connecting such units in the population is rarely available, addressing interference in observational studies with egocentric sampling remains a significant challenge.

## Supplementary Material

kxag031_Supplementary_Data

## Data Availability

Analysis code applied to a simulated dataset similar to the design of HPTN 037 can be found at https://github.com/barwein/ENRT\_SA. The HPTN 037 study datasets are publicly available and can be requested from the Statistical Center for HIV/AIDS Research and Prevention through https://atlas.scharp.org/project/home/begin.view.
