# Supplementary material for: Sensitivity analysis for contamination in egocentric-network randomized trials with interference
Source: Biostatistics. 2026 Aug 12;27(1):kxag031. doi: 10.1093/biostatistics/kxag031 (PMC13461678; doi:10.1093/biostatistics/kxag031)
Supplement: Supplementary file 1 [file kxag031_supplementary_data.zip › biosts-26067-File007.pdf]

Appendix for "Sensitivity analysis for contamination in egocentric-network randomized trials with interference"

Bar Weinstein* and Daniel Nevo†
Department of Statistics and Operations Research, Tel Aviv University

June 7, 2026

Appendix – Table of Contents

A Proofs and technical details **2**
A.1 Proof of Proposition 1 2
A.2 Exposure probabilities of alters 4
A.3 Proof of Proposition 2 4
A.4 Proof of Proposition 3 5
A.5 Direction of bias relative to the null 6
A.6 Direction and magnitude of bias-corrected estimators 7
A.7 Variance estimation and asymptotic distribution (no cross-fitting) 7
A.8 Augmented estimation via cross-fitting 10
A.9 Relative risk estimand and estimators 12
A.10 Relaxing Assumption 4 13
A.11 Estimating the sensitivity parameters with internal validation data 15

B Extension to three-level exposure mapping **17**
B.1 Preliminaries 17
B.2 Bias of the naive estimator 18
B.3 Bias-corrected estimator 20
B.4 Relation to the two-level exposure model 21
B.5 Direction of bias, sign preservation, and effect magnitude 21
B.6 Sensitivity analysis 21

C Probabilistic bias analysis **22**

D Simulations **23**

E Data analysis **26**

F Results of the example analysis code **29**

*Corresponding author, barwein@mail.tau.ac.il.
†This work was supported by the Israel Science Foundation (ISF grant No. 2300/25); BW is supported by the Data Science Fellowship granted by the Israeli Council for Higher Education. We thank Dr. Ashley Buchanan for sharing code and assisting in the analysis of the HPTN 037 study. The authors report no conflict of interest.

A Proofs and technical details

A.1 Proof of Proposition 1

Recall that the HT estimator of the indirect effect based on the observed data (3) is

$$\widehat{IE} = \frac{1}{n_a} \sum_{i \in \mathcal{R}_a} \left[\frac{\mathbb{I}\{\widetilde{F}_i = 1\} Y_i}{p_z} - \frac{\mathbb{I}\{\widetilde{F}_i = 0\} Y_i}{1 - p_z} \right]$$

By the assumed experimental design (Assumption 1), the probability that an alter is assigned to treatment is zero, that is, $\Pr(Z_i = 1 \mid i \in \mathcal{R}_a) = 0$. In addition, by design Z_i and $\widetilde{F}_i = F(\boldsymbol{Z}_{-i}, \widetilde{\boldsymbol{A}})$ are independent. Thus, for an alter $i \in \mathcal{R}_a$, we have $\mathbb{I}\{\widetilde{F}_i = f\} = \mathbb{I}\{Z_i = 0, \widetilde{F}_i = f\}$ with probability one. Starting with the first inner term, for $i \in \mathcal{R}_a$,

$$\begin{aligned}
&\mathbb{E}_{\boldsymbol{Z}} \left[\frac{\mathbb{I}\{\widetilde{F}_i = 1\} Y_i}{p_z} \right] \\
&\overset{(i)}{=} \frac{1}{p_z} \mathbb{E}_{\boldsymbol{Z}} \left[\mathbb{I}\{F_i = 1, \widetilde{F}_i = 1\} Y_i(0,1) + \mathbb{I}\{F_i = 0, \widetilde{F}_i = 1\} Y_i(0,0) \right] \\
&\overset{(ii)}{=} \frac{\Pr(F_i = 1, \widetilde{F}_i = 1 \mid i \in \mathcal{R}_a)}{p_z} Y_i(0,1) + \frac{\Pr(F_i = 0, \widetilde{F}_i = 1 \mid i \in \mathcal{R}_a)}{p_z} Y_i(0,0) \\
&\overset{(iii)}{=} \Pr(F_i = 1 \mid \widetilde{F}_i = 1, i \in \mathcal{R}_a) Y_i(0,1) + \Pr(F_i = 0 \mid \widetilde{F}_i = 1, i \in \mathcal{R}_a) Y_i(0,0) \\
&\overset{(iv)}{=} Y_i(0,1),
\end{aligned} \tag{A.1}$$

where (i) follows since $Z_i = 0$ with probability one for alters and consistency (Assumption 3) that connects the observed outcomes Y_i to the potential outcomes $Y_i(z, f)$; (ii) from taking expectation with respect to the design $\boldsymbol{Z}$ (recalling that the distribution of $\boldsymbol{Z}$ in this paper is conditional on the sample); (iii) simply uses that p_z is equal to $\Pr(\widetilde{F}_i = 1 \mid i \in \mathcal{R}_a)$ and $\frac{\Pr(F_i = f, \widetilde{F}_i = 1 \mid i \in \mathcal{R}_a)}{\Pr(\widetilde{F}_i = 1 \mid i \in \mathcal{R}_a)} = \Pr(F_i = f \mid \widetilde{F}_i = 1, i \in \mathcal{R}_a)$; and (iv) follows since $\Pr(F_i = 1 \mid \widetilde{F}_i = 1, i \in \mathcal{R}_a) = 1$. To see this, note that $\sum_{j \neq i} Z_j \widetilde{A}_{ij} \le \sum_{j \neq i} Z_j A_{ij}$ with probability one, which implies that $\widetilde{F}_i \le F_i$ with probability one as well. Since F_i is binary, if $\widetilde{F}_i = 1$, it must also be $F_i = 1$.

Moving to the second inner term, a similar derivation yields

$$\begin{aligned}
\mathbb{E}_{\boldsymbol{Z}} \left[\frac{\mathbb{I}\{\widetilde{F}_i = 0\} Y_i}{1 - p_z} \right] &= \Pr(F_i = 1 \mid \widetilde{F}_i = 0, i \in \mathcal{R}_a) Y_i(0,1) \\
&\quad + \Pr(F_i = 0 \mid \widetilde{F}_i = 0, i \in \mathcal{R}_a) Y_i(0,0).
\end{aligned}$$

By Bayes' rule,

$$\begin{aligned}
\Pr(F_i = 0 \mid \widetilde{F}_i = 0, i \in \mathcal{R}_a) &= \frac{\Pr(\widetilde{F}_i = 0 \mid F_i = 0, i \in \mathcal{R}_a) \Pr(F_i = 0 \mid i \in \mathcal{R}_a)}{\Pr(\widetilde{F}_i = 0 \mid i \in \mathcal{R}_a)} \\
&= \frac{\Pr(F_i = 0 \mid i \in \mathcal{R}_a)}{\Pr(\widetilde{F}_i = 0 \mid i \in \mathcal{R}_a)} \\
&= \frac{1 - \pi_i^a}{1 - p_z},
\end{aligned} \tag{A.2}$$

as $\Pr(\widetilde{F}_i = 0 \mid F_i = 0, i \in \mathcal{R}_a) = 1$ since $\widetilde{F}_i \leq F_i$ with probability one. Setting $\Pr(\widetilde{F}_i = 1 \mid F_i = 0, i \in \mathcal{R}_a) = 1 - \Pr(\widetilde{F}_i = 0 \mid F_i = 0, i \in \mathcal{R}_a)$ and rearranging terms yields

$$\mathbb{E}_{\boldsymbol{Z}}\left[\frac{\mathbb{I}\{\widetilde{F}_i = 0\}Y_i}{1-p_z}\right] = \frac{(\pi_i^a - p_z)\, Y_i(0,1) + (1-\pi_i^a)\, Y_i(0,0)}{1-p_z}. \tag{A.3}$$

Consequently, combining (A.1) and (A.3),

$$\begin{aligned}
\mathbb{E}_{\boldsymbol{Z}}\left[\widehat{IE}\right] &= \frac{1}{n_a}\sum_{i\in\mathcal{R}_a}\left[Y_i(0,1) - \frac{(\pi_i^a - p_z)\, Y_i(0,1) + (1-\pi_i^a)\, Y_i(0,0)}{1-p_z}\right] \\
&= \frac{1}{n_a}\sum_{i\in\mathcal{R}_a}\frac{1-\pi_i^a}{1-p_z}\left[Y_i(0,1) - Y_i(0,0)\right] \\
&= IE + \frac{1}{n_a}\sum_{i\in\mathcal{R}_a}\frac{p_z-\pi_i^a}{1-p_z}\left[Y_i(0,1) - Y_i(0,0)\right],
\end{aligned} \tag{A.4}$$

which completes the proof of the first part of Proposition 1.

Moving to the direct effect estimator. Recall that the HT estimator based on the observed data (4) is

$$\widehat{DE} = \frac{1}{n_e}\sum_{i\in\mathcal{R}_e}\left[\frac{\mathbb{I}\{Z_i = 1\}Y_i}{p_z} - \frac{\mathbb{I}\{Z_i = 0\}Y_i}{1-p_z}\right].$$

By the assumed experimental design (Assumption 1), the observed exposure of all egos is zero $\Pr(\widetilde{F}_i = 0 \mid i \in \mathcal{R}_e) = 1$, and among the egos $i \in \mathcal{R}_e$, we have that $\mathbb{I}\{Z_i = z\} = \mathbb{I}\{Z_i = z, \widetilde{F}_i = 0\}$ with probability one. The expectation of the first inner term of $\widehat{DE}$, for $i \in \mathcal{R}_e$, is as follows.

$$\begin{aligned}
\mathbb{E}_{\boldsymbol{Z}}\left[\frac{\mathbb{I}\{Z_i = 1\}Y_i}{p_z}\right] &\overset{(i)}{=} \mathbb{E}_{\boldsymbol{Z}}\left[\frac{\mathbb{I}\{Z_i = 1, \widetilde{F}_i = 0\}Y_i}{p_z}\right] \\
&\overset{(ii)}{=} \frac{1}{p_z}\mathbb{E}_{\boldsymbol{Z}}\left[\mathbb{I}\{Z_i = 1, F_i = 1, \widetilde{F}_i = 0\}Y_i(1,1)\right] \\
&\quad + \frac{1}{p_z}\mathbb{E}_{\boldsymbol{Z}}\left[\mathbb{I}\{Z_i = 1, F_i = 0, \widetilde{F}_i = 0\}Y_i(1,0)\right] \\
&\overset{(iii)}{=} \Pr(F_i = 1, \widetilde{F}_i = 0 \mid i \in \mathcal{R}_e)Y_i(1,1) \\
&\quad + \Pr(F_i = 0, \widetilde{F}_i = 0 \mid i \in \mathcal{R}_e)Y_i(1,0) \\
&\overset{(iv)}{=} \Pr(F_i = 0 \mid i \in \mathcal{R}_e)Y_i(1,1) \\
&\quad + \Pr(F_i = 1 \mid i \in \mathcal{R}_e)Y_i(1,0),
\end{aligned}$$

where (i) follows from the explanation described above; (ii) from consistency (Assumption 3); (iii) since Z_i is independent of both F_i and $\widetilde{F}_i$ by Assumption 1, and $\Pr(Z_i = 1 \mid i \in \mathcal{R}_e) = p_z$; and (iv) since $\widetilde{F}_i$ is equal to 0 with probability one. Recalling that $\pi_i^e = \Pr(F_i = 1 \mid i \in \mathcal{R}_e)$ and plugging it into the above expectation yields

$$\mathbb{E}_{\boldsymbol{Z}}\left[\frac{\mathbb{I}\{Z_i = 1\}Y_i}{p_z}\right] = \pi_i^e Y_i(1,1) + (1-\pi_i^e)Y_i(1,0). \tag{A.5}$$

Replacing $Z_i = 1$ with $Z_i = 0$ and p_z with $1 - p_z$ above, yields similar results. Specifically, for $z = 0$ we have

$$\mathbb{E}_{\boldsymbol{Z}}\left[\frac{\mathbb{I}\{Z_i = 0\}Y_i}{1-p_z}\right] = \pi_i^e Y_i(0,1) + (1-\pi_i^e)Y_i(0,0). \tag{A.6}$$

Plugging (A.5) and (A.6) into $\mathbb{E}_{\boldsymbol{Z}}\left[\widehat{DE}\right]$ yields

$$\begin{aligned}\mathbb{E}_{\boldsymbol{Z}}\left[\widehat{DE}\right] &= \frac{1}{n_e}\sum_{i\in\mathcal{R}_e}\left[\pi_i^e\left\{Y_i(1,1)-Y_i(0,1)\right\}+(1-\pi_i^e)\left\{Y_i(1,0)-Y_i(0,0)\right\}\right] \\ &= DE+\frac{1}{n_e}\sum_{i\in\mathcal{R}_e}\pi_i^e\left[\left\{Y_i(1,1)-Y_i(0,1)\right\}-\left\{Y_i(1,0)-Y_i(0,0)\right\}\right],\end{aligned} \tag{A.7}$$

which completes the second part of Proposition 1. □

A.2 Exposure probabilities of alters

Alter $i\in\mathcal{R}_a$ is exposed ($F_i=1$) if its ego $e(i)$ or at least one of the other recruited egos connected to i in the population network $\boldsymbol{A}$ is treated. We have

$$\begin{aligned}\pi_i^a &= \Pr(F_i=1\mid i\in\mathcal{R}_a) \\ &= \Pr(F_i=1\mid Z_{e(i)}=1, i\in\mathcal{R}_a)\Pr(Z_{e(i)}=1\mid i\in\mathcal{R}_a) \\ &+ \Pr(F_i=1\mid Z_{e(i)}=0, i\in\mathcal{R}_a)\Pr(Z_{e(i)}=0\mid i\in\mathcal{R}_a),\end{aligned} \tag{A.8}$$

but $\Pr(F_i=1\mid Z_{e(i)}=1, i\in\mathcal{R}_a)=1$ and $\Pr(Z_{e(i)}=1\mid i\in\mathcal{R}_a)=p_z\mathbb{I}\{e(i)\in\mathcal{R}_e\}=p_z$ by Assumptions 1 and 2. Since only recruited egos have a non-zero probability of being assigned to treatment, then for alter $i\in\mathcal{R}_a$, $F_i=\sum_{j\neq i}Z_jA_{ij}=\sum_{j\in\mathcal{R}_e}Z_jA_{ij}$ with probability one. Therefore,

$$\begin{aligned}\Pr(F_i=0\mid Z_{e(i)}=0, i\in\mathcal{R}_a) &= \Pr\Big(\sum_{j\in\mathcal{R}_e}Z_jA_{ij}=0\mid Z_{e(i)}=0, i\in\mathcal{R}_a\Big) \\ &= \Pr\Big(\sum_{j\in\mathcal{R}_e\setminus e(i)}Z_jA_{ij}=0\mid i\in\mathcal{R}_a\Big) \\ &= \prod_{j\in\mathcal{R}_e\setminus\{e(i)\}}\left(1-p_z\rho_{ij}^a\right).\end{aligned}$$

Substituting $\Pr(F_i=1\mid Z_{e(i)}=0, i\in\mathcal{R}_a)=1-\Pr(F_i=0\mid Z_{e(i)}=0, i\in\mathcal{R}_a)$ in (A.8) yields (14).

A.3 Proof of Proposition 2

The bias-corrected estimator is

$$\widehat{IE}_{adj}=\frac{1}{n_a}\sum_{i\in\mathcal{R}_a}\frac{1-p_z}{1-\pi_i^a}\left[\frac{\mathbb{I}\{\widetilde{F}_i=1\}Y_i}{p_z}-\frac{\mathbb{I}\{\widetilde{F}_i=0\}Y_i}{1-p_z}\right].$$

As the weights $\frac{1-p_z}{1-\pi_i^a}$ are fixed and therefore independent of $\boldsymbol{Z}$, taking the expectation $\mathbb{E}_{\boldsymbol{Z}}\left[\widehat{IE}_{adj}\right]$ will result in the second row of (A.4) where the terms $\frac{1-\pi_i^a}{1-p_z}$ and $\frac{1-p_z}{1-\pi_i^a}$ are multiplied and therefore canceled. That implies $\mathbb{E}_{\boldsymbol{Z}}\left[\widehat{IE}_{adj}\right]=IE$ and completes the proof of Proposition 2. □

A.4 Proof of Proposition 3

Recall that (7) states that

$$\overline{\Delta}(1) = \kappa\overline{\Delta}(0),$$

where $\overline{\Delta}(f) = \frac{1}{n_e}\sum_{i\in\mathcal{R}_e}\Delta_i(f)$ and $\Delta_i(f) = Y_i(1,f) - Y_i(0,f)$. The bias-adjusted direct effect estimator is

$$\widehat{DE}_{adj} = \frac{1}{1+\overline{\pi}^e(\kappa-1)}\widehat{DE} = \frac{1}{u_e}\sum_{i\in\mathcal{R}_e}\left[\frac{\mathbb{I}\{Z_i=1\}Y_i}{p_z} - \frac{\mathbb{I}\{Z_i=0\}Y_i}{1-p_z}\right],$$

where $u_e = n_e\left[1+\overline{\pi}^e(\kappa-1)\right]$ and $\overline{\pi}^e = n_e^{-1}\sum_{i\in\mathcal{R}_e}\pi_i^e$ is the average probability of exposure among the egos.

The inner product of two vectors $\boldsymbol{a},\boldsymbol{b}\in\mathbb{R}^m$ can be written as $\frac{1}{m}\sum_i a_ib_i = \overline{a}\overline{b} + S_{\boldsymbol{a},\boldsymbol{b}}$ where $S_{\boldsymbol{a},\boldsymbol{b}} = \frac{1}{m}\sum_i(a_i-\overline{a})(b_i-\overline{b})$. Since both κ and π_i^e are fixed and therefore independent of $\boldsymbol{Z}$, we can use (A.7) and write the expectation of the naive estimator as

$$\begin{aligned}\mathbb{E}_{\boldsymbol{Z}}\left[\widehat{DE}\right] &= DE + \frac{1}{n_e}\sum_{i\in\mathcal{R}_e}\pi_i^e\left[\{Y_i(1,1)-Y_i(0,1)\} - \{Y_i(1,0)-Y_i(0,0)\}\right]\\ &= DE + \frac{1}{n_e}\sum_{i\in\mathcal{R}_e}\pi_i^e\left[\Delta_i(1)-\Delta_i(0)\right] \qquad \text{(A.9)}\\ &= DE + \overline{\pi}^e\left(\overline{\Delta}(1)-\overline{\Delta}(0)\right) + S_{\boldsymbol{\pi}^e,\boldsymbol{\Delta(1)}} - S_{\boldsymbol{\pi}^e,\boldsymbol{\Delta(0)}}\\ &= \left[1+\overline{\pi}^e(\kappa-1)\right]DE + S_{\boldsymbol{\pi}^e,\boldsymbol{\Delta(1)}} - S_{\boldsymbol{\pi}^e,\boldsymbol{\Delta(0)}},\end{aligned}$$

where we used $\overline{\Delta}(1) = \kappa\overline{\Delta}(0)$ and the fact that $\overline{\Delta}(0) = DE$. Thus, the expected value of the bias-corrected estimator is

$$\mathbb{E}_{\boldsymbol{Z}}\left[\widehat{DE}_{adj}\right] = DE + \frac{S_{\boldsymbol{\pi}^e,\boldsymbol{\Delta(1)}} - S_{\boldsymbol{\pi}^e,\boldsymbol{\Delta(0)}}}{1+\overline{\pi}^e(\kappa-1)}.$$

Finally, under Assumption 4, $S_{\boldsymbol{\pi}^e,\boldsymbol{\Delta(1)}} = S_{\boldsymbol{\pi}^e,\boldsymbol{\Delta(0)}}$, and we obtain $\mathbb{E}_{\boldsymbol{Z}}\left[\widehat{DE}_{adj}\right] = DE$. That completes the proof of Proposition 3. □

In the case of homogeneous exposure probabilities $\pi_i^e = \pi^e$ for all $i\in\mathcal{R}_e$ for some constant $\pi^e\in[0,1]$, we have $S_{\boldsymbol{\pi}^e,\boldsymbol{\Delta(1)}} = S_{\boldsymbol{\pi}^e,\boldsymbol{\Delta(0)}} = 0$, therefore $\widehat{DE}_{adj}$ is an unbiased estimator of DE.

Conditions under which the naive HT estimator is unbiased. If $\pi_i^e = 0$ for all $i\in\mathcal{R}_e$ or $\kappa = 1$, the bias-corrected estimator $\widehat{DE}_{adj}$ reduces to the naive HT estimator $\widehat{DE}$. If $\pi_i^e = 0$, then from Proposition 1 the naive HT estimator will be an unbiased estimator of DE. If $\kappa = 1$, we have two options

1. If the exposure probabilities are homogeneous ($\pi_i^e = \pi^e$), then $S_{\boldsymbol{\pi}^e,\boldsymbol{\Delta(1)}} = S_{\boldsymbol{\pi}^e,\boldsymbol{\Delta(0)}} = 0$ and $\widehat{DE}$ is an unbiased estimator of DE. That will be true even if there is some contamination between the egos, i.e., if $\pi^e > 0$.

2. As described in the main text, under the saturated potential outcome model

$$Y_i(z,f) = \beta_{0i} + \beta_{1i}z + \beta_{2i}f + \beta_{3i}zf,$$

Assumption 4 reduces to

$$S_{\boldsymbol{\pi}^e,\boldsymbol{\beta}_3} = \frac{1}{n_e}\sum_{i\in\mathcal{R}_e}(\pi_i^e - \overline{\pi}^e)(\beta_{3i} - \overline{\beta_3}) = 0.$$

Setting $\kappa = 1$ implies that $\sum_{i\in\mathcal{R}_e}\beta_{3i} = 0$. Thus, $\widehat{DE}$ will be an unbiased estimator of DE if $\sum_{i\in\mathcal{R}_e}\pi_i^e\beta_{3i} = 0$.

A.5 Direction of bias relative to the null

We now show the direction of bias of the naive estimators $\widehat{IE}$ and $\widehat{DE}$ relative to the null of no effects.

Indirect effect estimator Starting with $\widehat{IE}$. If $Y_i(0,1) \geq Y_i(0,0)$ for all $i \in \mathcal{R}_a$ such that $IE \geq 0$, then since $p_z - \pi_i^a \leq 0$, from (A.4), we have $0 \leq \mathbb{E}_{\boldsymbol{Z}}\left[\widehat{IE}\right] \leq IE$. Vice versa, if $Y_i(0,1) \leq Y_i(0,0)$ for all $i \in \mathcal{R}_a$, then $IE \leq 0$ and $0 \geq \mathbb{E}_{\boldsymbol{Z}}\left[\widehat{IE}\right] \geq IE$. That is, the estimator $\widehat{IE}$ is, in expectation, biased towards the null of no indirect effect. That will hold in both the homogeneous and heterogeneous scenarios of ρ_{ij}^a, as $p_z - \pi_i^a \leq 0$ for all $i \in \mathcal{R}_a$.

Direct effect estimator We show the direction of bias relative to the null of the naive direct effect estimator $\widehat{DE}$ using the representation κ as defined in (7). From (A.9)

$$\mathbb{E}_{\boldsymbol{Z}}\left[\widehat{DE}\right] = \left[1 + \overline{\pi}^e(\kappa - 1)\right]DE + S_{\boldsymbol{\pi}^e,\boldsymbol{\Delta(1)}} - S_{\boldsymbol{\pi}^e,\boldsymbol{\Delta(0)}}.$$

Under Assumption 4, the covariance terms are equal, thus

$$\mathbb{E}_{\boldsymbol{Z}}\left[\widehat{DE}\right] = \left[1 + \overline{\pi}^e(\kappa - 1)\right]DE$$

Since $\overline{\pi}^e \geq 0$, we have

- If $DE \geq 0$

$$\begin{cases} \mathbb{E}_{\boldsymbol{Z}}\big[\widehat{DE}\big] \geq DE \geq 0, & \kappa > 1, \\ DE \geq \mathbb{E}_{\boldsymbol{Z}}\big[\widehat{DE}\big] \geq 0, & \kappa \in \left(1 - \frac{1}{\overline{\pi}^e}, 1\right), \\ \mathbb{E}_{\boldsymbol{Z}}\big[\widehat{DE}\big] \leq 0, & \kappa < 1 - \frac{1}{\overline{\pi}^e}. \end{cases}$$

- If $DE \leq 0$

$$\begin{cases} \mathbb{E}_{\boldsymbol{Z}}\big[\widehat{DE}\big] \leq DE \leq 0, & \kappa > 1, \\ DE \leq \mathbb{E}_{\boldsymbol{Z}}\big[\widehat{DE}\big] \leq 0, & \kappa \in \left(1 - \frac{1}{\overline{\pi}^e}, 1\right), \\ \mathbb{E}_{\boldsymbol{Z}}\big[\widehat{DE}\big] \geq 0, & \kappa < 1 - \frac{1}{\overline{\pi}^e}. \end{cases}$$

Therefore, the estimator $\widehat{DE}$ is biased *away* from the null of no direct effect if $\kappa > 1$, biased *towards* the null if $\kappa \in \left(1 - \frac{1}{\overline{\pi}^e}, 1\right)$, and changes sign if $\kappa < 1 - \frac{1}{\overline{\pi}^e}$. Since π_i^e is typically small, then $1 - \frac{1}{\overline{\pi}^e}$ is a large negative number, and it is unlikely that κ is smaller than that value in practice. Thus, in general, we expect that the estimator $\widehat{DE}$ is biased away from the null if $\kappa > 1$ and towards the null if $\kappa < 1$ for many realistic scenarios.

A.6 Direction and magnitude of bias-corrected estimators

We now derive the direction and effect magnitude of the bias-corrected estimators $\widehat{IE}_{adj}$ and $\widehat{DE}_{adj}$ compared to their respective naive estimators $\widehat{IE}$ and $\widehat{DE}$.

Indirect effect estimators. Eq. (14) implies that $\pi_i^a \geq p_z$, therefore $\frac{1-p_z}{1-\pi_i^a} \geq 1$. If $\pi_i^a = \pi^a$ for all $i \in \mathcal{R}_a$, that is, the same for all alters, then $\widehat{IE}_{adj} = \frac{1-p_z}{1-\pi^a}\widehat{IE}$. Thus, $\text{sign}(\widehat{IE}_{adj}) = \text{sign}(\widehat{IE})$ and

$$\begin{cases} \widehat{IE}_{adj} \geq \widehat{IE}, & \widehat{IE} > 0, \\ \widehat{IE}_{adj} \leq \widehat{IE}, & \widehat{IE} < 0. \end{cases}$$

Direct effect estimators. Recall that $\widehat{DE}_{adj} = \frac{1}{1+\overline{\pi}^e(\kappa-1)}\widehat{DE}$. Thus, if $1 + \overline{\pi}^e(\kappa - 1) > 1 \Rightarrow \kappa > 1 - \frac{1}{\overline{\pi}^e}$, then $\text{sign}(\widehat{DE}_{adj}) = \text{sign}(\widehat{DE})$. Otherwise, if $\kappa < 1 - \frac{1}{\overline{\pi}^e}$, then $\text{sign}(\widehat{DE}_{adj}) = -\text{sign}(\widehat{DE})$. However, for example, if $\overline{\pi}^e \approx 0.01$, $1 - \frac{1}{\overline{\pi}^e} \approx -99$, and it is unlikely that κ is smaller than that value in practice. So in general, we expect $\text{sign}(\widehat{DE}_{adj}) = \text{sign}(\widehat{DE})$. Moreover, if $\kappa > 1$ then

$$\begin{cases} \widehat{DE}_{adj} < \widehat{DE}, & \widehat{DE} > 0, \\ \widehat{DE}_{adj} > \widehat{DE}, & \widehat{DE} < 0, \end{cases}$$

so the adjusted estimator is, in magnitude, closer to zero than the unadjusted estimator. Vice versa, if $\kappa \in \left(1 - \frac{1}{\overline{\pi}^e}, 1\right)$ then

$$\begin{cases} \widehat{DE}_{adj} > \widehat{DE}, & \widehat{DE} > 0, \\ \widehat{DE}_{adj} < \widehat{DE}, & \widehat{DE} < 0. \end{cases}$$

Thus, the adjusted estimator is, in magnitude, further away from zero than the unadjusted estimator.

A.7 Variance estimation and asymptotic distribution (no cross-fitting)

We derive variance estimators for the non-augmented (but bias-adjusted) estimators.

Indirect effect estimator. Starting with $\widehat{IE}_{adj}$. Let $r_i^a = \frac{1-p_z}{1-\pi_i^a}\left[\frac{\mathbb{I}\{\widetilde{F}_i=1\}Y_i}{p_z} - \frac{\mathbb{I}\{\widetilde{F}_i=0\}Y_i}{1-p_z}\right]$ such that $\widehat{IE}_{adj} = \frac{1}{n_a}\sum_{i\in\mathcal{R}_a} r_i^a$. By the ENRT design, alters in the same ego-network all have the same treatment assignments and exposure status. Thus, we can calculate the variance by using the aggregated outcomes in each ego-network. To this end, we can write

$$\begin{aligned} \widehat{IE}_{adj} &= \frac{1}{n_a}\sum_{i\in\mathcal{R}_e}\sum_{j\in\mathcal{R}_a:e(j)=i} r_j^a \\ &= \frac{1}{n_a}\sum_{i\in\mathcal{R}_e} T_i \\ &= \frac{n_e}{n_a}\overline{T}, \end{aligned}$$

where $T_i = \sum_{j \in \mathcal{R}_a : e(j)=i} r_j^a$ and $\overline{T} = \frac{1}{n_e} \sum_{i \in \mathcal{R}_e} T_i$ is the average over ego-networks. Therefore, the variance is $V_{\boldsymbol{Z}}\left(\widehat{IE}_{adj}\right) = \frac{n_e^2}{n_a^2} V_{\boldsymbol{Z}}\left(\overline{T}\right)$, which can be conservatively estimated with the design-based variance estimator (Ding, 2024)

$$\widehat{V}_{\boldsymbol{Z}}\left(\widehat{IE}_{adj}\right) = \frac{1}{n_a^2} \sum_{i \in \mathcal{R}_e} \left(T_i - \overline{T}\right)^2.$$

Under standard regularity conditions, the estimator $\widehat{IE}_{adj}$ is asymptotically normally distributed with mean IE (Ding, 2024). This motivates a Wald-type confidence interval for IE based on the variance estimator $\widehat{V}_{\boldsymbol{Z}}\left(\widehat{IE}_{adj}\right)$

$$\widehat{IE}_{adj} \pm z_{1-\alpha/2} \sqrt{\widehat{V}_{\boldsymbol{Z}}\left(\widehat{IE}_{adj}\right)},$$

where $z_{1-\alpha/2}$ is the $1-\alpha/2$ upper quantile of a standard normal distribution. However, since the estimator $\widehat{V}_{\boldsymbol{Z}}\left(\widehat{IE}_{adj}\right)$ is a conservative estimator of $V_{\boldsymbol{Z}}\left(\widehat{IE}_{adj}\right)$, constructing confidence intervals based on it results in a conservative confidence interval with coverage rate of at least $1-\alpha$.

Direct effect estimator. Moving to $\widehat{DE}_{adj}$. Let $r_i^e = \frac{\mathbb{I}\{Z_i=1\}Y_i}{p_z} - \frac{\mathbb{I}\{Z_i=0\}Y_i}{1-p_z}$ such that $\widehat{DE}_{adj} = \frac{1}{u_e} \sum_{i \in \mathcal{R}_e} r_i^e$. By the ENRT design, treatment assignment is independent across egos. We can write the variance of $\widehat{DE}_{adj}$ as

$$V_{\boldsymbol{Z}}\left(\widehat{DE}_{adj}\right) = \frac{1}{u_e^2} \sum_{i \in \mathcal{R}_e} V_{\boldsymbol{Z}}(r_i^e) + \frac{1}{u_e^2} \sum_{i,j \in \mathcal{R}_e : i \neq j} \text{Cov}_{\boldsymbol{Z}}(r_i^e, r_j^e), \tag{A.10}$$

where $V_{\boldsymbol{Z}}(r_i^e)$ is the variance of the terms r_i^e w.r.t. the design $\boldsymbol{Z}$. The Neyman-type variance estimator is (Ding, 2024)

$$\widehat{V}_{Neyman}\left(\widehat{DE}_{adj}\right) = \frac{1}{u_e^2} \sum_{i \in \mathcal{R}_e} \left(r_i^e - \widehat{DE}_{adj}\right)^2.$$

This estimator assumes that the terms r_i^e are independent, and is therefore a conservative estimator of the first term in $V_{\boldsymbol{Z}}\left(\widehat{DE}_{adj}\right)$. However, in the presence of contamination, this independence assumption is violated. From the exposure mapping specification (Assumption 2) and consistency (Assumption 3), we can write the terms r_i^e as

$$r_i^e = \frac{Z_i}{p_z}\left[F_i Y_i(1,1) + (1-F_i)Y_i(1,0)\right] - \frac{1-Z_i}{1-p_z}\left[F_i Y_i(0,1) + (1-F_i)Y_i(0,0)\right],$$

where $F_i = F(\boldsymbol{Z}_{-i}, \boldsymbol{A})$ are the true exposures of ego i. Therefore, if egos i and j share an ego neighbor k in the population network $\boldsymbol{A}$, the terms r_i^e and r_j^e will be dependent, as Z_k will influence both F_i and F_j. These dependencies induce pairwise covariances $\text{Cov}_{\boldsymbol{Z}}(r_i^e, r_j^e)$ that are omitted by the Neyman-type estimator $\widehat{V}_{Neyman}\left(\widehat{DE}_{adj}\right)$. Thus, $\widehat{V}_{Neyman}\left(\widehat{DE}_{adj}\right)$ captures only the sum of marginal variances, and ignoring the covariance terms that arise under contamination might lead to underestimation of the overall variance.

To ensure valid inference, we propose a conservative variance correction that accounts for these latent correlations. The terms r_i^e and r_j^e for two egos $i, j \in \mathcal{R}_e$ are correlated if

the egos share a common (latent) neighbor $k \in \mathcal{R}_e$. Let $K_{ij}^{\boldsymbol{A}}(k) = \mathbb{I}\{A_{ik} = 1, A_{jk} = 1\}$ be the indicator that in the population network $\boldsymbol{A}$, the ego $k \in \mathcal{R}_e$ is a common neighbor of both i and j while $k \neq i, j$. Define the number of shared neighbors between ego i and j by in the population network $\boldsymbol{A}$ by

$$K_{ij}^{\boldsymbol{A}} = \sum_{k \in \mathcal{R}_e, k \neq i,j} K_{ij}^{\boldsymbol{A}}(k).$$

We construct a dependency graph $\boldsymbol{G}_{dep}^{\boldsymbol{A}} = \left(\mathcal{R}_e, \boldsymbol{E}_{dep}^{\boldsymbol{A}}\right)$ where an edge exists between two egos $i, j \in \mathcal{R}_e$ if the terms r_i^e and r_j^e are dependent. That is, $\boldsymbol{E}_{dep}^{\boldsymbol{A}}(i,j) = 1$ occurs if $A_{ik} = A_{jk} = 1$ for some other ego $k \in \mathcal{R}_e$. Thus, we can write $\mathbb{I}\{\boldsymbol{E}_{dep}^{\boldsymbol{A}}(i,j) = 1\} = \mathbb{I}\{K_{ij}^{\boldsymbol{A}} > 0\}$. However, the ego-ego edges in $\boldsymbol{A}$ are missing from the observed network $\widetilde{\boldsymbol{A}}$. But we can compute the probability of the dependence under the sensitivity model (Section 3.4). Each of the missing ego-ego edges A_{ik} is postulated to exist independently with probability ρ_{ik}^e. Therefore, taking expectation with regard to the epistemic uncertainty from the sensitivity model yields,

$$\begin{aligned}
\Pr(\boldsymbol{E}_{dep}^{\boldsymbol{A}}(i,j) = 1) &= \Pr(K_{ij}^{\boldsymbol{A}} > 0) \\
&= 1 - \Pr(K_{ij}^{\boldsymbol{A}} = 0) \\
&= 1 - \prod_{k \in \mathcal{R}_e : k \neq i,j} (1 - \rho_{ik}^e \rho_{jk}^e) \\
&= \xi_{ij}.
\end{aligned}$$

As the network $\boldsymbol{A}$ is assumed to be fixed, but partly unknown, the expressions $\mathrm{Cov}_{\boldsymbol{Z}}(r_i^e, r_j^e)$ in (A.10) are essentially conditioned on it. That is, written explicitly, $\mathrm{Cov}_{\boldsymbol{Z}}(r_i^e, r_j^e)$ should be viewed as $\mathrm{Cov}_{\boldsymbol{Z}}(r_i^e, r_j^e \mid \boldsymbol{A})$. As the terms r_i^e and r_j^e are dependent only under the event $\mathbb{I}\{\boldsymbol{E}_{dep}^{\boldsymbol{A}}(i,j) = 1\}$, for a fixed population network $\boldsymbol{A}$, we can write $\mathrm{Cov}_{\boldsymbol{Z}}(r_i^e, r_j^e \mid \boldsymbol{A})$ as $\mathbb{I}\{\boldsymbol{E}_{dep}^{\boldsymbol{A}}(i,j) = 1\}\mathrm{Cov}_{\boldsymbol{Z}}(r_i^e, r_j^e \mid \boldsymbol{A})$. By the Cauchy-Schwarz inequality, we can bound the covariance term

$$\mathbb{I}\{\boldsymbol{E}_{dep}^{\boldsymbol{A}}(i,j) = 1\}|\mathrm{Cov}_{\boldsymbol{Z}}(r_i^e, r_j^e \mid \boldsymbol{A})| \leq \mathbb{I}\{\boldsymbol{E}_{dep}^{\boldsymbol{A}}(i,j) = 1\}\sqrt{V_{\boldsymbol{Z}}(r_i^e)V_{\boldsymbol{Z}}(r_j^e)},$$

where $V_{\boldsymbol{Z}}(r_i^e)$ is the variance of the terms r_i^e w.r.t. the design $\boldsymbol{Z}$. Since $\mathbb{I}\{\boldsymbol{E}_{dep}^{\boldsymbol{A}}(i,j) = 1\}$ is unknown due to the missing ego-ego edges in the observed network, we replace it with its expected value $\Pr(\boldsymbol{E}_{dep}^{\boldsymbol{A}}(i,j) = 1) = \xi_{ij}$ under the sensitivity model. Therefore,

$$\xi_{ij}|\mathrm{Cov}_{\boldsymbol{Z}}(r_i^e, r_j^e \mid \boldsymbol{A})| \leq \xi_{ij}\sqrt{V_{\boldsymbol{Z}}(r_i^e)V_{\boldsymbol{Z}}(r_j^e)}$$

We use the squared residuals $\hat{v}_i = (r_i^e - \widehat{DE}_{adj})^2$ as conservative estimators for the unit-level variances $V_{\boldsymbol{Z}}(r_i^e)$. This yields a conservative estimator for the variance in $\widehat{DE}_{adj}$ due to contamination between ego-networks

$$\widehat{V}_{Conta.}\left(\widehat{DE}_{adj}\right) = \frac{1}{u_e^2}\sum_{i \neq j} \xi_{ij}\sqrt{\hat{v}_i \hat{v}_j}. \tag{A.11}$$

The final corrected variance estimator for $\widehat{DE}_{adj}$ is given by

$$\widehat{V}_{\boldsymbol{Z}}\left(\widehat{DE}_{adj}\right) = \widehat{V}_{Neyman}\left(\widehat{DE}_{adj}\right) + \widehat{V}_{Conta.}\left(\widehat{DE}_{adj}\right). \tag{A.12}$$

We establish the asymptotic normality of $\widehat{DE}_{adj}$ by adapting standard asymptotic normality results for design-based estimators (Ding, 2024) to the dependent data setting induced by our sensitivity model. We present a sketch of a proof based on the Central Limit Theorem for dependency graphs established by Baldi and Rinott (1989). Under the design-based framework, statistical dependence between the summands r_i^e in $\widehat{DE}_{adj}$ arises solely from the random treatment assignments of shared neighbors in the latent population network. Specifically, the dependence arises from the true exposure values $F_i = F_i(\boldsymbol{Z}_{-i}, \boldsymbol{A}_i)$ due to possible contamination under the sensitivity model.

Consider the dependency graph $\boldsymbol{G}_{dep}^{\boldsymbol{A}} = \left(\mathcal{R}_e, \boldsymbol{E}_{dep}^{\boldsymbol{A}}\right)$ presented above. Define the degree of each ego $i \in \mathcal{R}_e$ in this graph by $\mathrm{Deg}_{n_e,i}$, and let $\mathrm{Deg}_{n_e,max} = \max_{i\in\mathcal{R}_e} \mathrm{Deg}_{n_e,i}$ denote the maximum degree. Assume that the terms r_i^e are bounded, and that the variance of the sum converges linearly n_e, i.e., $V_{\boldsymbol{Z}}\left(\sum_{i\in\mathcal{R}_e} r_i^e\right) = \Theta(n_e)$, indicating that the variance of the sum is bounded both above and below by n_e asymptotically. Therefore, Corollary 2 of Baldi and Rinott (1989) implies that the error in the normal approximation is bounded by a term of order $\mathrm{Deg}_{n_e,max}/n_e^{1/4}$. Consequently, the validity of the normal approximation requires that the size of the dependency neighborhoods grows sufficiently slowly relative to the sample size, specifically $\mathrm{Deg}_{n_e,max} = o(n_e^{1/4})$.

In the context of our sensitivity analysis, as the true population network $\boldsymbol{A}$ is only partially known, this imposes a constraint on the postulated latent network in the specified sensitivity model. If the specified probabilities ρ_{ij}^e are sufficiently large such that the latent network become dense (e.g., $\rho_{ij}^e \approx 1$ for all egos $i, j \in \mathcal{R}_e$), this causes the maximal degree of the dependency graph to scale linearly with the sample size ($\mathrm{Deg}_{n_e,max} \approx n_e$), violating the condition $\mathrm{Deg}_{n_e,max} = o(n_e^{1/4})$. Therefore, the reported confidence intervals are valid only for values of the sensitivity parameters consistent with a sufficiently sparse latent network topology.

Following a similar argument to $\widehat{IE}_{adj}$, the Wald-type confidence interval for DE based on the conservative variance estimator $\widehat{V}_{\boldsymbol{Z}}\left(\widehat{DE}_{adj}\right)$

$$\widehat{DE}_{adj} \pm z_{1-\alpha/2}\sqrt{\widehat{V}_{\boldsymbol{Z}}\left(\widehat{DE}_{adj}\right)},$$

results in a conservative confidence interval for DE with coverage of at least $1-\alpha$.

A.8 Augmented estimation via cross-fitting

The augmented estimators combine the outcome Y_i and predicted outcome models for estimating direct and indirect effects. Estimating the outcome models requires cross-fitting. We utilize a two-fold cross-fitting algorithm tailored for design-based estimators (Lu et al., 2025). We also provide variance estimators for the augmented estimators obtained from this cross-fitting procedure. Finally, we describe the asymptotic distribution of the estimators.

We adapt Lu et al. (2025, Algorithm 1) to our setting. Specifically, from Assumption 1 of Bernoulli design for each ego-network, Lu et al. (2025, Proposition 1) motivates the following two-fold cross-fitting algorithm.

1. Randomly split ego-networks into two disjoint parts S_0, S_1 such that $|S_0 \cup S_1| = n$ and $S_0 \cap S_1 = \emptyset$. Specifically, we assign each ego-network (ego and alters) into S_1 with probability 0.5.

2. For $q = 0, 1$ do

(a) Estimate the outcome functions for alters $\widehat{\mu}_i^a(f)$ and egos $\widehat{\mu}_i^e(z)$ using data from the complementary split S_{1-q}.

(b) Predict $\widehat{\mu}_i^a(f)$ and $\widehat{\mu}_i^e(z)$ on the units from S_q.

(c) Estimate IE on units from S_q:

$$\widehat{IE}_{adj[q]}^{aug} = \frac{1}{n_{a[q]}} \sum_{i \in \mathcal{R}_a \cap S_q} \frac{1-p_z}{1-\pi_i^a} \left[D_i^a + \widehat{\mu}_i^a(1) - \widehat{\mu}_i^a(0) \right],$$

where $n_{a[q]} = \sum_{i \in \mathcal{R}_a} \mathbb{I}\{i \in S_q\}$ is the number of alters in split S_q, and where

$$D_i^a = \frac{\mathbb{I}\{\widetilde{F}_i = 1\} \left(Y_i - \widehat{\mu}_i^a(1)\right)}{p_z} - \frac{\mathbb{I}\{\widetilde{F}_i = 0\} \left(Y_i - \widehat{\mu}_i^a(0)\right)}{1-p_z}.$$

(d) Estimate DE on units from S_q:

$$\widehat{DE}_{adj[q]}^{aug} = \frac{1}{u_{e[q]}} \sum_{i \in \mathcal{R}_e \cap S_q} \left[D_i^e + \widehat{\mu}_i^e(1) - \widehat{\mu}_i^e(0) \right],$$

where $u_{e[q]} = n_{e[q]} \left[1 + \overline{\pi}^{e[q]}(\kappa - 1)\right]$ similarly to u_e in Section 3.2, with $n_{e[q]} = \sum_{i \in \mathcal{R}_e} \mathbb{I}\{i \in S_q\}$ and $\overline{\pi}^{e[q]} = \frac{1}{n_{e[q]}} \sum_{i \in \mathcal{R}_e \cap S_q} \pi_i^e$ are the number of egos and the average exposure probability, respectively, in split S_q, and

$$D_i^e = \frac{\mathbb{I}\{Z_i = 1\} \left(Y_i - \widehat{\mu}_i^e(1)\right)}{p_z} - \frac{\mathbb{I}\{Z_i = 0\} \left(Y_i - \widehat{\mu}_i^e(0)\right)}{1-p_z}.$$

3. Combine the estimates across splits

$$\widehat{IE}_{adj}^{aug} = \sum_{q=0,1} \frac{n_{a[q]}}{n_a} \widehat{IE}_{adj[q]}^{aug},$$

$$\widehat{DE}_{adj}^{aug} = \sum_{q=0,1} \frac{u_{e[q]}}{u_e} \widehat{DE}_{adj[q]}^{aug}.$$

Note that the weighting in the combination of the direct effect estimators uses u_e (as defined in Section 3.2) since $u_e = u_{e[0]} + u_{e[1]}$. As the units in the splits S_0 and S_1 are independent (w.r.t. $\boldsymbol{Z}$), variance estimation will also be a combination of variance estimates in each split (Lu et al., 2025). For the indirect effect estimates, we can write the variance estimate in split q by

$$\widehat{V}_{\boldsymbol{Z}}\left(\widehat{IE}_{adj[q]}^{aug}\right) = \frac{1}{n_{a[q]}^2} \sum_{i \in \mathcal{R}_e \cap S_q} \left(T_i^{aug} - \overline{T}_{[q]}^{aug}\right)^2,$$

where $T_i^{aug} = \sum_{j \in \mathcal{R}_a \cap S_q : e(j) = i} \frac{1-p_z}{1-\pi_j^a} D_j^a$ is the average of weighted residuals D_j^a of alter j in ego-network of ego $i \in \mathcal{R}_e$ in split q, and $\overline{T}_{[q]}^{aug} = \frac{1}{n_{e[q]}} \sum_{i \in \mathcal{R}_e \cap S_q} T_i^{aug}$ is the average over ego-networks in split q. The outcome model terms $\widehat{\mu}_i^e(1) - \widehat{\mu}_i^e(0)$ are not included in the variance estimator as they are fixed w.r.t. $\boldsymbol{Z}$ and since the splitting rule ensures independence between the two folds. The combined variance estimator of $\widehat{IE}_{adj}^{aug}$ is

$$\widehat{V}_{\boldsymbol{Z}}\left(\widehat{IE}_{adj}^{aug}\right) = \sum_{q=0,1} \frac{n_{a[q]}^2}{n_a^2} \widehat{V}_{\boldsymbol{Z}}\left(\widehat{IE}_{adj[q]}^{aug}\right). \tag{A.13}$$

For direct effects, the Neyman-type variance estimator in split q is

$$\widehat{V}_{Neyman}\left(\widehat{DE}^{aug}_{adj[q]}\right) = \frac{1}{u^2_{e[q]}} \sum_{i \in \mathcal{R}_e \cap S_q} \left(D^e_i - \overline{D^e}_{[q]}\right)^2,$$

where $\overline{D^e}_{[q]} = \frac{1}{u_{e[q]}} \sum_{i \in \mathcal{R}_e \cap S_q} D^e_i$. Therefore, similarly to (A.13), the Neyman variance estimator of $\widehat{DE}^{aug}_{adj}$ is

$$\widehat{V}_{Neyman}\left(\widehat{DE}^{aug}_{adj}\right) = \sum_{q=0,1} \frac{u^2_{e[q]}}{u^2_e} \widehat{V}_{\boldsymbol{Z}}\left(\widehat{DE}^{aug}_{adj[q]}\right). \tag{A.14}$$

We apply the same conservative contamination-attributed variance correction (A.12) derived for the adjusted estimator, adapting it to the augmented residuals. Specifically, we compute the conservative contamination-attributed variance estimator (A.11) for each fold, and combine it similarly to (A.13).

From Lu et al. (2025, Theorem 4), under common regularity conditions, both estimators $\widehat{IE}^{aug}_{adj}$ and $\widehat{DE}^{aug}_{adj}$ are approximately normal and normal-based confidence intervals using the variance estimators achieve a valid (asymptotic) coverage rate.

In addition, the random splitting minimizes the dependence between the two folds. While strict independence (w.r.t. $\boldsymbol{Z}$) implies no interference between the splits, the contamination between ego-networks is assumed to be sparse. Consequently, the dependence of the estimated outcome models on the treatment assignments of units in the complementary fold is expected to be negligible. Under this approximation, the estimated outcome models at each split can be treated as fixed w.r.t. the design $\boldsymbol{Z}$ (Lin, 2013; Lu et al., 2025). Therefore, the derivations shown in Sections A.3-A.4 imply that $\widehat{IE}^{aug}_{adj}$ and $\widehat{DE}^{aug}_{adj}$ remain approximately unbiased estimators of IE and DE, respectively, provided that the number of edges connecting units in the two folds (ego–ego and alter–ego) is small relative to the sample size.

A.9 Relative risk estimand and estimators

In the case of binary outcomes, we can also consider the relative risk (RR) estimand

$$IE^{RR} = \frac{\sum_{i \in \mathcal{R}_a} Y_i(0,1)}{\sum_{i \in \mathcal{R}_a} Y_i(0,0)}. \tag{A.15}$$

Analogously to (3), the estimator based on the observed data is therefore

$$\widehat{IE}^{RR} = \frac{\sum_{i \in \mathcal{R}_a} \frac{\mathbb{I}\{\widetilde{F}_i = 1\} Y_i}{p_z}}{\sum_{i \in \mathcal{R}_a} \frac{\mathbb{I}\{\widetilde{F}_i = 0\} Y_i}{1-p_z}}. \tag{A.16}$$

We saw in (A.1) that the numerator in (A.16) is an unbiased estimator of the numerator in (A.15). However, from (A.3) the denominator is biased. Rearranging (A.3),

$$\frac{1-p_z}{1-\pi^a_i} \mathbb{E}_{\boldsymbol{Z}}\left[\frac{\mathbb{I}\{\widetilde{F}_i = 0\} Y_i}{1-p_z}\right] = Y_i(0,0) + \frac{\pi^a_i - p_z}{1-\pi^a_i} Y_i(0,1).$$

Thus, using (A.1), we can unbiasedly estimate $Y_i(0,0)$ through

$$\frac{1-p_z}{1-\pi^a_i} \frac{\mathbb{I}\{\widetilde{F}_i = 0\} Y_i}{1-p_z} - \frac{\pi^a_i - p_z}{1-\pi^a_i} \frac{\mathbb{I}\{\widetilde{F}_i = 1\} Y_i}{p_z}.$$

That motivates the adjusted estimator

$$\widehat{IE}_{adj}^{RR} = \frac{\sum_{i \in \mathcal{R}_a} \frac{\mathbb{I}\{\widetilde{F}_i=1\} Y_i}{p_z}}{\sum_{i \in \mathcal{R}_a} \frac{1-p_z}{1-\pi_i^a} \frac{\mathbb{I}\{\widetilde{F}_i=0\} Y_i}{1-p_z} - \frac{\pi_i^a - p_z}{1-\pi_i^a} \frac{\mathbb{I}\{\widetilde{F}_i=1\} Y_i}{p_z}}. \tag{A.17}$$

Being a ratio estimator, (A.17) is biased, but the bias can be easily bounded (Särndal et al., 2003).

A.10 Relaxing Assumption 4

We provide two approaches to relax Assumption 4, which states that the empirical covariance between exposure probabilities π_i^e and the direct effect among exposed egos $\Delta_i(1)$ is equal to the empirical covariance between π_i^e and the direct effect among non-exposed egos $\Delta_i(0)$, that is,

$$S_{\boldsymbol{\pi}^e, \boldsymbol{\Delta}(1)} = S_{\boldsymbol{\pi}^e, \boldsymbol{\Delta}(0)}.$$

In the first approach, we derive sharp bounds for DE as a function of the sensitivity parameters π_i^e and κ and the observed data. That enables researchers to obtain bounds, instead of point estimates, for DE, or obtain bounds for DE given estimates of π_i^e and κ from validation data, as described in Section 3.6.

In the second approach, we modify the sample average proportionality expressed in (7), to a stronger assumption of unit-level proportionality. That produces a modified bias-corrected estimator for DE that is an unbiased estimator without requiring Assumption 4 to hold.

Bounds for DE without Assumption 4. We can relax Assumption 4 by using bounds instead of an unbiased estimator given values of π_i^e and κ. Note that we can write

$$S_{\boldsymbol{\pi}^e, \boldsymbol{\Delta}(1)} - S_{\boldsymbol{\pi}^e, \boldsymbol{\Delta}(0)} = S_{\boldsymbol{\pi}^e, \boldsymbol{\Delta}(1) - \boldsymbol{\Delta}(0)}.$$

Therefore, Assumption 4 is equivalent to assuming $S_{\boldsymbol{\pi}^e, \boldsymbol{\Delta}(1) - \boldsymbol{\Delta}(0)} = 0$. Note that from (A.9) the expectation of the naive HT estimator is

$$\begin{aligned} \mathbb{E}_{\boldsymbol{Z}}\left[\widehat{DE}\right] &= \left[1 + \overline{\pi}^e(\kappa - 1)\right] DE + S_{\boldsymbol{\pi}^e, \boldsymbol{\Delta(1)}} - S_{\boldsymbol{\pi}^e, \boldsymbol{\Delta(0)}} \\ &= \left[1 + \overline{\pi}^e(\kappa - 1)\right] DE + S_{\boldsymbol{\pi}^e, \boldsymbol{\Delta}(1) - \boldsymbol{\Delta}(0)} \end{aligned}$$

By the Cauchy-Schwarz inequality, we have

$$|S_{\boldsymbol{\pi}^e, \boldsymbol{\Delta}(1) - \boldsymbol{\Delta}(0)}| \leq S_{\boldsymbol{\pi}^e} S_{\boldsymbol{\Delta}(1) - \boldsymbol{\Delta}(0)},$$

where the first component $S_{\boldsymbol{\pi}^e}$ is the empirical standard deviation of π_i^e and can be computed given a specific value of the sensitivity parameters. The second standard deviation can be bounded as follows. Assume that there exist two constants $b_0 \leq b_1$ such that $b_0 \leq Y_i(z, f) \leq b_1$ for all i, z, f. Then $|\Delta_i(f)| \leq b_1 - b_0$ and $|\Delta_i(1) - \Delta_i(0)| \leq 2(b_1 - b_0)$. Therefore, by the Bhatia–Davis inequality, a sharp bound for the variance $S^2_{\boldsymbol{\Delta}(1) - \boldsymbol{\Delta}(0)}$ is

$$\begin{aligned} S^2_{\boldsymbol{\Delta}(1) - \boldsymbol{\Delta}(0)} &\leq \left[2(b_1 - b_0) - (\overline{\Delta}(1) - \overline{\Delta}(0))\right] \left[\overline{\Delta}(1) - \overline{\Delta}(0) + 2(b_1 - b_0)\right] \\ &= 4(b_1 - b_0)^2 - \left(\overline{\Delta}(1) - \overline{\Delta}(0)\right)^2. \end{aligned}$$

But from (7), we can write $\overline{\Delta}(1) - \overline{\Delta}(0) = (\kappa - 1)\overline{\Delta}(0) = (\kappa - 1)DE$. Thus, the variance bound is

$$S^2_{\mathbf{\Delta}(1)-\mathbf{\Delta}(0)} \leq 4(b_1 - b_0)^2 - [(\kappa - 1)DE]^2 .$$

That yields a bound for the standard deviation

$$S_{\mathbf{\Delta}(1)-\mathbf{\Delta}(0)} \leq \sqrt{4(b_1 - b_0)^2 - [(\kappa - 1)DE]^2} \leq 2(b_1 - b_0),$$

as $[(\kappa - 1)DE]^2 \geq 0$. Therefore, we obtain that the empirical covariance $S_{\boldsymbol{\pi}^e,\mathbf{\Delta}(1)-\mathbf{\Delta}(0)}$ is bounded by

$$|S_{\boldsymbol{\pi}^e,\mathbf{\Delta}(1)-\mathbf{\Delta}(0)}| \leq 2(b_1 - b_0)S_{\boldsymbol{\pi}^e}.$$

Thus, we can bound DE as follows,

$$\frac{\mathbb{E}_{\boldsymbol{Z}}\left[\widehat{DE}\right] - 2(b_1 - b_0)S_{\boldsymbol{\pi}^e}}{1 + \overline{\pi}^e(\kappa - 1)} \leq DE \leq \frac{\mathbb{E}_{\boldsymbol{Z}}\left[\widehat{DE}\right] + 2(b_1 - b_0)S_{\boldsymbol{\pi}^e}}{1 + \overline{\pi}^e(\kappa - 1)},$$

or, since $\mathbb{E}_{\boldsymbol{Z}}\left[\widehat{DE}_{adj}\right] = \frac{\mathbb{E}_{\boldsymbol{Z}}\left[\widehat{DE}\right]}{1+\overline{\pi}^e(\kappa-1)}$, we can write the bounds in terms of the bias-corrected estimator

$$\mathbb{E}_{\boldsymbol{Z}}\left[\widehat{DE}_{adj}\right] - \frac{2(b_1 - b_0)S_{\boldsymbol{\pi}^e}}{1 + \overline{\pi}^e(\kappa - 1)} \leq DE \leq \mathbb{E}_{\boldsymbol{Z}}\left[\widehat{DE}_{adj}\right] + \frac{2(b_1 - b_0)S_{\boldsymbol{\pi}^e}}{1 + \overline{\pi}^e(\kappa - 1)}.$$

For example, for binary outcomes, $b_0 = 0, b_1 = 1$, and we obtain

$$\frac{\mathbb{E}_{\boldsymbol{Z}}\left[\widehat{DE}\right] - 2S_{\boldsymbol{\pi}^e}}{1 + \overline{\pi}^e(\kappa - 1)} \leq DE \leq \frac{\mathbb{E}_{\boldsymbol{Z}}\left[\widehat{DE}\right] + 2S_{\boldsymbol{\pi}^e}}{1 + \overline{\pi}^e(\kappa - 1)}.$$

Where in practice it is possible to use the naive estimator $\widehat{DE}$ instead of its expectation in the bounds and construct confidence intervals for the partially identified parameter DE (e.g., as proposed by Imbens and Manski, 2004).

Unit-level proportionality assumption. Recall that in the main text we defined κ as the proportion between the sample average direct effects among exposed egos $\overline{\Delta}(1)$ to the effect among non-exposed egos $\overline{\Delta}(0)$. By the design-based inference framework, this proportionality is only a notation and holds by design. The bias-corrected estimator $\widehat{DE}_{adj}$, as defined in (8), is unbiased estimator of DE only given additional conditions, as formally expressed in Proposition 3. Here, replacing Assumption 4 with a different assumption produce a modified bias-corrected estimator.

Specifically, assume that instead of constant κ that link $\Delta_i(1)$ and $\Delta_i(0)$ on average over the egos ($\overline{\Delta}(1) = \kappa\overline{\Delta}(0)$), there is a constant κ^* that control the unit-level proportionality

Assumption A.1 (Unit-level proportionaliity). *There exist a constant κ^* such that $\Delta_i(1) = \kappa^*\Delta_i(0)$ for all $i \in \mathcal{R}_e$.*

That is, Assumption A.1 states that $Y_i(1,1) - Y_i(0,1) = \kappa^* \{Y_i(1,0) - Y_i(0,0)\}$ for all egos. The interpretation of the additional sensitivity parameter κ in Assumption A.1 is related to the interaction between treatment and exposure in the potential outcomes of the egos. Under the saturated potential outcomes model

$$Y_i(z, f) = \beta_{0i} + \beta_{1i}z + \beta_{2i}f + \beta_{3i}zf,$$

Assumption A.1 is equivalent to $\beta_{3i} = (\kappa^* - 1)\beta_{1i}$. That is, the interaction term β_{3i} is proportional to the direct effect term β_{1i} for all egos. Thus, while κ in (7) expressed an *average-level* relation, κ^* in Assumption A.1 posit *unit-level* proportionality. However, for binary potential outcomes, Assumption A.1 implies only a specific set of possible values for κ^*, and not any constant value, thereby restricting the valid values to specify for κ^* when running the sensitivity analysis described below.

We can use a modified bias-corrected direct effect estimator

$$\widehat{DE}^*_{adj} = \frac{1}{n_e} \sum_{i \in \mathcal{R}_e} \frac{1}{1 + \pi_i^e(\kappa^* - 1)} \left[\frac{\mathbb{I}\{Z_i = 1\}Y_i}{p_z} - \frac{\mathbb{I}\{Z_i = 0\}Y_i}{1 - p_z} \right].$$

Since both κ^* and π_i^e are fixed and therefore independent of $\boldsymbol{Z}$, we can use (A.5), (A.6), and (A.7) to obtain

$$\begin{aligned}
\mathbb{E}\left[\widehat{DE}^*_{adj}\right] &= \frac{1}{n_e} \sum_{i \in \mathcal{R}_e} \frac{1}{1 + \pi_i^e(\kappa^* - 1)} \left[\pi_i^e \{Y_i(1,1) - Y_i(0,1)\} + (1 - \pi_i^e)\{Y_i(1,0) - Y_i(0,0)\}\right] \\
&\overset{(i)}{=} \frac{1}{n_e} \sum_{i \in \mathcal{R}_e} \frac{1}{1 + \pi_i^e(\kappa^* - 1)} \left[\pi_i^e \kappa^* \{Y_i(1,0) - Y_i(0,0)\} + (1 - \pi_i^e)\{Y_i(1,0) - Y_i(0,0)\}\right] \\
&= \frac{1}{n_e} \sum_{i \in \mathcal{R}_e} [Y_i(1,0) - Y_i(0,0)] \\
&= DE,
\end{aligned}$$

where in (i) we used Assumption A.1. Therefore, $\widehat{DE}^*_{adj}$ is an unbiased estimator of DE under Assumptions 1-3 and Assumption A.1 instead of assuming homogeneous exposure probabilities or Assumption 4 in Proposition 3.

The estimation of variance for $\widehat{DE}^*_{adj}$ can be performed similarly to the estimator described in Section A.7 by replacing r_i^e with $r_i^{e*} = \frac{1}{1+\pi_i^e(\kappa^*-1)} \left[\frac{\mathbb{I}\{Z_i=1\}Y_i}{p_z} - \frac{\mathbb{I}\{Z_i=0\}Y_i}{1-p_z} \right]$ such that $\widehat{DE}^*_{adj} = \frac{1}{n_e} \sum_{i \in \mathcal{R}_e} r_i^{e*}$. In addition, outcome-augmented estimation for $\widehat{DE}^*_{adj}$ is also similar to the methods described in Section A.8 for $\widehat{DE}_{adj}$.

Running the sensitivity analysis (GSA or PBA) with this modified estimator follows the same logic described in Section 3.5, by replacing κ with κ^*, albeit with a different interpretation, of course.

In addition, Assumption A.1 can be relaxed by allowing conditional proportionality given covariates $\boldsymbol{X}_i$. That is, replacing κ^* with $\kappa_i^* = \kappa^*(\boldsymbol{X}_i)$ in Assumption A.1 enables researchers to specify some structure on the proportionality instead of assuming the same level of homogeneity for all units. For example, for a categorical covariate $X_{i1} \in \{1, 2, 3\}$, researchers can specify κ_1^* for $X_{i1} = 1$ and set $\kappa_k^* = \tau_k \kappa_1^*$ for constant τ_k for $k = 2, 3$. This relaxes the homogeneous proportionality by allowing each subgroup (determined by X_{i1}) to have different values. For instance, X_{i1} can represent some proxy of risky behavior or susceptibility to be influenced by peer advice, such as age group. In that case, researchers can treat κ_1^* as the sensitivity parameters and either assume fixed values for τ_k or instead specify multiple values for τ_k as well. That can be easily done in PBA, where researchers only need to specify a distribution for these parameter values.

A.11 Estimating the sensitivity parameters with internal validation data

Alters. Assume we have an internal validation subsample of alters $\mathcal{V}_a \subset \mathcal{R}_a$ that are asked to recall information about the intervention. Assume that alters who recall such

information have a true exposure $F_i = 1$ as they were exposed to at least one treated ego. This essentially assumes that the recall data in the validation subset perfectly equate the true exposure status. This might not hold if recall is not perfect, but can be strengthened through stringent selection criteria for the validation subsample (Chao et al., 2025). The conditional probability of true exposure given observed non-exposure for alters $\Pr(F_i = 1 \mid \widetilde{F}_i = 0, i \in \mathcal{R}_a)$ is linked to the marginal exposure probability π_i^a through (A.2):

$$\pi_i^a = p_z + (1 - p_z) \Pr(F_i = 1 \mid \widetilde{F}_i = 0, i \in \mathcal{R}_a).$$

Assuming homogeneous exposure probabilities $\pi_i^a = \pi^a$, we can estimate this conditional probability in validation subsample through

$$\widehat{q}^a = \frac{\sum_{i \in \mathcal{V}_a} \mathbb{I}\left\{F_i = 1, \widetilde{F}_i = 0\right\}}{\sum_{i \in \mathcal{V}_a} \mathbb{I}\left\{\widetilde{F}_i = 0\right\}},$$

and use $\widehat{q}^a$ to estimate π^a

$$\widehat{\pi}^a = p_z + (1 - p_z)\widehat{q}^a.$$

Given $\widehat{\pi}^a$, we can estimate the latent ego-alter edges probabilities ρ^a as well. By Example 1, $\pi^a = p_z + (1 - p_z)\left[1 - (1 - p_z\rho^a)^{n_e - 1}\right]$. Substituting π^a with $\widehat{\pi}^a$ and solving for ρ^a yields

$$\widehat{\rho}^a = \frac{1 - (1 - \widehat{q}^a)^{\frac{1}{n_e - 1}}}{p_z}.$$

A similar derivation can yield an estimate of m^a, the expected number of missing ego-alter edges (Example 2).

Under heterogeneous exposure probabilities, we can regress F_i on $\widetilde{F}_i = 0$ and $\boldsymbol{X}_i$ among the validation subsample to learn $\Pr(F_i = 1 \mid \widetilde{F}_i = 0, \boldsymbol{X}_i, i \in \mathcal{V}_a)$ and use the learned model to predict the exposure probabilities for the other alters in the main study. In this case, it is impossible to infer the edge probabilities ρ_{ij}^a as π_i^a is a convolution of these non-identical values. However, given the estimated π_i^a values, researchers can still use the bias-adjusted indirect effect estimator (5). Accounting for uncertainty in estimating π_i^a in the final indirect effect estimates can be achieved using standard methods from the measurement error literature (e.g., as described by Chao et al., 2025).

Egos. For egos, we separate between two types of internal validation data gathered on a validation subsample $\mathcal{V}_e \subset \mathcal{R}_e$ of egos

1. Asking egos to recall *any* information related to the intervention.

2. Asking egos if they heard or learned about the intervention from other friends or sources.

The first type is similar to the validation data described for the alters. The second is more directly related to indirect exposure to treatment through neighbors.

Treated egos ($Z_i = 1$) in the validation subset $\mathcal{V}_e$ of the first type should recall any information related to the intervention, as they were randomized to participate in it. However, non-treated egos ($Z_i = 0$) would have learned about the intervention only through their relations to other treated egos. Thus, any non-treated ego that recalled information about the intervention in the validation subsample can be viewed as having been truly exposed to

at least one treated ego, that is, $F_i = 1$. By the experimental design (Assumption 1) and the exposure mapping specification (Assumption 2), the treatment status Z_i is independent of the exposure F_i. Therefore, $\pi_i^e = \Pr(F_i = 1 \mid i \in \mathcal{R}_e) = \Pr(F_i = 1 \mid Z_i = 0, i \in \mathcal{R}_e)$. Under homogeneous exposure probabilities $\pi_i^e = \pi^e$ we can estimate π_i^e from the validation subsample through

$$\widehat{q}^e = \widehat{\Pr}(F_i = 1 \mid Z_i = 0, i \in \mathcal{V}_e) = \frac{\sum_{i \in \mathcal{V}_e} \mathbb{I}\{F_i = 1, Z_i = 0\}}{\sum_{i \in \mathcal{V}_e} \mathbb{I}\{Z_i = 0\}}.$$

Similarly for the derivation given to the alters, we can use $\widehat{q}^e$ to estimate ρ^e (in Example 1) or m^e (in Example 2). We can also estimate heterogeneous exposure probabilities by learning $\Pr(F_i = 1 \mid Z_i = 0, \boldsymbol{X}_i, i \in \mathcal{V}_e)$.

In the second validation data type, we have access to the true exposure status F_i of all egos in the validation subsample. Thus, we can learn π^e or π_i^e from the entire validation subsample, instead of only non-treated egos, as described above. Moreover, κ can also be learned on this validation subsample, given that researchers are willing to assume that the validation subsample is representative of the full sample and does not suffer from selection challenges. Recall that $\kappa = \frac{\overline{\Delta}(1)}{\overline{\Delta}(0)}$. Therefore, an estimator of κ from the validation subsample is

$$\widehat{\kappa} = \frac{\overline{Y}^{\mathcal{V}_e}(1,1) - \overline{Y}^{\mathcal{V}_e}(0,1)}{\overline{Y}^{\mathcal{V}_e}(1,0) - \overline{Y}^{\mathcal{V}_e}(0,0)},$$

where $\overline{Y}^{\mathcal{V}_e}(z,f) = \frac{\sum_{i \in \mathcal{V}_e} \mathbb{I}\{Z_i = z, F_i = f\} Y_i}{\sum_{i \in \mathcal{V}_e} \mathbb{I}\{Z_i = z, F_i = f\}}$, is the average outcome in the validation subsample for egos with treatment $z \in \{0,1\}$ and exposure $f \in \{0,1\}$. Given the estimators of π_i^e and κ from the validation subset, we can either obtain point estimates of DE using $\widehat{DE}^{adj}$ or have bounds for DE, as described in Appendix A.10.

B Extension to three-level exposure mapping

B.1 Preliminaries

We extend the framework to a three-level exposure mapping for alters, $F_i \in \{0, 1, 2+\}$, indicating exposure to zero, exactly one, or two-or-more treated neighbors, respectively. We consider only the indirect effect estimand and estimators. We derive bias expressions for the naive HT estimator and propose an adjusted estimator to correct for the bias. The adjusted estimators rely on the same sensitivity parameter of alter–ego edges probabilities ρ_{ij}^a (12) defined in Section 3.4. However, we require an additional sensitivity parameter, similar to the unit-level interaction parameter κ^* for the bias-corrected direct effect estimator presented in Appendix A.10.

Let $S_i = \sum_{j \neq i} Z_j A_{ij}$ be the true number of treated neighbors of unit i in the population network $\boldsymbol{A}$. For alters $i \in \mathcal{R}_a$, we can write $S_i = \sum_{j \in \mathcal{R}_e} Z_j A_{ij}$. We first modify the exposure mapping (Assumption 2).

Assumption B.1 (Three-Level Exposure Mapping)**.** *Let*

$$F(\boldsymbol{Z}_{-i}, \boldsymbol{A}) = \begin{cases} 0, & S_i = 0 \\ 1, & S_i = 1 \\ 2+, & S_i \geq 2 \end{cases}$$

For any $\boldsymbol{z}, \boldsymbol{z}' \in \mathcal{Z}_{\mathcal{R}}$, if $z_i = z_i'$ and $F(\boldsymbol{z}_{-i}, \boldsymbol{A}) = F(\boldsymbol{z}'_{-i}, \boldsymbol{A})$, then $Y_i(\boldsymbol{z}) = Y_i(\boldsymbol{z}')$.

By Assumption B.1, we can write $Y_i(\boldsymbol{Z}) = Y_i(Z_i, F_i)$, where $Z_i \in \{0,1\}$ and $F_i \in \{0,1,2+\}$. Thus, each unit now has six potential outcomes, instead of four. The consistency assumption (Assumption 3) is also modified.

Assumption B.2 (Consistency (Three-Level))**.** *The observed outcomes are given by*

$$Y_i = \sum_{z\in\{0,1\}} \sum_{f\in\{0,1,2+\}} Y_i(z,f)\mathbb{I}\left\{Z_i = z, F_i = f\right\}.$$

We focus on the estimand of the sample average indirect effect of exposure to a single versus no treated neighbors (1)

$$IE = \frac{1}{n_a}\sum_{i\in\mathcal{R}_a} Y_i(0,1) - Y_i(0,0).$$

B.2 Bias of the naive estimator

The naive HT estimator $\widehat{IE}$ remains as defined in (3), as the observed exposures $\widetilde{F}_i$ are zero or one. Its expectation is $\mathbb{E}_{\boldsymbol{Z}}\left[\widehat{IE}\right] = \frac{1}{n_a}\sum_{i\in\mathcal{R}_a}(E_{i,1} - E_{i,0})$, where

$$E_{i,1} = \mathbb{E}_{\boldsymbol{Z}}\left[\frac{\mathbb{I}\{\widetilde{F}_i = 1\}Y_i}{p_z}\right]$$
$$E_{i,0} = \mathbb{E}_{\boldsymbol{Z}}\left[\frac{\mathbb{I}\{\widetilde{F}_i = 0\}Y_i}{1-p_z}\right].$$

The issue is that observed exposures $\widetilde{F}_i = 1$ can correspond to true exposures of either $F_i = 1$ or $F_i = 2+$, and observed exposures $\widetilde{F}_i = 0$ can correspond to true exposures of $F_i = 0$, $F_i = 1$, or $F_i = 2+$. Therefore, the observed outcomes Y_i under observed exposures $\widetilde{F}_i$ are mixtures of potential outcomes under different true exposures F_i.

Define $S_{i,-e(i)} = \sum_{j\in\mathcal{R}_e^{e(i)}} Z_j A_{ij}$ to be the number of treated neighbors of alter i *other than* its own ego $e(i)$. This represents the possible exposure from contamination between the ego-networks.

To derive the bias, we first write $E_{i,0}$ and $E_{i,1}$ as functions of the potential outcomes and the exposure probabilities, which in turn are used to obtain an expression for $\mathbb{E}_{\boldsymbol{Z}}\left[\widehat{IE}\right]$.

Derivation of $E_{i,1}$: If $\widetilde{F}_i = 1$, then $Z_{e(i)} = 1$. The true number of treated neighbors is $S_i = 1 + S_{i,-e(i)}$. Given that $Z_{e(i)} = 1$,

- The true exposure is $F_i = 1$ if $S_i = 1$, which implies $S_{i,-e(i)} = 0$.
- The true exposure is $F_i = 2+$ if $S_i \geq 2$, which implies $S_{i,-e(i)} \geq 1$.

By Assumptions B.1-B.2,

$$\begin{aligned}
E_{i,1} &= \frac{1}{p_z}\mathbb{E}_{\boldsymbol{Z}}\left[\mathbb{I}\{\widetilde{F}_i = 1, F_i = 1\}Y_i(0,1) + \mathbb{I}\{\widetilde{F}_i = 1, F_i = 2+\}Y_i(0,2+)\right] \\
&= \frac{1}{p_z}\mathbb{E}_{\boldsymbol{Z}}\left[\mathbb{I}\{Z_{e(i)} = 1, S_{i,-e(i)} = 0\}Y_i(0,1) + \mathbb{I}\{Z_{e(i)} = 1, S_{i,-e(i)} \geq 1\}Y_i(0,2+)\right] \\
&= \Pr(S_{i,-e(i)} = 0)Y_i(0,1) + \Pr(S_{i,-e(i)} \geq 1)Y_i(0,2+).
\end{aligned}$$

The last step holds because $S_{i,-e(i)}$ is independent of $Z_{e(i)}$ under Assumption 1, and $\Pr(Z_{e(i)} = 1) = p_z$.

Derivation of $E_{i,0}$: If $\widetilde{F}_i = 0$, then $Z_{e(i)} = 0$. The true number of treated neighbors is $S_i = S_{i,-e(i)}$. Given that $Z_{e(i)} = 0$,

- The true exposure is $F_i = 0$ if $S_i = 0$, which implies $S_{i,-e(i)} = 0$.
- The true exposure is $F_i = 1$ if $S_i = 1$, which implies $S_{i,-e(i)} = 1$.
- The true exposure is $F_i = 2+$ if $S_i \geq 2$, which implies $S_{i,-e(i)} \geq 2$.

Therefore,

$$\begin{aligned} E_{i,0} &= \frac{1}{1-p_z}\mathbb{E}_{\boldsymbol{Z}}\left[\sum_{f\in\{0,1,2+\}} \mathbb{I}\{\widetilde{F}_i = 0, F_i = f\}Y_i(0,f)\right] \\ &= \Pr(S_{i,-e(i)} = 0)Y_i(0,0) + \Pr(S_{i,-e(i)} = 1)Y_i(0,1) + \Pr(S_{i,-e(i)} \geq 2)Y_i(0,2+). \end{aligned}$$

Exposures probabilities. As in Section 3.4, the sensitivity model assumes conditional edge independence, with ρ^a_{ij} defined in (12). The random variable $S_{i,-e(i)}$ follows a Poisson-Binomial distribution with $n_e - 1$ probabilities $\{p_z\rho^a_{ij}\}_{j\in\mathcal{R}_e^{e(i)}}$. Let $\pi^*_{i,k} = \Pr(S_{i,-e(i)} = k)$. The required probabilities are

$$\begin{aligned} \pi^*_{i,0} &= \Pr(S_{i,-e(i)} = 0) = \prod_{j\in\mathcal{R}_e^{e(i)}} (1 - p_z\rho^a_{ij}) \\ \pi^*_{i,1} &= \Pr(S_{i,-e(i)} = 1) = \sum_{j\in\mathcal{R}_e^{e(i)}} \left[p_z\rho^a_{ij} \prod_{l\in\mathcal{R}_e^{e(i)}\setminus\{j\}} (1 - p_z\rho^a_{il})\right] \\ \pi^*_{i,2+} &= \Pr(S_{i,-e(i)} \geq 2) = 1 - \pi^*_{i,0} - \pi^*_{i,1}. \end{aligned}$$

For large n_e, analytic computation of $\pi^*_{i,1}$ can be challenging, but approximation methods exist (Hong, 2013).

Bias expression. First, note that $\Pr(S_{i,-e(i)} \geq 1) = 1 - \Pr(S_{i,-e(i)} = 0) = 1 - \pi^*_{i,0}$. Substituting these probabilities into the expressions we obtained above for $E_{i,0}$ and $E_{i,1}$, the expectations of the two components of the naive estimator are:

$$\begin{aligned} E_{i,1} &= \pi^*_{i,0}Y_i(0,1) + (1 - \pi^*_{i,0})Y_i(0,2+) \\ E_{i,0} &= \pi^*_{i,0}Y_i(0,0) + \pi^*_{i,1}Y_i(0,1) + (1 - \pi^*_{i,0} - \pi^*_{i,1})^*Y_i(0,2+) \end{aligned}$$

The expectation of the naive estimator is:

$$\begin{aligned} \mathbb{E}_{\boldsymbol{Z}}\left[\widehat{IE}\right] &= \frac{1}{n_a}\sum_{i\in\mathcal{R}_a} (E_{i,1} - E_{i,0}) \\ &= \frac{1}{n_a}\sum_{i\in\mathcal{R}_a} \left[-\pi^*_{i,0}Y_i(0,0) + (\pi^*_{i,0} - \pi^*_{i,1})Y_i(0,1) + \pi^*_{i,1}Y_i(0,2+)\right] \\ &= IE + \frac{1}{n_a}\sum_{i\in\mathcal{R}_a} \left[\pi^*_{i,1}\left\{Y_i(0,2+) - Y_i(0,1)\right\} - (1 - \pi^*_{i,0})\left\{Y_i(0,1) - Y_i(0,0)\right\}\right], \end{aligned} \tag{B.1}$$

which implies that the naive estimator is also biased for this indirect effect estimand. The bias depends on the average of the unit-level indirect effect of 2+ exposures relative to 1 exposures and of 1 exposures relative to 0 exposures, weighted by the exposure probabilities $\pi^*_{i,1}$ and $1 - \pi^*_{i,0}$, respectively.

B.3 Bias-corrected estimator

The resulting expected value (B.1) is a linear combination of the unknown potential outcomes $\{Y_i(0,0), Y_i(0,1), Y_i(0,2+)\}$. The problem is that the observed data do not provide enough information to identify all three potential outcomes for each alter $i \in \mathcal{R}_a$. To achieve identification, we introduce an additional sensitivity parameter δ via a unit-level structural assumption on the potential outcomes, analogous to Assumption A.1 of unit-level proportionality for the egos.

Assumption B.3. *There exists a constant δ such that $Y_i(0,2+)-Y_i(0,1) = \delta\{Y_i(0,1) - Y_i(0,0)\}$ for all alters $i \in \mathcal{R}_a$.*

The sensitivity parameter δ in Assumption B.3 provides an intuitive way to model the dose-response relationship of alter exposure. It is best interpreted through the *marginal effect of additional exposure*. The effect of first exposure is $Y_i(0,1) - Y_i(0,0)$. The *additional* effect of the second-and-higher exposure is $Y_i(0,2+) - Y_i(0,1)$. Assumption B.3 implies a direct relationship. Researchers can therefore specify plausible values for δ based on their domain knowledge.

- $\delta = 0$. The effect *saturates* after one exposure, and any further contamination is irrelevant. This recovers the binary exposure model.
- $\delta > 0$. There is a *reinforcing* cumulative dose effect, where a second exposure adds benefit. For example, $\delta = 1$ implies the same marginal effect between first and second-and-higher exposure. However, $\delta \in (0,1)$ implies that the marginal effect between first and zero exposure is higher than the marginal effect between second-or-higher and first exposure.
- $\delta < 0$. Additional exposures *diminish* the effect of the first. For example, exposure to more than one treated neighbor may create confusion or mistrust, reducing the benefit of exposure.

This framework allows researchers to conduct sensitivity analysis by specifying a plausible range for the marginal effect of exposures, rather than the absolute value of $Y_i(0,2+)$ (e.g., by deriving sharp, yet informative, bounds). Rearranging the statement in Assumption B.3, we have

$$Y_i(0,2+) = (\delta+1)Y_i(0,1) - \delta Y_i(0,0). \tag{B.2}$$

Using (B.2), the expectation in (B.1) can be written as,

$$\mathbb{E}_{\boldsymbol{Z}}\left[\widehat{IE}\right] = \frac{1}{n_a}\sum_{i\in\mathcal{R}_a}\left[\pi^*_{i,0} + \pi^*_{i,1}\delta\right]\left[Y_i(0,1) - Y_i(0,0)\right], \tag{B.3}$$

which implies the bias-corrected estimator of indirect effect under the three-level exposures

$$\widehat{IE}_{adj} = \frac{1}{n_a}\sum_{i\in\mathcal{R}_a}\frac{1}{\pi^*_{i,0} + \pi^*_{i,1}\delta}\left[\frac{\mathbb{I}\{\widetilde{F}_i = 1\}Y_i}{p_z} - \frac{\mathbb{I}\{\widetilde{F}_i = 0\}Y_i}{1-p_z}\right]. \tag{B.4}$$

Following the same strategy as in the proof of Proposition 2, and utilizing the derivations above, it can be shown that the adjusted estimator $\widehat{IE}_{adj}$ is unbiased for the indirect effect estimand IE

$$\mathbb{E}_{\boldsymbol{Z}}\left[\widehat{IE}_{adj}\right] = IE.$$

Moreover, (B.3) implies that the naive estimator $\widehat{IE}$ is unbiased if

- $\pi_{i,0}^* = 1$. This corresponds to no contamination between ego-networks (no additional treated neighbors beyond the ego). That is equivalent to $\rho_{ij}^a = 0$ for all alter–ego edges.
- For all $i \in \mathcal{R}_a$, we have that $\delta = \frac{1-\pi_{i,0}^*}{\pi_{i,1}^*} = 1 + \frac{\pi_{i,2+}^*}{\pi_{i,1}^*}$. This implies that the marginal effect of additional exposure is exactly offset by the contamination between ego-networks.

B.4 Relation to the two-level exposure model

Note that if we set $\delta = 0$ in (B.2), then $Y_i(0,2+) = Y_i(0,1)$, and the additional exposure level 2+ collapses into level 1. Recall that $\pi_i^a = \Pr(F_i = 1 \mid i \in \mathcal{R}_a)$ is the probability that alter i is exposed to *at least* one treated neighbor under the two-level exposure mapping. We can write

$$\begin{aligned}1 - \pi_i^a &= \Pr(F_i = 0 \mid Z_{e(i)} = 1 \in \mathcal{R}_a)\Pr(Z_{e(i)} = 1 \mid i \in \mathcal{R}_a) \\ &\quad + \Pr(F_i = 0 \mid Z_{e(i)} = 0 \in \mathcal{R}_a)\Pr(Z_{e(i)} = 0 \mid i \in \mathcal{R}_a) \\ &= \Pr(S_{i,-e(i)} = 0)(1 - p_z) \\ &= \pi_{i,0}^*(1 - p_z).\end{aligned}$$

That is, $\pi_{i,0}^* = \frac{1-\pi_i^a}{1-p_z}$. Substituting this into (B.4) with $\delta = 0$, we recover the bias-corrected estimator for the two-level exposure mapping given in (5). Therefore, the two-level exposure model can be viewed as a special case of the three-level exposure model with saturation of effect after the first exposure.

B.5 Direction of bias, sign preservation, and effect magnitude

If $Y_i(0,1) - Y_i(0,0) \geq 0$ for all alters $i \in \mathcal{R}_a$, then

$$\begin{cases} \mathbb{E}_{\boldsymbol{Z}}\left[\widehat{IE}\right] \geq IE \geq 0, & \delta \geq \max_{i\in\mathcal{R}_a}\left\{1 + \frac{\pi_{i,2+}^*}{\pi_{i,1}^*}\right\}, \\ 0 \leq \mathbb{E}_{\boldsymbol{Z}}\left[\widehat{IE}\right] \leq IE, & \delta \in \left(\max_{i\in\mathcal{R}_a}\left\{1 - \frac{1-\pi_{i,2+}^*}{\pi_{i,1}^*}\right\}, \min_{i\in\mathcal{R}_a}\left\{1 + \frac{\pi_{i,2+}^*}{\pi_{i,1}^*}\right\}\right), \\ \mathbb{E}_{\boldsymbol{Z}}\left[\widehat{IE}\right] \leq 0 \leq IE, & \delta \leq \min_{i\in\mathcal{R}_a}\left\{1 - \frac{1-\pi_{i,2+}^*}{\pi_{i,1}^*}\right\}. \end{cases}$$

Similarly, if $Y_i(0,1) - Y_i(0,0) \leq 0$ for all alters $i \in \mathcal{R}_a$, we obtain the opposite inequalities. Therefore, given monotonicity (in either direction) of the potential outcomes $Y_i(0,1)$ and $Y_i(0,0)$, the estimator $\widehat{IE}$ is biased away, towards, or changes sign relative to the true indirect effect IE, depending on the value of the sensitivity parameter δ.

Similarly to the augmented bias-corrected estimator (Section 3.3), we can define an augmented version of the three-level exposure bias-corrected estimator by adding outcome models $\widehat{\mu}_i^a(f)$ for $f \in \{0,1\}$. Variance estimation can be achieved using the same approaches described in Section A.7 for the bias-corrected indirect effect estimators under two-level exposures.

B.6 Sensitivity analysis

The bias-corrected estimator (B.4) can be used in the same way as the bias-corrected estimators under two-level exposure mapping described in Section 3.5. That is, based on their domain knowledge, researchers can specify plausible ranges for the sensitivity

parameters δ and the basic components that result in ρ_{ij}^a and conduct GSA or PBA. The main difference is the specification of the additional sensitivity parameter δ, and a slightly more complicated computation of the exposure probabilities $\pi_{i,0}^*$ and $\pi_{i,1}^*$. The latter can be easily computed in `R` using the approximations developed by Hong (2013), for example, as implemented in the `poisbinom` package.

C Probabilistic bias analysis

We provide a brief overview of the technical motivation behind PBA. For a more detailed treatment, we refer the reader to Greenland (2005) and Fox et al. (2021).

Assume researchers observe data $\boldsymbol{D}$ and are interested in estimating a parameter θ (e.g., a causal effect). They construct an estimator $\widehat{\theta}(\boldsymbol{D})$ based on the observed data, but know that it is biased. Namely,

$$\mathbb{E}\left[\widehat{\theta}(\boldsymbol{D})\right] \neq \theta.$$

Suppose there exists a vector of sensitivity or bias parameters ρ such that the researchers can construct an adjusted estimator $\widehat{\theta}_{adj}(\boldsymbol{D}, \rho)$ that is an unbiased estimator of θ given the true value of ρ:

$$\mathbb{E}\left[\widehat{\theta}_{adj}(\boldsymbol{D}, \rho) \mid \rho\right] = \theta.$$

However, the true values of ρ are typically unknown and not identifiable from the data. Standard GSA compares estimates of $\widehat{\theta}_{adj}(\boldsymbol{D}, \rho)$ (and possibly associated uncertainty quantification) across a fixed grid of ρ values, treating each point in the grid as if it were the truth. In contrast, PBA treats ρ as a random variable governed by a probability distribution $p(\rho)$. This distribution reflects the researcher's prior knowledge or uncertainty regarding the true values and structure of the bias.

The goal of PBA is to estimate the "posterior" distribution of the parameter of interest θ, accounting for both the uncertainty in ρ and the sampling variability of the estimator. This distribution can be approximated via Monte Carlo simulation (Greenland, 2005). First, we draw M samples $\rho^{(1)}, \ldots, \rho^{(M)}$ from the prior $p(\rho)$. For each draw $\rho^{(m)}$, we calculate the adjusted point estimate $\widehat{\theta}_{adj}(\boldsymbol{D}, \rho^{(m)})$ and its associated variance estimate, denoted $\widehat{V}(\boldsymbol{D}, \rho^{(m)})$. To fully propagate the statistical uncertainty, we simulate a value $\theta^{(m)}$ from the estimator's approximate sampling distribution, typically assumed to be normal:

$$\theta^{(m)} \sim N\left(\widehat{\theta}_{adj}(\boldsymbol{D}, \rho^{(m)}),\, \widehat{V}(\boldsymbol{D}, \rho^{(m)})\right).$$

The resulting values $\theta^{(1)}, \ldots, \theta^{(M)}$ approximate the marginal "posterior" distribution of the parameter of interest θ, accounting for both the uncertainty regarding the sensitivity parameters and the statistical uncertainty arising from the data. This distribution can be summarized through standard summary statistics (e.g., the mean and percentiles).

In our context, the only randomness in the data is due to the randomization $\boldsymbol{Z}$. Thus, $\widehat{\theta}$ corresponds to the naive estimators $\widehat{IE}$ or $\widehat{DE}$. The sensitivity ρ consists of the edge probabilities ρ_{ij}^a or ρ_{ij}^e and the interaction parameter κ. The adjusted estimators $\widehat{\theta}_{adj}$ correspond to $\widehat{IE}_{adj}$ or $\widehat{DE}_{adj}$. This formulation yields the PBA procedure described in Section 3.5.

D Simulations

Code for replicating the simulation study and data analysis is available at https://github.com/barwein/ENRT_SA.

We generated three covariates for each unit.

$$\begin{aligned} X_{i1} &\sim \text{Bernoulli}(p_1), \\ X_{i2} &\sim \text{Bernoulli}(p_2), \\ X_{i3} &\sim \text{Normal}(0,1), \end{aligned}$$

where $p_1 = 0.6, p_2 = 0.2$ for egos and $p_1 = 0.5, p_2 = 0.3$ for alters. The covariates coefficients in the potential outcome models were set to $\boldsymbol{\beta}_e = (-0.5, -0.3, 0.2)$ for egos and $\boldsymbol{\beta}_a = (-0.4, -0.2, 0.1)$ for alters. Heterogeneous edge probabilities were generated with weights computed using the Euclidean distance $\|\cdot\|_2$ between the covariates of units.

Tables D.1 and D.2 report the simulation results for the indirect and direct effects, respectively, with homogeneous exposure probabilities and augmented estimators. All results are qualitatively similar to those presented in the main text.

Table D.3 shows the results of the direct effect estimators under heterogeneous exposure probabilities (with $m_e = 150$) under misspecification of κ values. That is, the true value is $\kappa = 1.5$ while we tested how using $\kappa = 0.5$ and $\kappa = 1.1$ impacts the estimates. The table shows that using $\kappa = 0.5$ severely affects the bias-corrected estimators $\widehat{DE}_{adj}$ and $\widehat{DE}_{adj}^{aug}$ by increasing the bias and reducing the coverage compared to the naive HT estimator $\widehat{DE}$. That is the case since using $\kappa < 1$ when the true value is larger than one, changes the direction of the bias correction in the weights used in (8), resulting in inferior performance to the naive estimator. However, using a misspecified value closer to the true value $\kappa = 1.1$ (which is still in the same direction $\kappa > 1$ as the true value), negatively impacts the bias and coverage of the bias-corrected estimators, but still results in better performance than the naive estimator.

Table D.4 shows the results of the direct effect estimators under heterogeneous exposure probabilities (with $m_e = 150$) with violation of Assumption 4. The violation was simulated by modifying the data-generating process of the potential outcomes. Specifically, we have added a heterogeneous interaction term such that the potential outcomes for egos $i \in \mathcal{R}_e$ are now generated from

$$Y_i(z,f) = -0.5 + 2z + 0.5f + zf + \beta_4 zfX_{i1} + \boldsymbol{X}_i^T\boldsymbol{\beta}_e + \varepsilon_i.$$

Taking $\beta_4 = 0$ yields the original data-generating process used in the other setups. However, taking $\beta_4 \neq 0$ results in heterogeneous treatment and exposure interactions, leading to violation of Assumption 4. Table D.4 shows the results with $\beta_4 = 0.5$ and $\beta_4 = 1.1$, while using the true κ value in the bias-corrected estimators. Larger β_4 leads to stronger violation of Assumption 4. The table shows that the violation of Assumption 4 only slightly affects the bias-corrected estimators regardless of their specification and β_4 values. Specifically, the bias was minimal, but the augmented estimators tend to underestimate the variance ($SD/SE > 1$).

Finally, Tables D.5 and D.6 show the results for the indirect and direct effects, respectively, with binary potential outcomes under homogeneous or heterogeneous exposure probabilities with $m_a = 100$ and $m_e = 150$. The binary potential outcomes were sampled from a logistic model with

$$\begin{aligned} \operatorname{logit}\Pr(Y_i(z,f)=1) &= -0.5 + 2z + 0.5f + zf + \boldsymbol{X}_i^T\boldsymbol{\beta}_e, \quad && \text{for } i \in \mathcal{R}_e, \\ \operatorname{logit}\Pr(Y_i(0,f)=1) &= -0.5 + 2f + \boldsymbol{X}_i^T\boldsymbol{\beta}_a, && \text{for } i \in \mathcal{R}_a, \end{aligned}$$

using the same values for $\boldsymbol{\beta}_e$ and $\boldsymbol{\beta}_a$ as described above. We targeted the DE and IE on the difference scale, but the outcome-augmented estimators used the predicted probabilities from a logistic regression model. The results are qualitatively similar to those with continuous potential outcomes, showing that the bias-corrected estimators performed better than the naive estimators.

Table D.1: Simulation results for indirect effect with homogeneous exposure probabilities. True effect is $IE = 2$.

Scenario	Specification	Augmented	Bias	Coverage	SD/SE
$m^a = 100$	Heterogeneous	FALSE	0.000	0.963	0.941
		TRUE	-0.001	0.954	0.986
	Homogeneous	FALSE	-0.001	0.962	0.942
		TRUE	-0.002	0.954	0.986
	Naive	FALSE	-0.236	0.506	0.942
		TRUE	-0.237	0.400	0.986
$m^a = 200$	Heterogeneous	FALSE	0.008	0.972	0.881
		TRUE	0.006	0.954	0.962
	Homogeneous	FALSE	0.003	0.973	0.882
		TRUE	0.001	0.956	0.959
	Naive	FALSE	-0.440	0.063	0.882
		TRUE	-0.442	0.020	0.959

Table D.2: Simulation results for direct effect with homogeneous exposure probabilities. True effect is $DE = 2$.

Scenario	Specification	Augmented	Bias	Coverage	SD/SE
$m^e = 150, \kappa = 1.5$	Heterogeneous	FALSE	0.012	0.984	0.798
		TRUE	0.008	0.956	0.961
	Homogeneous	FALSE	0.000	0.982	0.810
		TRUE	-0.003	0.955	0.969
	Naive	FALSE	0.529	0.247	1.079
		TRUE	0.525	0.132	1.052
$m^e = 250, \kappa = 1.5$	Heterogeneous	FALSE	0.026	0.999	0.583
		TRUE	0.023	0.980	0.827
	Homogeneous	FALSE	0.007	0.999	0.600
		TRUE	0.004	0.978	0.843
	Naive	FALSE	0.724	0.076	1.070
		TRUE	0.720	0.012	1.031

Table D.3: Simulation results for direct effect with heterogeneous exposure probabilities and misspecified κ values. True effect is $DE = 2$, true $\kappa = 1.5$, and $m_e = 150$.

Scenario	Specification	Augmented	Bias	Coverage	SD/SE
Using $\kappa = 0.5$	Heterogeneous	FALSE	1.385	0.008	0.755
		TRUE	1.376	0.000	0.951
	Homogeneous	FALSE	1.417	0.006	0.772
		TRUE	1.408	0.000	0.959
	Naive	FALSE	0.514	0.279	1.068
		TRUE	0.508	0.175	1.045
Using $\kappa = 1.1$	Heterogeneous	FALSE	0.392	0.716	0.791
		TRUE	0.385	0.430	0.952
	Homogeneous	FALSE	0.388	0.697	0.813
		TRUE	0.382	0.429	0.960
	Naive	FALSE	0.514	0.279	1.068
		TRUE	0.508	0.175	1.045

Table D.4: Simulation results for direct effect with heterogeneous exposure probabilities and violation of Assumption 4. True effect is $DE = 2$ and $m_e = 150$. The value β_4 controls the magnitude of violation, where larger values correspond to larger violations. Values of κ depend on β_4 and are computed from the potential outcomes.

Scenario	Specification	Augmented	Bias	Coverage	SD/SE
$\beta_4 = 0.5$ and $\kappa = 1.68$	Heterogeneous	FALSE	0.003	0.984	0.792
		TRUE	-0.002	0.955	0.969
	Homogeneous	FALSE	-0.010	0.981	0.815
		TRUE	-0.016	0.952	0.977
	Naive	FALSE	0.681	0.122	1.094
		TRUE	0.673	0.049	1.063
$\beta_4 = 1.5$ and $\kappa = 1.97$	Heterogeneous	FALSE	0.009	0.980	0.814
		TRUE	0.003	0.950	0.993
	Homogeneous	FALSE	-0.010	0.976	0.840
		TRUE	-0.015	0.949	1.000
	Naive	FALSE	1.013	0.020	1.134
		TRUE	1.004	0.005	1.087

Table D.5: Simulation results for indirect effect with binary potential outcomes under homogeneous or heterogeneous exposure probabilities with $m_a = 100$. Augmented estimators used a logistic regression model for the augmentation. True effect is $IE = 0.4775$.

Scenario	Specification	Augmented	Bias	Coverage	SD/SE
Heterogeneous	Heterogeneous	FALSE	0.001	0.968	0.913
		TRUE	0.001	0.984	0.789
	Homogeneous	FALSE	0.001	0.967	0.913
		TRUE	0.001	0.985	0.788
	Naive	FALSE	-0.055	0.928	0.913
		TRUE	-0.055	0.834	0.788
Homogeneous	Heterogeneous	FALSE	-0.003	0.968	0.912
		TRUE	-0.003	0.987	0.786
	Homogeneous	FALSE	-0.003	0.967	0.912
		TRUE	-0.003	0.986	0.785
	Naive	FALSE	-0.059	0.920	0.912
		TRUE	-0.059	0.804	0.785

Table D.6: Simulation results for direct effect with binary potential outcomes under homogeneous or heterogeneous exposure probabilities with $m_e = 150$. Augmented estimators used a logistic regression model for the augmentation. True effect is $DE = 0.52$ and $\kappa = 0.932$.

Scenario	Specification	Augmented	Bias	Coverage	SD/SE
Heterogeneous	Heterogeneous	FALSE	-0.005	0.994	0.674
		TRUE	-0.006	0.982	0.812
	Homogeneous	FALSE	-0.004	0.993	0.692
		TRUE	-0.005	0.981	0.818
	Naive	FALSE	-0.023	0.954	0.939
		TRUE	-0.024	0.963	0.886
Homogeneous	Heterogeneous	FALSE	-0.012	0.996	0.675
		TRUE	-0.012	0.987	0.772
	Homogeneous	FALSE	-0.011	0.994	0.689
		TRUE	-0.012	0.986	0.778
	Naive	FALSE	-0.029	0.953	0.934
		TRUE	-0.030	0.964	0.845

E Data analysis

Figure E.1 shows the distribution of the number of alters per ego-network in the data. The average and median number is 2, the range is from 1 to 6.

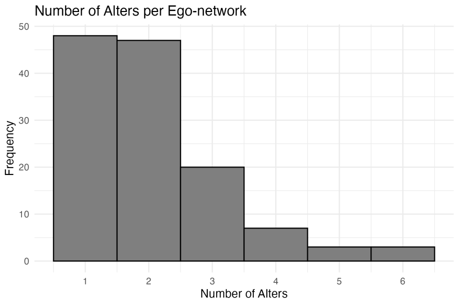

Figure E.1: Distribution of number of alters per ego-network.

Figure E.2 complements Figure 3 and shows the the GSA results (Section 5.1) for the direct effect using the augmented estimators given that $\kappa = 1.5$. We also included the results using $\gamma^e = 2$, representing a stronger influence of covariates' similarity on the exposure probabilities π_i^e. This presentation, similar to that of the indirect effect, enables us to see how changing the expected number of missing ego–ego edges (m^e) impacts the estimated direct effect. Clearly, for all specifications, the estimated effects were almost identical and approached zero as the contamination level increased Furthermore, Figure E.3 shows the results of GSA with the non-augmented estimators $\widehat{DE}_{adj}$, also including $\gamma^e = 2$. The figure shows that under all three specifications, the bias-corrected estimates are similar. Figure E.4 complements Figure E.2 by showing the bias-corrected estimates with the non-augmented estimators for $\kappa = 1.5$. This figure highlights the similarity of the results across the different edge probability specifications. Finally, Figure E.5 shows the augmented bias-corrected DE estimates as a function of κ given the value $\widehat{m}^e = 33.4$ estimated from the internal validation data, showing that increasing κ yields bias-corrected estimates closer to zero.

Figure E.6 shows the results of the GSA for indirect effect using the non-augmented estimators $\widehat{IE}_{adj}$. The structure is similar to that of the non-augmented estimator presented in the main text (Figure 2). However, the augmented estimator had values closer to zero for all levels of contamination (including no contamination) and edge probability specifications. Taking $\gamma^e = 2$ results in higher variance estimates for large levels of missing alter–ego edges ($m^a \geq 250$).

Figures E.7 and E.8 show the results of the PBA for indirect and direct effect using the non-augmented estimators $\widehat{IE}_{adj}$ and $\widehat{DE}_{adj}$. The results are similar to those of the non-augmented estimators presented in the main text (Figure 4).

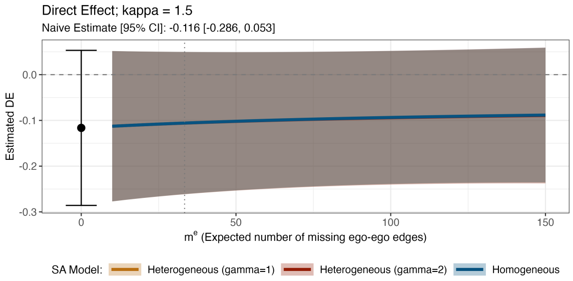

Figure E.2: GSA for direct effect with $\kappa = 1.5$ using the augmented estimators $\widehat{DE}_{adj}^{aug}$. The dashed vertical line at 33.4 is the estimate of m^e from the internal validation data.

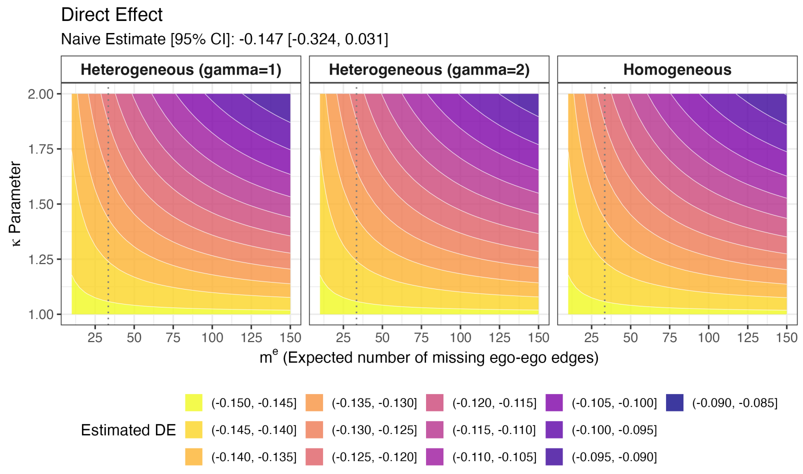

Figure E.3: GSA for direct effect with non-augmented estimators $\widehat{DE}_{adj}$. The dashed vertical line at 33.4 is the estimate of m^e from the internal validation data.

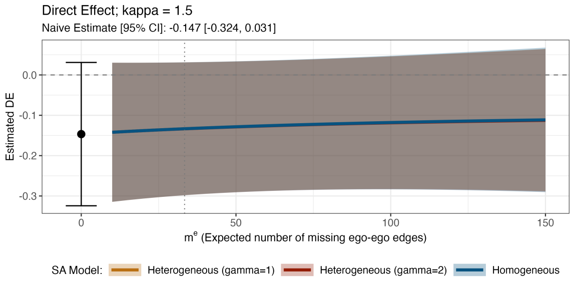

Figure E.4: GSA for direct effect with $\kappa = 1.5$. Not-augmented estimator. The dashed vertical line at 33.4 is the estimate of m^e from the internal validation data.

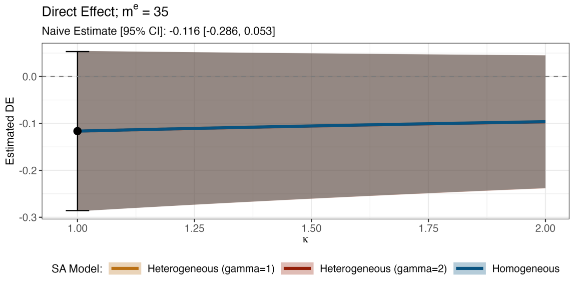

Figure E.5: GSA for direct effect with augmented estimators using $m^e = 35$ to approximate the bias-corrected estimates obtained using the the estimated value $\widehat{m}^e = 33.4$ from the internal validation data.

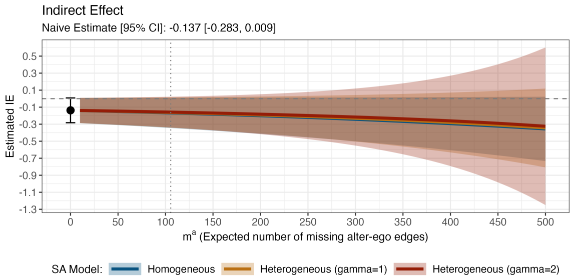

Figure E.6: GSA for indirect effect with non-augmented estimators $\widehat{IE}_{adj}$. The dashed vertical line at 105.5 is the estimate of m^a from the internal validation data.

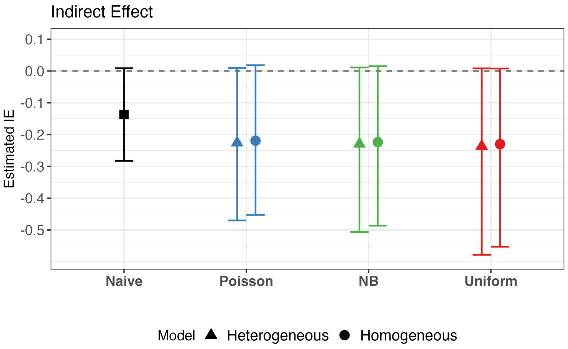

Figure E.7: PBA for indirect effect with non-augmented estimators $\widehat{IE}_{adj}$. Results are shown as mean (95% intervals) of bias-corrected estimates across $B = 10^4$ Monte Carlo samples. The results account for both bias and statistical uncertainty.

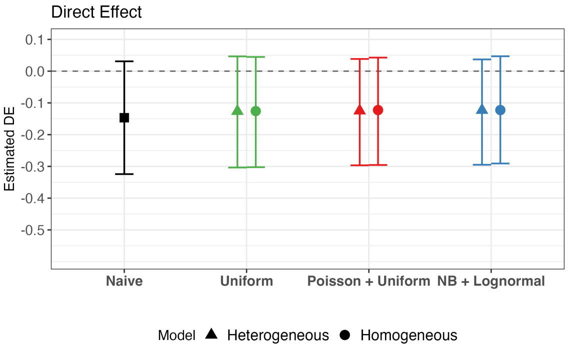

Figure E.8: Probabilistic bias analysis for direct effect with non-augmented estimators $\widehat{DE}_{adj}$. Results are shown as mean (95% intervals) of bias-corrected estimates across $B = 10^4$ Monte Carlo samples. The results account for both bias and statistical uncertainty.

F Results of the example analysis code

Analysis code applied to a simulated dataset similar to the design of HPTN 037 can be found at https://github.com/barwein/ENRT_SA. To replicate it, run the script `ENRT_example_code.R`. We provide here the results from running the code, in the order of their appearance in the script.

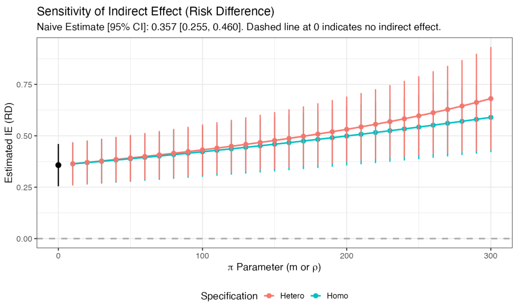

Figure F.1: Results for IE estimand with GSA in the ENRT example code.

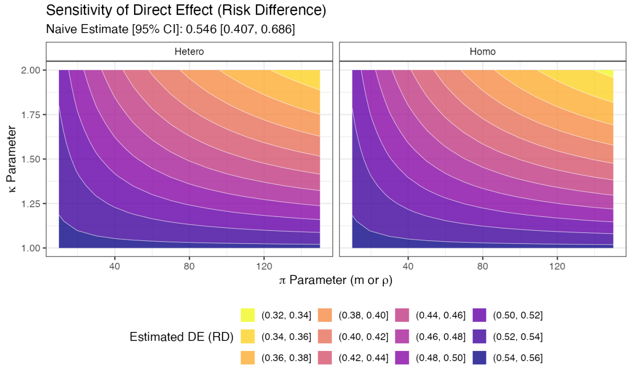

Figure F.2: Results for DE estimand with GSA in the ENRT example code.

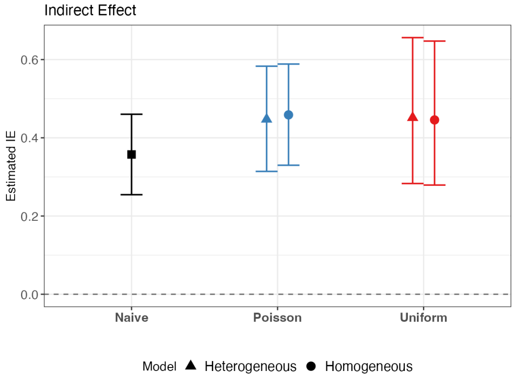

Figure F.3: Results for IE estimand with PBA in the ENRT example code.

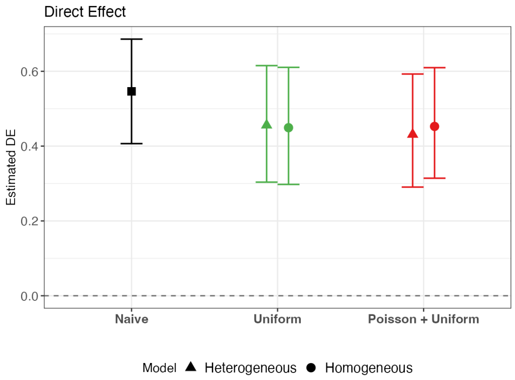

Figure F.4: Results for DE estimand with PBA in the ENRT example code.

References

Baldi, P. and Rinott, Y. (1989). On normal approximations of distributions in terms of dependency graphs. *The Annals of Probability*, 17(4):1646–1650.

Chao, A., Spiegelman, D., Buchanan, A., and Forastiere, L. (2025). Estimation and inference for causal spillover effects in egocentric-network randomized trials in the presence of network membership misclassification. *Biostatistics*, 26(1):kxaf009.

Ding, P. (2024). *A first course in causal inference*. Chapman and Hall/CRC.

Fox, M. P., MacLehose, R. F., and Lash, T. L. (2021). *Applying quantitative bias analysis to epidemiologic data*, volume 10. Springer.

Greenland, S. (2005). Multiple-Bias Modelling for Analysis of Observational Data. *Journal of the Royal Statistical Society Series A: Statistics in Society*, 168(2):267–306.

Hong, Y. (2013). On computing the distribution function for the poisson binomial distribution. *Computational Statistics & Data Analysis*, 59:41–51.

Imbens, G. W. and Manski, C. F. (2004). Confidence intervals for partially identified parameters. *Econometrica*, 72(6):1845–1857.

Lin, W. (2013). Agnostic notes on regression adjustments to experimental data: Reexamining freedman's critique. *The Annals of Applied Statistics*, pages 295–318.

Lu, X., Shi, L., Liu, H., and Ding, P. (2025). Conditional cross-fitting for unbiased machine-learning-assisted covariate adjustment in randomized experiments. *arXiv preprint arXiv:2508.15664*.

Särndal, C.-E., Swensson, B., and Wretman, J. (2003). *Model Assisted Survey Sampling (Springer Series in Statistics)*. Springer.
